# Synthesis, Enzymatic Degradation, and Polymer-Miscibility Evaluation of Nonionic Antimicrobial Hyperbranched Polyesters with Indole or Isatin Functionalities

**DOI:** 10.1021/acs.biomac.1c00343

**Published:** 2021-04-26

**Authors:** Xiaoya Li, Sedef İlk, Javier A. Linares-Pastén, Yang Liu, Deepak Bushan Raina, Deniz Demircan, Baozhong Zhang

**Affiliations:** † Centre for Analysis and Synthesis, Department of Chemistry, Lund University, P.O. Box 124, SE-22100 Lund, Sweden; ‡ Faculty of Medicine, Department of Immunology, Niğde Ömer Halisdemir University, 51240 Niǧde, Turkey; § School of Engineering Sciences in Chemistry, Biotechnology and Health, Department of Chemistry, Division of Glycoscience, KTH Royal Institute of Technology, SE-10691 Stockholm, Sweden; ∥ Division of Biotechnology, Department of Chemistry, Lund University, P.O. Box 124, 22100 Lund, Sweden; ⊥ Faculty of Medicine, Department of Clinical Sciences, Orthopedics, Lund University, 22100 Lund, Sweden

## Abstract

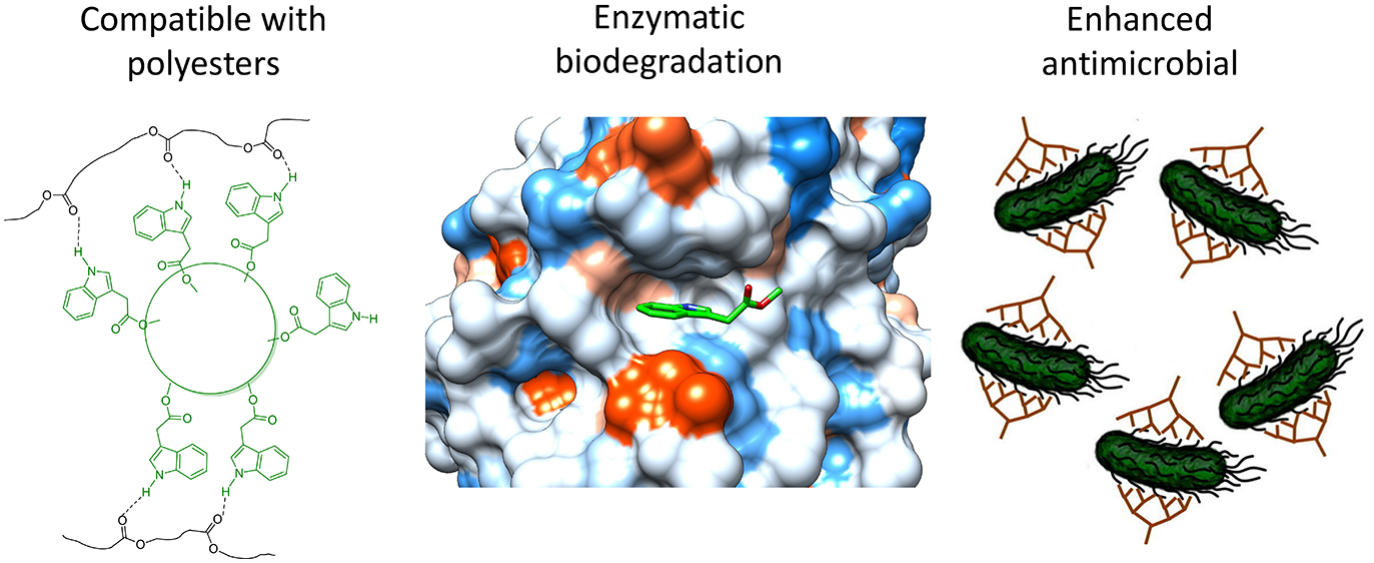

Most macromolecular antimicrobials are ionic and thus lack miscibility/compatibility with nonionic substrate materials. In this context, nonionic hyperbranched polyesters (HBPs) with indole or isatin functionality were rationally designed, synthesized, and characterized. Antimicrobial disk diffusion assay indicated that these HBPs showed significant antibacterial activity against 8 human pathogenic bacteria compared to small molecules with indole or isatin groups. According to DSC measurements, up to 20% indole-based HBP is miscible with biodegradable polyesters (polyhydroxybutyrate or polycaprolactone), which can be attributed to the favorable hydrogen bonding between the N–H moiety of indole and the C=O of polyesters. HBPs with isatin or methylindole were completely immiscible with the same matrices. None of the HBPs leaked out from plastic matrix after being immersed in water for 5 days. The incorporation of indole into HBPs as well as small molecules facilitated their enzymatic degradation with PETase from *Ideonella sakaiensis*, while isatin had a complex impact. Molecular docking simulations of monomeric molecules with PETase revealed different orientations of the molecules at the active site due to the presence of indole or isatin groups, which could be related to the observed different enzymatic degradation behavior. Finally, biocompatibility analysis with a mammalian cell line showed the negligible cytotoxic effect of the fabricated HBPs.

## 1 Introduction

Macromolecular antimicrobials (antimicrobial polymers, AMPs) have received growing attention because of their potential to overcome antimicrobial resistance,^1−5^ as well as other advantages like low leaching potential, high selectivity and efficacy, low skin permeation, and toxicity.^6−11^ In particular, the large size and low migration (leaching) potential of AMPs are desirable for the design of coatings or additives for clinical materials (e.g., catheters, scaffold, stitches, implants), of which microbial infection is a serious threat.^12−17^ Currently, most reported AMPs are ionic,^18^ which usually lack compatibility (or miscibility) with a nonionic matrix (e.g., polymers).^19^ This could lead to undesirable phase separation and inferior mechanical properties. Furthermore, ionic AMPs may suffer from undesirable high water solubility and ecotoxicity.^20−22^ These challenges can be potentially solved by the development of nonionic AMPs.

Due to the lack of ionic interactions with microbials (as most ionic APMs do), nonionic AMPs need particular functional groups (e.g., chlorine, organotin, carbon-rich esters) to endow antimicrobial properties.^2,7,23^ Considering the health and environmental impacts, those naturally existing antimicrobial functional groups have received growing attention for the design and synthesis of new nonionic AMPs (e.g., indole, isatin, anisole, curcumin, astaxanthin, tropolone, aspirin, limonene, etc.).^24−32^ These naturally occurring functionalities have been used and adapted by natural ecosystems, so they are prone to be more biodegradable and less ecotoxic.

Dendrimers and hyperbranched polymers (HBPs) form a particularly attractive class of AMPs^33−37^ because of their locally concentrated functionalities^38−40^ and enhanced interaction with a bacterial membrane.^41−48^ Today, most reported antimicrobial dendrimers and HBPs are cationic.^18^ For example, hyperbranched poly(amino-ester)s with quaternary ammoniums showed antibacterial activity when blended with polycaprolactone (PCL, a biodegradable polymer).^49^ Lucine-based hyperbranched or star-shaped polymers also exhibited remarkable antibacterial activity.^50^ Nonionic antimicrobial dendrimers or HBPs have been rarely reported. For instance, HBPs with indole and imidazole groups exhibited significant antibacterial properties and have been investigated as surface coating materials.^24^ Recently, our group reported that biobased HBPs with nonionic isatin and anisole groups showed antibacterial activity against 9 different pathogenic bacteria.^26^ However, their rigid molecular structures and high glass transition temperatures (*T*_g_ > 200 °C) limited their miscibility with low-*T*_*g*_ biodegradable polymers (e.g., PLA, PHB, PBS, etc.). In addition, these HBPs are nonbiodegradable, as many aliphatic hyperbranched polyesters are.^51,52^

This work aims to enhance the biodegradability of nonionic antibacterial HBPs as well as to improve their miscibility with biodegradable plastic matrices. To achieve these goals, new HBPs with flexible biobased aliphatic polyester backbones and naturally existing antimicrobial functional groups (i.e., indole, isatin) were designed. In particular, the presence of N–H as hydrogen-bond donor in indole is expected to enhance the miscibility with hydrogen-bond-accepting polyester matrices. Herein, we report the synthesis of new nonionic HBPs by grafting isatin or indole units onto a hydroxyl-terminated commercially available polyester HBP, Boltorn.^53,54^ The molecular structures, thermal properties, antimicrobial effects, enzymatic degradation,^55,56^ cytotoxicity, leaching potential in water, and miscibility with biodegradable polyesters were evaluated. The impact of indole and isatin groups on enzymatic degradation of aliphatic polyester backbones and small molecules was investigated. To our knowledge, this is the first report of nonionic dendritic AMPs that are plastics miscible and biodegradable.

## 2 Experimental Section

### 2.1 Chemicals and Materials

Boltorn H40 (98%) was purchased from Perstorp AB, Sweden. Isatin, methyl bromoacetate, potassium carbonate (K_2_CO_3_), 1-ethyl-3-(3′-dimethylaminopropyl) carbodiimide·HCl (EDC·HCl), 4-dimethylaminopyridine (DMAP), indole-3-acetic acid (**2**), *N*-methylindole-3-acetic acid, and methylindole-3-acetate were purchased from Sigma-Aldrich. Tetrahydrofuran (THF), *N*,*N*-dimethylformamide (DMF), *N*,*N*-dimethylacetamide (DMAc), 1,4-dioxane, chloroform, dichloromethane (DCM), dimethyl sulfoxide (DMSO), HCl, NaOH, ethanol, methanol, acetone, acetonitrile, ethyl acetate, diethyl ether, sodium carbonate (Na_2_CO_3_), NaHCO_3_ (ACS, Reag. Ph. Eur.), and NaSO_4_ were purchased from VWR Chemicals. Poly(3-hydroxybutyrate) (PHB) powder was supplied by BIOMER (Germany), which was washed with 0.001 N aqueous HCl for 30 min, washed with deionized water, and dried at 50 °C under vacuum for 2 days before further use. The acid-treated PHB had a weight-average molar mass (*M*_w_) of 620 000 g mol^–1^ and polydispersity index (PDI) of 2.0. PCL was purchased from Sigma-Aldrich with a weight-average molar mass (*M*_w_) of 14 000 g mol^–1^ and polydispersity index (PDI) of 1.4.

### 2.2 Synthesis

#### Methyl Isatin-*N*-acetate (**5**)

A solution of isatin **3** (5.30 g, 37.0 mmol, 1.0 equiv), methyl bromoacetate **4** (4.80 mL, 44.4 mmol, 1.2 equiv), and K_2_CO_3_ (7.68 g, 55.6 mmol, 1.5 equiv) in 80 mL of DMF was added into a 250 mL round-bottom flask and stirred at room temperature. After 16 h, the mixture was poured into ice–water, and the obtained orange precipitate was filtered, washed with water, and recrystallized from water to yield methyl isatin-*N*-acetate **5** as orange crystals (9.86 g, 76%). ^1^H NMR (400.13 MHz, DMSO-*d*_6_) δ, ppm: 7.69 (t, 1H, Ar), 7.62 (d, 1H, Ar), 7.22–7.16 (m, 2H, Ar), 4.64 (s, 2H, N–CH_2_–COOR), 3.71 (s, 3H, COOCH_3_). ^13^C NMR (100.61 MHz, DMSO-*d*_6_) δ, ppm: 183.06, 168.48, 158.61, 150.81, 138.94, 125.13, 124.16, 117.81, 111.57, 52.93, 41.55. Mp (DSC): 120.6 °C.

#### Isatin-*N*-acetic Acid (**1**)

To a well-stirred solution of **5** (0.50 g, 23 mmol) in ethanol/water (40 mL/10 mL) was added 10% NaOH solution (1.2 mL) dropwise in a 250 mL round-bottom flask equipped with a reflux condenser. The reaction mixture was stirred at 60 °C for 3 h until the color changed to yellow. Afterward, concentrated HCl was added dropwise, yielding an orange precipitate. The precipitate was filtered, dissolved in saturated Na_2_CO_3_ solution (100 mL), and washed with ethyl acetate (4 × 50 mL). Concentrated HCl was added into the collected aqueous phase to precipitate a yellow solid, isatin-*N*-acetic acid **1** (0.35 g, 70%). ^1^H NMR (400.13 MHz, DMSO-*d*_6_) δ, ppm: 7.69 (t, 1H, Ar), 7.61 (d, 1H, Ar), 7.22–7.14 (m, 2H, Ar), 4.53 (s, 2H, N–C*H*_*2*_–COOH). ^13^C NMR (100.61 MHz, DMSO-*d*_6_) δ, ppm: 183.47, 169.37, 158.60, 151.31, 138.95, 125.32, 124.05, 117.88, 111.54. Mp (DSC): 209.8 °C.

#### Isatin-Grafted BH40 (BISA)

A solution of BH40 (0.18 g, 0.060 mmol, 1.0 equiv), **1** (0.33 g, 1.6 mmol, 1.1 equiv), EDC·HCl (0.31 g, 1.6 mmol, 1.1 equiv), and DMAP (0.01 g, 5 wt %) in DMF (5 mL) in a capped 25 mL round-bottom flask was stirred at room temperature. After 18 h, the reaction mixture was added to a saturated NaHCO_3_ solution (200 mL). The resulting yellow precipitate was dissolved in acetone (2 mL), which was dropped into diethyl ether (100 mL) to precipitate. The resulting precipitate was collected by gravity filtration and dried at 50 °C under vacuum for 12 h to yield an orange solid as BISA (0.21 g, 42%). ^1^H NMR (400 MHz, DMSO-*d*_6_) δ, ppm: 7.70–7.49 (m, 2H, Ar), 7.61 (d, 1H, Ar), 7.21–7.03 (m, 2H), 4.99 (s, −O*H*), 4.61 (s, 2H, N–C*H*_*2*_–COOR), 4.30–3.94 (m, C*H*_*2*_OR), 3.53–3.39 (m, C*H*_2_OH), 1.25–0.87 (m, C*H*_*3*_R). ^13^C NMR (100.61 MHz, DMSO-*d*_6_) δ, ppm: 182.89, 174.19–166.97, 167.41, 158.54, 150.60, 138.82, 125.11, 124.17, 117.76, 111.39, 66.89–63.76, 51.26–45.79, 18.09–16.77.

#### Indole-Grafted BH40 (BIN)

A solution of BH40 (0.18 g, 0.060 mmol, 1.0 equiv), **2** (0.31 g, 1.6 mmol, 1.1 equiv), EDC·HCl (0.31 g, 1.6 mmol 1.1 equiv), and DMAP (0.01 g, 5 wt %) in DMF (5 mL) in a capped 25 mL round-bottom flask was stirred at room temperature. After 18 h, the reaction mixture was added to a saturated NaHCO_3_ solution (200 mL). The resulting brown precipitate was dissolved in acetone (2 mL), which was added into diethyl ether (100 mL). The resulting precipitate was collected by gravity filtration and dried at 50 °C under vacuum for 12 h to yield a brown solid as BIN (0.22 g, 45%). ^1^H NMR (400.13 MHz, DMSO-*d*_6_) δ, ppm: 10.91 (s, 1H, NH), 7.43(s, 1H, Ar), 7.32(s, 1H, Ar), 7.17(s, 1H, Ar), 7.04(s, 1H, Ar), 6.94 (s, 1H, Ar), 4.99 (s, O*H*), 4.23–3.94 (m, C*H*_*2*_OR), 3.68(s, 2H, ArC*H*_*2*_COOR), 3.59–3.39 (m, C*H*_*2*_OH), 1.18–0.89 (m, C*H*_*3*_R). ^13^C NMR (100.61 MHz, DMSO-*d*_6_) δ, ppm: 171.54, 174.03–171.25, 136.50, 127.44, 124.46, 121.50, 118.95, 118.84, 111.83, 107.26, 107.11, 66.38–63.72, 49.13–46.03, 30.78, 17.80–16.76.

### 2.3 Measurements

Nuclear magnetic resonance (NMR) spectra were recorded on a Bruker DRX400 spectrometer at a proton frequency of 400.13 MHz and a carbon frequency of 100.61 MHz. Fourier transform infrared (FT-IR) spectra were obtained with an attenuated total reflection (ATR) setup using a Bruker Alpha FT-IR spectrometer. Differential scanning calorimetry (DSC) measurements were performed using a TA Instruments DSC Q2000. The samples were studied with a heating rate of 10 °C min^–1^ under nitrogen with a purge rate of 50 mL min^–1^. The *T*_*g*_ was taken as the midpoint of the endothermic step change observed during the second heating run; the cool crystallization temperature *T*_c_ and melting temperature *T*_m_ were taken as those of main exo- and endothermal peaks, respectively. Thermogravimetric analysis (TGA) was performed under a nitrogen atmosphere with a Thermogravimetric Analyzer (TA Instrument Q500) at a heating rate 10 °C/min. Gel permeation chromatography (GPC) was carried out with three Shodex columns in series (KF-805, 2804, and 2802.5) and a refractive index (RI) detector (Viscotek model 250). All measurements were carried out at room temperature at a concentration of 3.0 mg mL^–1^ using chloroform as the eluent and at an elution rate of 1 mL min^–1^. Calibration was performed with four polystyrene standard samples (*M*_n_ = 650 kg mol^–1^ from Water Associates, 96 and 30 kg mol^–1^ from Polymer Laboratories, and 3180 g mol^–1^ from Agilent Technologies). A UV–vis spectrometer was used in the wavelength range from 200 to 600 nm with a resolution of 2 nm, employing quartz cuvettes of 10 mm path length. High-resolution mass spectrometry (HRMS) was performed by direct infusion on a Water Xevo-G2 QTOF mass spectrometer using electrospray ionization. Reversed phase liquid chromatography-mass spectrometry (LC-MS) was performed on a XEVO-G2 ESI-QToF mass spectrometer and Acquity UPLC equipped with an Acquity CSH C18 column (1.7 μm, 2.1 × 100 mm), all from Waters. The mobile phases contained 0.1% formic acid in water (A) and 0.1% formic acid in acetonitrile (B), and the gradient profile was 0.0–0.7 min 5% B, 0.7–8.0 min 5–99% B, followed by 99% B for 3 min. The column was kept at 60 °C, and the flow rate was 0.5 mL/min. Diode-array detection was performed between 190 and 300 nm, and the mass spectra with *m*/*z* between 50 and 1200 were generated in positive electrospray mode using a capillary voltage of 3 kV, cone voltage of 40 V, source temperature of 120 °C, desolvation temperature of 500 °C, cone gas of 50 L/h, and desolvation gas of 800 L/h (both N_2_). Lock mass correction was performed using leucine enkephalin (according to Waters standard recommendations). HPLC (high-performance liquid chromatography) measurements were carried out according to a previously developed method.^57^ A CMB-20A HPLC instrument (Shimadzu), with a UV–vis detector (SPD-20A) was used. A C18 column Kinetex 1.7 μm XB-C18 100 Å, LC column 50 × 2.1 mm was used with mobile phases A and B consisting of acetonitrile and 0.1% formic acid solution, respectively. The flow rate was fixed at 0.4 mL/min for 10 min.

### 2.4 Leaching Potential from PHB Films

First, films of pure PHB or PHB with 5 wt % HBP (BISA or BIN) or small molecules (isatin or indole-3-acetic acid) were prepared according to a solution-casting protocol.^26,58^ Pure PHB powder or mixtures of PHB powder with HBPs or small molecules were dissolved in chloroform at 100 °C for 5 min in a sealed vessel, followed by 5 h standing at room temperature without agitation. Afterward, the solutions were cast at room temperature in a Petri dish and dried for 24 h. For the evaluation of the leaching potential, the prepared PHB films (ca. 100 mg) were immersed in distilled water (10 mL). After 5 days, the UV absorbance of the water phase was measured.

### 2.5 PHB/HBP and PCL/HBP Blends Preparation

A powder of PCL with or without HBP additives (5–20 wt %) was dissolved in THF in a sealed vessel. The resulting homogeneous solutions stand for 5 h without any agitation. A powder of PHB with or without HBP additives (5–20 wt %) was dissolved in chloroform with a few drops of DMF at 100 °C for 5 min in a sealed vessel. The resulting homogeneous solutions were cooled to room temperature and allowed to stand for 5 h without any agitation. Afterward, the PCL or PHB solutions were cast at room temperate on a glass Petri dish and dried at 50 °C under vacuum for 48 h. The films were kept at room temperature until DSC measurements.

### 2.6 Production and Purification of Enzymes

PETase from *Ideonella sakaiensis* was produced in a recombinant strain of *Escherichia coli* as described in our previous work.^59,60^ Synthetic genes encoding the enzyme were chemically synthesized (GenScript USA Inc. Piscataway, NJ) with optimized codons for *E. coli*. PETase gene was inserted in a frame with an N-terminal histidine tag encoded by the backbone of the vector pET28b. The resulting construct was named pET28b::PETase and introduced into chemically competent *E. coli* 21(DE3) by thermic shock. The strain harboring pET28b::PETase was cultivated in LB medium with 34 μg/mL kanamycin. The recombinant strains were grown in 300 mL at 37 °C until an optical density of 0.6 was measured at a 600 nm wavelength. Thereafter, the production of recombinant enzyme was induced with 1 mM isopropyl β-d-1-thiogalactopyranoside at 25 °C for 12 h. The enzyme was purified by immobilized metal ion affinity chromatography (IMAC) according to the protocol previously reported.^67^ The purity of the prepared enzyme was analyzed by SDS-PAGE, and its concentration was determined spectrophotometrically at a 280 nm wavelength.

### 2.7 Enzymatic Reactions

#### Enzymatic Reactions with Polymers

Degradation reactions of polymers, BISA and BIN, with PETase were conducted according to our previously reported protocol.^59^ The polymers were soaked in a mixture of phosphate buffer (1000 μL, pH 7.0), water (750 μL), DMSO (200 μL), and PETase (50 μL, 2.10 mg mL^–1^). The reaction mixtures were incubated at 37 °C with shaking at 200 rpm for 72 h. In the meantime, nonenzymatic degradation of BISA and BIN was carried out by a negative control under identical conditions (no enzyme). After incubation, the samples were centrifuged at 13 000*g* for 10 min, and the supernatants were analyzed with LC-MS.

#### Enzymatic Reactions with Monomer

For the enzymatic reactions of monomers, methyl isatin-*N*-acetate (EISA) and methylindole-3-acetate (EIN), the monomers were soaked in a mixture of phosphate buffer (1000 μL, pH 7.0), water (700 μL), DMSO (200 μL), and PETase (100 μL, 0.62 mg mL^–1^). The reaction mixtures were incubated at 37 °C with shaking at 200 rpm. In the meantime, nonenzymatic degradation of EISA and EIN was determined by a negative control under identical conditions (without enzyme). The reactions were stopped at appropriate time intervals spread over 6 h by being kept at −80 °C until HPLC analysis, and the concentrations of the hydrolysis products were calculated.

#### Calibration

Standard solutions of EISA and EIN with six concentrations were used (0.25, 0.5, 1.0, 1.5, 2.0, and 2.5 g/L in DMSO) for calibration of the HPLC peak areas. A plot of the peak area versus the concentration of the standard solutions was made. Regression analysis was carried out. The correlation coefficient (*r*^2^), the slope of the line, the *Y* intercept, and line equation were calculated.

### 2.8 Molecular Docking

Molecular structures of ligands, EIN and EISA, were generated using the Avogadro program.^61^ Their geometries were optimized at the molecular-mechanics level with the force field MMFF94 and the algorithm Steepest Descend using 1000 steps and convergence of 10^–7^. The receptor was a crystallographic structure^62^ of PETase from *I. sakaiensis* available in the Protein Data Bank (PDB: 5XJH). Docking was performed thorough AutoDock^63^ implemented in YASARA v19.12.14 software.^64^ The molecular models were analyzed with Chimera.^65^

### 2.9 Bacteria Culture

Food-borne and human pathogenic microorganisms *Escherichia coli* ATCC 25922 (*Ec*), *Staphylococcus aureus* ATCC 25923 (*Sa*), *Proteus mirabilis* ATCC 14153 (*Pm*), *Proteus vulgaris* ATCC13315 (*Pv*), *Pseudomonas aeruginosa* ATCC27853 (*Pa*), *Enterobacter aerogenes* ATCC13048 (*Ea*), *Bacillus thuringiensis* (*Bt*), and *Streptococcus mutans* ATCC 25175 (*Sm*) were employed to evaluate the antibacterial properties of HBPs (BISA and BIN) and small molecular reagents (isatin or indole derivatives and gentamicin). All bacteria strains were subcultured on (Luria–Bertani) LB agar culture at 37 °C for 24 h.

### 2.10 Antimicrobial Disk Diffusion Assay

Disk-diffusion assay according to the modified standard method was applied to evaluate the antibacterial properties.^23,66^ First, the tested solid samples (HBPs or small molecular agents) were dissolved in DMF with four different concentrations (1, 0.5, 0.25, and 0.1 mg mL^–1^). Microorganisms’ susceptibility was adjusted with 0.5 McFarland as a reference standard. The prepared solutions were sterilized under UV light for 5 min before testing. Microorganism culture suspension (100 μL, 10^6^ cells per mL) was swabbed onto a plate within Müller–Hinton agar. Filter disks with a diameter of 6 mm were placed on the Petri plate inoculated with microorganisms, and 20 μL of the prepared sample solutions was loaded on the sterile disks. Afterward, bacteria cultures were incubated at 37 °C for 24 h. Disks containing gentamicin (10 μg per disk) or DMF (pure solvent) were used as a positive or negative control, respectively. All experiments were performed in triplicate. The results are expressed as the mean diameter of the inhibition zone in mm ± standard deviation (mean ± SD). Significant differences between the two groups were evaluated as *p* values by *t* test using Microsoft Excel software. *p* < 0.05 indicates a significant difference, while *p* ≥ 0.05 indicates an insignificant difference.

### 2.11 Anti-Quorum Sensing (Anti-QS)

Disc diffusion assay was performed to evaluate the anti-QS property of HBP and small molecules.^66^*Choromobacterium violaceum CV026* as the reporter strain was used to determine the anti-QS activity of HBPs (BISA and BIN) and two small molecules (EISA and EIN). Before the assay, the suspension of bacteria culture was incubated in Luria–Bertani (LB) broth at 30 °C for 24 h. For the anti-QS assay, the bacteria culture was swabbed onto the surface of LB soft agar (100 mL) containing signal molecule *N*-hexanoyl-l-homoserine lactone (HSL) (0.25 μg/mL) of CV026. Afterward, sterile discs loaded with solutions (20 μL) of the prepared samples (10 μg/mL) were placed on the Petri plates and incubated at 30 °C for 24 h. A commercial antibiotic gentamicin (10 μg per disc) and the pure solvent (DMF) were evaluated as a positive or negative control, respectively. The anti-QS activity was measured by the size of the formed turbid halo around the disc (in contrast to the purple background).

### 2.12 MTT Assay

The MG-63 osteoblast-like human cells were cultured in Dulbecco’s Modified Eagle Media (DMEM) supplemented with 10% fetal bovine serum (FBS), 1% penicillin, and 1% streptomycin in a humidified incubator at 37 °C. The medium was replaced every 2 days. Cells were trypsinized and centrifuged at 400*g* for 4 min to get a concentrated cell pellet when the confluence reached 80%; 1 ×10^4^ cells/well were seeded on a 96-well plate and cultured for 24 h before adding the materials. Test compounds ((1. C) control group; (2) BH40; (3) BISA; (4) BIN; (5) EISA; (6) EIN) dissolved in DMSO were added to the cell culture at a final DMSO concentration of 1% (v/v). Fresh culture medium without samples was used as positive control, and each sample was replicated in five wells. After being cultured for 24 h with materials from each group, the cell culture medium was discarded and the cells were washed with phosphate buffer once. MTT working solution (0.5 mg/mL) was added to the cells and incubated for 2 h at 37 °C, after which DMSO was added to the reaction products followed by further incubation for 10 min. The solubilized contents were pipetted and transferred into a clear bottom 96-well plate. Absorbance was determined by spectrophotometry at a 600 nm wavelength. Plain DMSO was used for blank subtraction.

## 3 Results and Discussion

### 3.1 Synthesis of HBPs

**Scheme 1:**
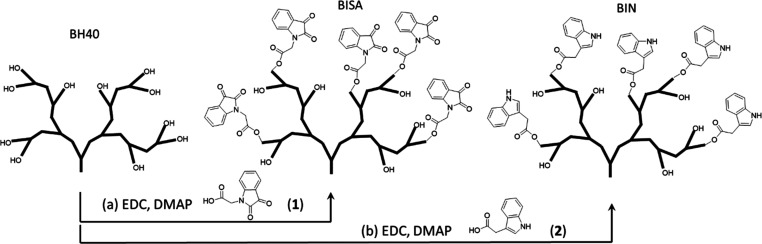
Synthesis of Isatin-Grafted HBP (BISA) and Indole-Grafted HBP (BIN) from Commercially Available HBP (BH40) Only schematic structures of the polymers are presented, and the number of OH groups and the grafted isatin and indole groups are not real.

Isatin- and indole-grafted HBPs (BISA and BIN) were synthesized by reacting carboxylic acid derivatives of isatin and indole (**1** and **2**) with BH40, a commercially available aliphatic polyester from Perstorp AB, Scheme 1. Indole-based grafting agent **2** is commercially available, and isatin-based grafting agent **1** was synthesized from isatin according to a modified 2-step synthetic protocol (Scheme S1, ESI).^67,68^ First, methyl isatin-*N*-acetate (**5**) was obtained by a convenient S_N_2 reaction between isatin (**3**) and a primary bromide (**4**) under mild basic condition. Hydrolysis of **5** gave **1** with ∼50% yield (over two steps). Transesterification of **5** with ethanol (the solvent) was a side reaction during this step, which yielded an ethyl ester (Figure S2, ESI) that could be completely removed by a straightforward precipitation (according to the ^1^H NMR results in Figure S3, ESI).

Grafting isatin or indole groups onto BH40 was achieved by a mild Steglich esterification using EDC and DMAP at room temperature.^69^ A slight excess (10 mol % excess with respect to the OH groups of BH40) of the grafting agents (**1** or **2**) was used. The corresponding polymers, BISA and BIN, were obtained in moderate yields after purification (42% and 45%, respectively). The grafting density, GD (i.e., the percentage of OH groups in BH40 that is consumed during the modification), of BISA and BIN was ∼78% and ∼81%, respectively, according to their ^1^H NMR spectra (Figures S4 and S5, Table S1, ESI). Further increasing the GD to ∼100% usually requires a large excess of grafting agents.^70^ In our case, synthesis of BISA and BIN with higher GD values has been attempted (Figures S6 and S7, ESI). As shown in Table S1 (ESI), the GD value of BISA remained almost unchanged (∼81%) using a 50% excess of **1** but was increased to 93% by the use of a 200% excess of **1**. For BIN, GD was increased to 94% by using a 50% excess of **2** but was not further increased even with a 200% excess of **2**. However, the isolated yields of BISA and BIN unavoidably decreased when a large amount of the grafting agents was used (Table S1, ESI), which could be attributed to the mass loss during purification.

It should be clarified that the presence of ∼20% unreacted OH groups (i.e., GD ≈ 80%) in BISA and BIN is desirable for our investigations because these hydrogen-bond donors are expected to enhance the interactions with the C=O groups in polyesters. In this article, we will focus only on the evaluation of the HBPs with ∼80% GD values.

### 3.2 Characterization of HBPs

The solubility of the obtained HBPs (BISA and BIN) and the precursor (BH40) was evaluated by examining whether 10 mg of polymer powder could be dissolved in a particular solvent (1 mL). As a result (Table 1), BISA is soluble in all of the tested aprotic solvents with a wide range of polarity. BIN also showed good solubility in aprotic solvents, except for the two least polar ones (chloroform and DCM). However, in protic solvents (ethanol, methanol, and water), both polymers are insoluble or only partially soluble. In general, the enhanced solubility of BISA and BIN in aprotic solvents was consistent with their decreased number of OH groups.

**Table 1:** Solubility of BH40, BISA, and BIN^a^

	solvent	BH40	BISA	BIN
aprotic solvents	chloroform	–	+	(⊕)
	DCM	–	+	(⊕)
	1,4-dioxane	(+)	+	+
	THF	+	+	+
	acetone	+	+	+
	acetonitrile	(⊕)	+	+
	DMF	+	+	+
	DMSO	+	+	+
	DMAc	+	+	+
protic solvents	ethanol	(⊕)	(⊕)	(⊕)
	methanol	(+)	(⊕)	(⊕)
	water	(+)	–	–

a Legend: + means soluble at room temperature, (+) means soluble after heating up to 60 °C, (⊕) means partially soluble after heating up to 60 °C, – means completely insoluble at 60 °C.

The molar masses of BISA and BIN were measured by GPC in chloroform. As shown in Table 2, the measured molar masses of BISA (*M*_n_ = 5740 g/mol) and BIN (*M*_n_ = 5002 g/mol) were higher than that of BH40 (*M*_n_ = 2833 g/mol, according to the supplier, Perstorp), which was consistent with grafting. The PDI values of BISA and BIN were lower than that of BH40, which could be attributed to the fractionation during purification by precipitation. This is consistent with the mass loss after grafting and purification (∼40% yields of BISA and BIN).

**Table 2:** Molecular Information and Thermal Properties of HBPs^a^

HBP	*M*_n_ (g mol^–1^)	*M*_w_ (g mol^–1^)	PDI	*T*_g_ (°C)	*T*_10_ (°C)	*T*_max_ (°C)	CY (%)
BH40	2833	5100	1.80	28	298	400	2.2
BISA	5740	7276	1.27	90	289	361	18.1
BIN	5002	6641	1.33	64	300	364	13.9

a *M*_n_, *M*_w_, and PDI were determined by GPC in chloroform. *T*_g_ (glass transition temperature) was measured from the DSC second heating curve; *T*_10_ and *T*_max_ are the temperature for 10% weight loss and the temperature for the maximum decomposition rate, respectively, according to the TGA data. Char yield (CY) was measured by TGA.

**Figure 1:**
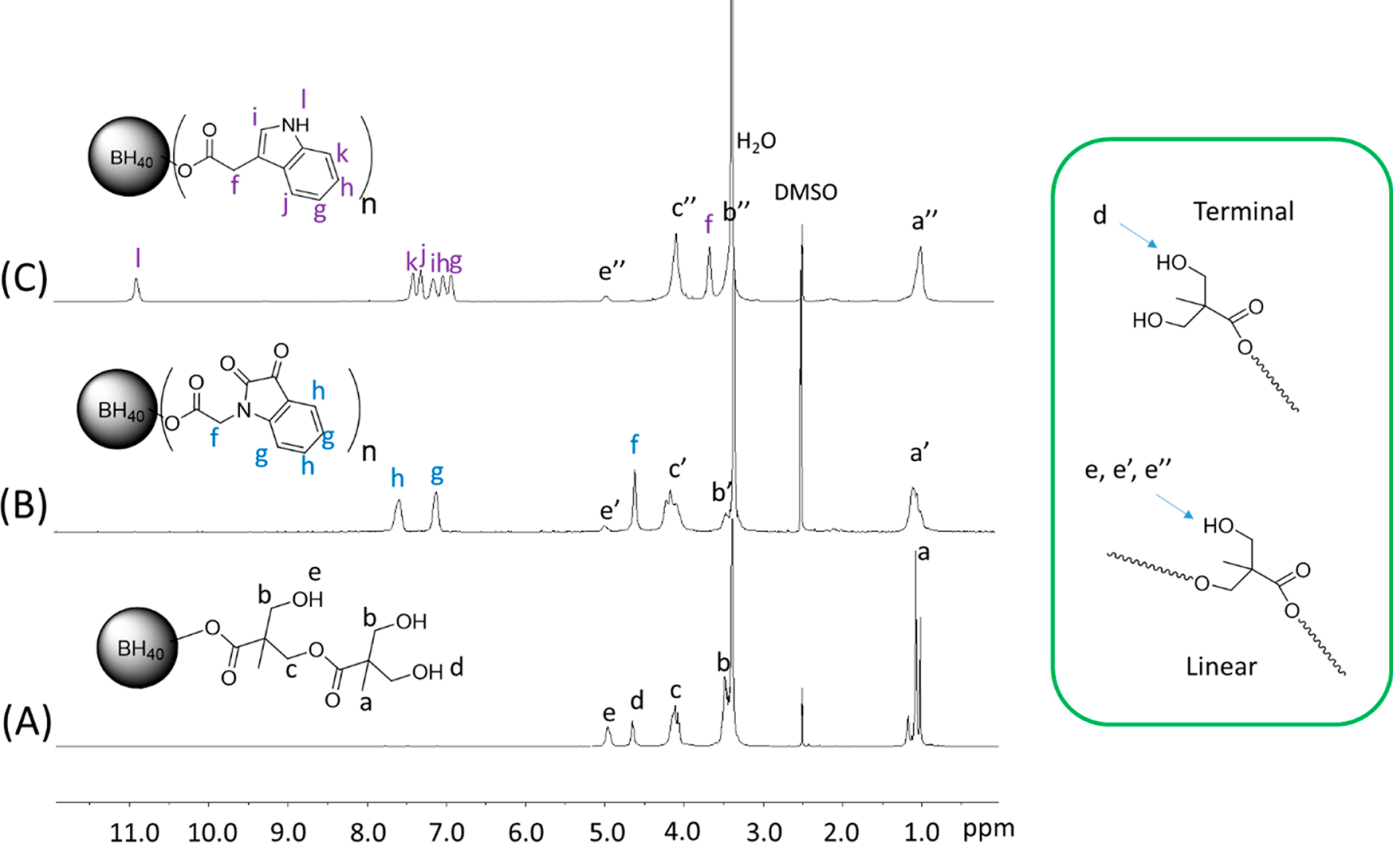
^1^H NMR spectra of BH40 (A), BISA (B), and BIN (C) in DMSO-*d*_6_. OH groups on the “terminal” or “linear” structural units are shown in the box on the right side.

The chemical structures of BISA and BIN were characterized by ^1^H NMR spectroscopy. In the ^1^H NMR spectrum of BH40 (Figure 1A), the signals corresponding to the methyl protons (*a*), the methylene protons (*b* and *c*), and the OH groups (*d* and *e* for terminal and linear units, respectively) were clearly discernible. After grafting (Figure 1B and 1C), the signal for the terminal OH groups disappeared while the signal for the linear OH groups remained (with significantly reduced intensity). This indicated that the terminal hydroxyl groups had higher reactivity for grafting and were completely consumed, but the linear hydroxyl groups were only partially grafted. All of the other characteristic signals for BH40 were observed in the spectra of BISA and BIN without significant changes in chemical shifts. In addition, two new aromatic signals of the isatin groups (*g* and *h*) and a new methylene signal at 4.61 ppm (*f*) were observed in the ^1^H NMR spectrum of BISA (Figure 1B). Similarly, five new aromatic signals at 7.42–6.94 ppm (*g*–*k*) and a new signal at 3.68 ppm (methylene proton close to the indole ring, *f*) were observed in the spectrum of BIN (Figure 1C). These new signals further confirmed the success of grafting. A comparison of the integrals of the OH signal, the aromatic signals, the indole N–H signal, and the backbone methyl signal was used to estimate the GD values (∼78% and ∼81% for BISA and BIN, respectively, Figures S4 and S5, ESI), as discussed earlier.

**Figure 2:**
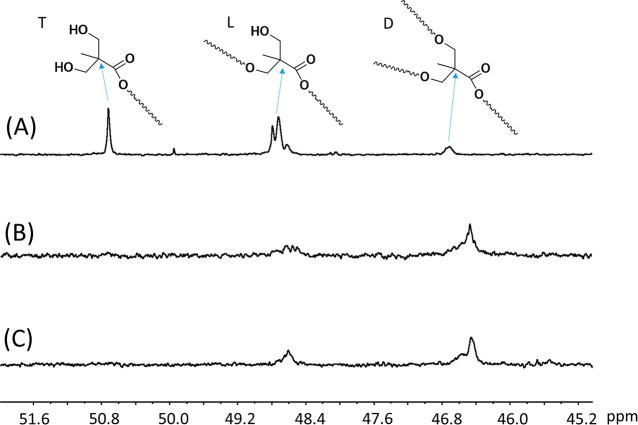
^13^C NMR spectra of BH40 (A), BISA (B), and BIN (C) with the expansion in the range of 45–52 ppm. Quaternary carbons for the terminal (T), linear (L), and dendritic (D) structural units are designated by the arrows.

The chemical structures of BISA and BIN were further characterized by ^13^C NMR spectroscopy (Figure 2). As shown in the expanded region of the quaternary carbons (45–52 ppm), discernible signals corresponding to the quaternary carbons of the terminal (T), linear (L), and dendritic (D) structural units of BH40 were observed at 50.7, 48.7, and 46.6 ppm, respectively, consistent with the literature (Figure 2A).^71^ The signal of the terminal (T) quaternary carbon completely disappeared in the spectra of BISA (Figure 2B) and BIN (Figure 2C), which confirmed the consumption of the terminal OH groups. In the meantime, the relative intensity of the signals of the linear quaternary carbons (L) with respect to that of the signals of dendritic carbons (D) significantly decreased after grafting (Figure 2 B and 2C compared with Figure 2A). This indicated that the hydroxyl groups on the linear units of BH40 partially reacted, which was consistent with the ^1^H NMR results discussed earlier.

**Figure 3:**
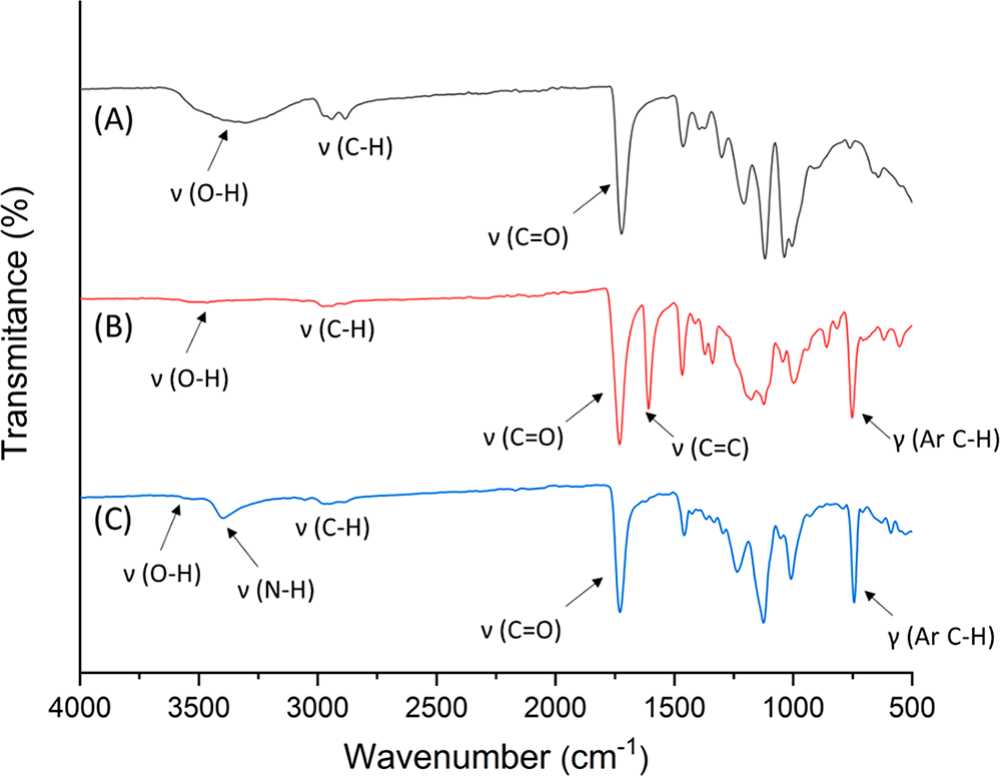
FT-IR spectra of BH40 (A), BISA (B), and BIN (C) with characteristic bands assigned.

The chemical structures of BISA and BIN were further confirmed by FT-IR spectra (Figure 3). The broad O–H stretching band (centered at ∼3346 cm^–1^) of BH40 (Figure 3A) was almost invisible in the spectra of BISA (Figure 3B) and BIN (Figure 3C). In addition, a new band at ∼3395 cm^–1^ was observed in the FTIR spectrum of BIN (Figure 3C), which was attributed to the N–H stretching of indole. The strong characteristic ester C=O stretching band was observed in the spectra of all HBPs (1721, 1732, and 1729 cm^–1^ for BH40, BISA and BIN, respectively). In addition, a strong band at 1608 cm^–1^ in the spectrum of BISA (Figure 3B) corresponded to the aromatic C=C stretching, which was consistent with the FT-IR spectrum of the isatin-based grafting agent **1** (Figure S9, ESI). This band was not observed in the FT-IR spectra of BIN and grafting agent **2** (Figure 3C and Figure S9, ESI). Finally, the out-of-plane bending vibrations of aromatic C–H for BISA (753 cm^–1^) and BIN (746 cm^–1^) were observed, which confirmed the presence of isatin and indole groups in the polymers.^72^

**Figure 4:**
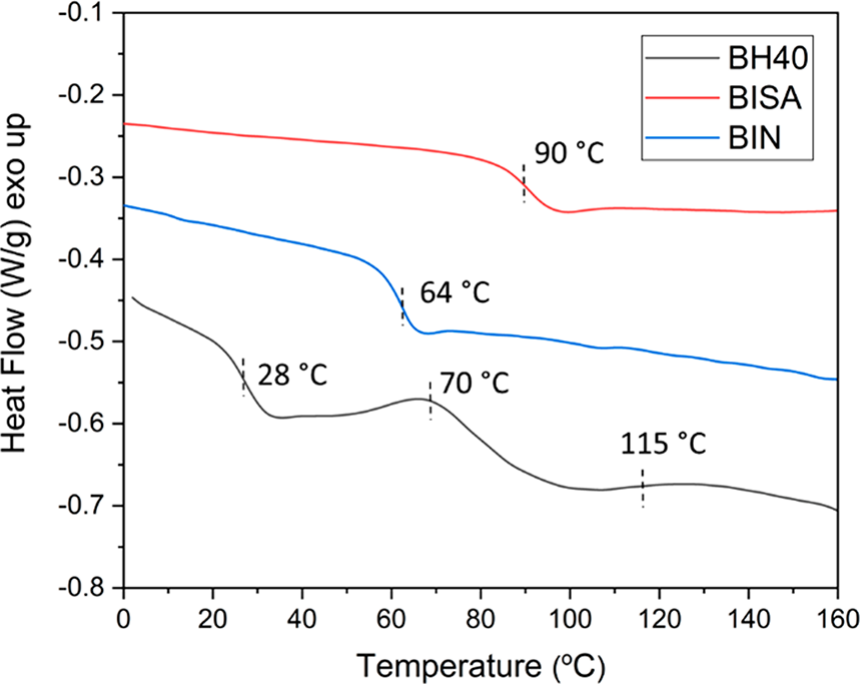
DSC second heating curves of BH40, BISA, and BIN.

**Figure 5:**
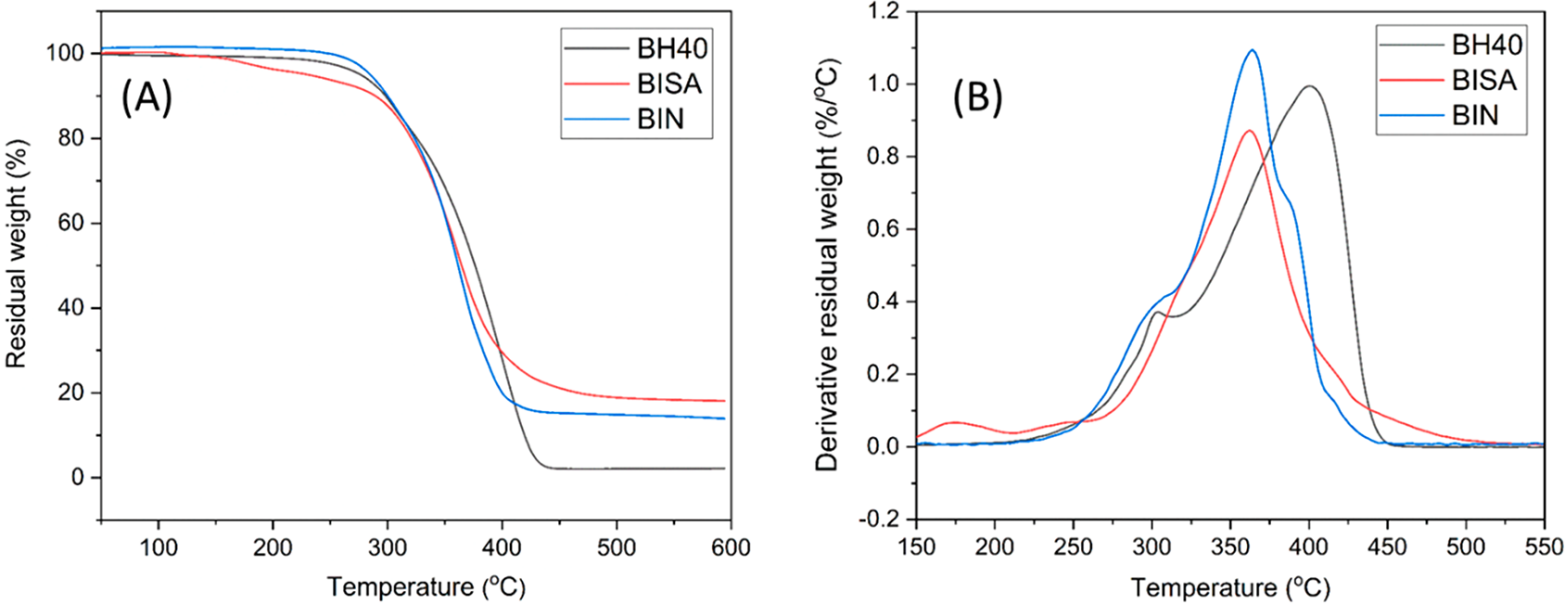
TGA residual weight (A) and derivative weight loss (B) curves of BH40, BISA, and BIN.

The thermal properties of BISA and BIN were characterized by DSC and TGA analyses. According to the DSC results (Figure 4), the glass transition temperatures (*T*_g_) of BISA and BIN are 90 and 64 °C, respectively, which are significantly higher than that of BH40 (*T*_g_ ≈ 28 °C). This can be attributed to the incorporation of cyclic aryl groups (indole or isatin), which can increase the overall structural rigidity (and *T*_g_) of linear and dendritic polymers.^59,73,74^ Furthermore, BISA showed a higher *T*_g_ than BIN, which could be due to the stronger dipole interactions of BISA (dipole moment of ∼5.65 D for isatin and ∼2.07–2.38 D for indole groups).^75,76^ Furthermore, the second heating curve of BH40 displayed a broad exotherm at ∼70 °C and a broad endotherm at ∼115 °C, which could be attributed to the formation and cleavage of hydrogen bonds by the OH groups in BH40.^77^ These exo- and endothermic events were not observed for BISA and BIN, which indicated that the remaining OH groups (∼20%) did not form significant hydrogen bonds. According to the TGA results (Figure 5 and Table 2), the decomposition onset temperature *T*_10_ (the temperature for 10% mass loss) was similar for BISA, BIN, and BH40. However, the temperatures for the maximal rate of weight loss (*T*_max_) for BISA and BIN were ∼30–40 °C lower compared with that of BH40, which could be attributed to thermal decomposition of the isatin and indole groups (confirmed by the thermal decomposition patterns of grafting agents **1** and **2**, Figure S10, ESI). Finally, BISA and BIN also showed higher residual char yields (CY) than BH40, which could be attributed to their aromatic units.^26,78^

### 3.3 Leakage Assessment of BISA and BIN from PHB Films

**Figure 6:**
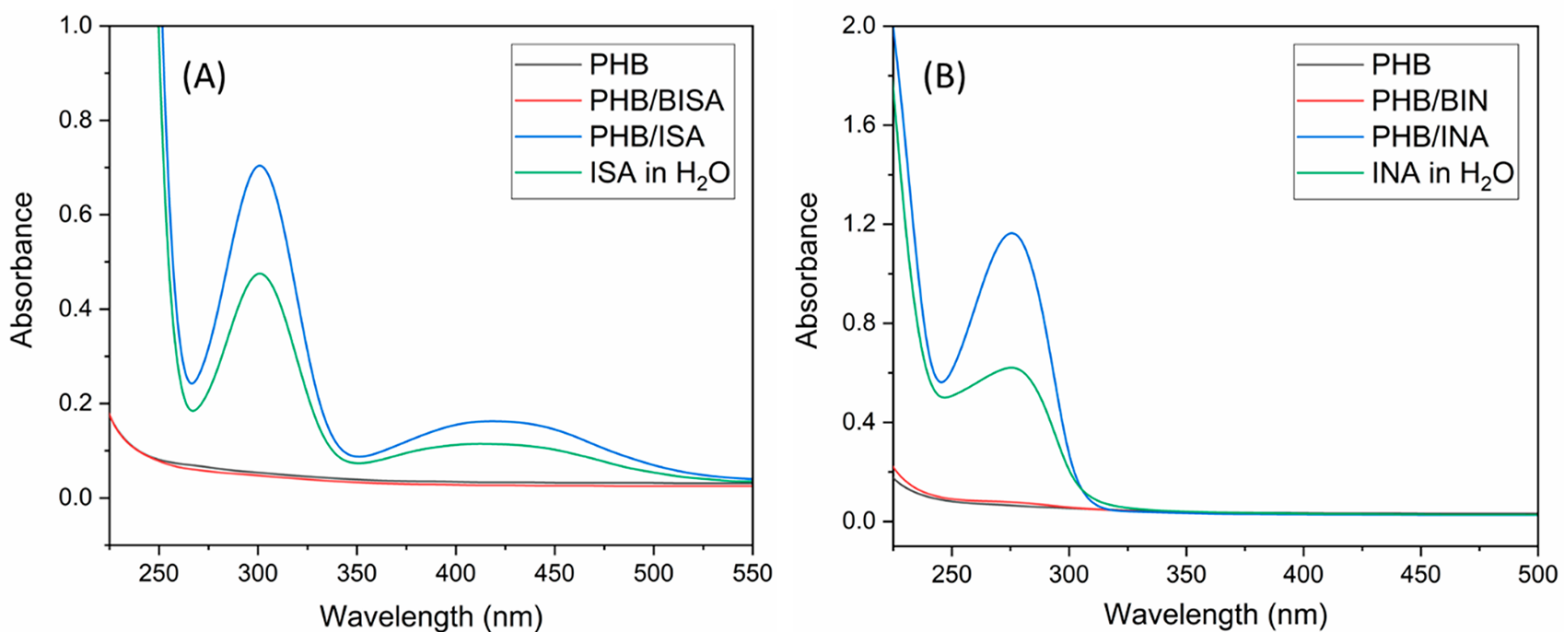
UV–vis absorbance spectra of the aqueous phase after pure PHB films or PHB films with (A) BISA or isatin (ISA) and (B) BIN or indole-3-acetic acid (INA) were immersed in deionized water for 5 days. For comparison, UV–vis spectra of ISA and INA in water are also shown in A and B, respectively.

The short-time leaching potential of BISA and BIN from biodegradable plastic PHB films into water was evaluated by measuring the UV–vis spectra of the aqueous phase after PHB films with BISA or BIN (5%) were immersed for 5 days. As shown in Figure 6, the UV–vis absorbance of the aqueous phase was negligible after PHB film with or without HBP additives were immersed. On the contrary, a significant UV–vis absorbance was observed in the aqueous phase after the PHB films containing small molecular agents (ISA or INA) were immersed for 5 days. This revealed the low short-time leaching potential of BISA and BIN from the PHB matrix into the aqueous environment, which was consistent with our previously reported isatin-based nonionic HBPs.^26^ The low leaching potential of BISA or BIN was also confirmed by the low coloration of the aqueous phases after the PHB films containing BISA and BIN were immersed (Figure S11, ESI), while the aqueous phase for immersing the films containing small molecules (ISA or INA) was colored. It should be clarified that since a zone of inhibition was observed in the disk diffusion antimicrobial assay (see later discussions), it is expected that a small quantity of the HBPs should be able to migrate out from the cellulose paper matrix in that case (different from the PHB matrix in the leaching tests here), but the quantity is expected to be low.

### 3.4 Miscibility of HBP with Biodegradable Polyesters

**Figure 7:**
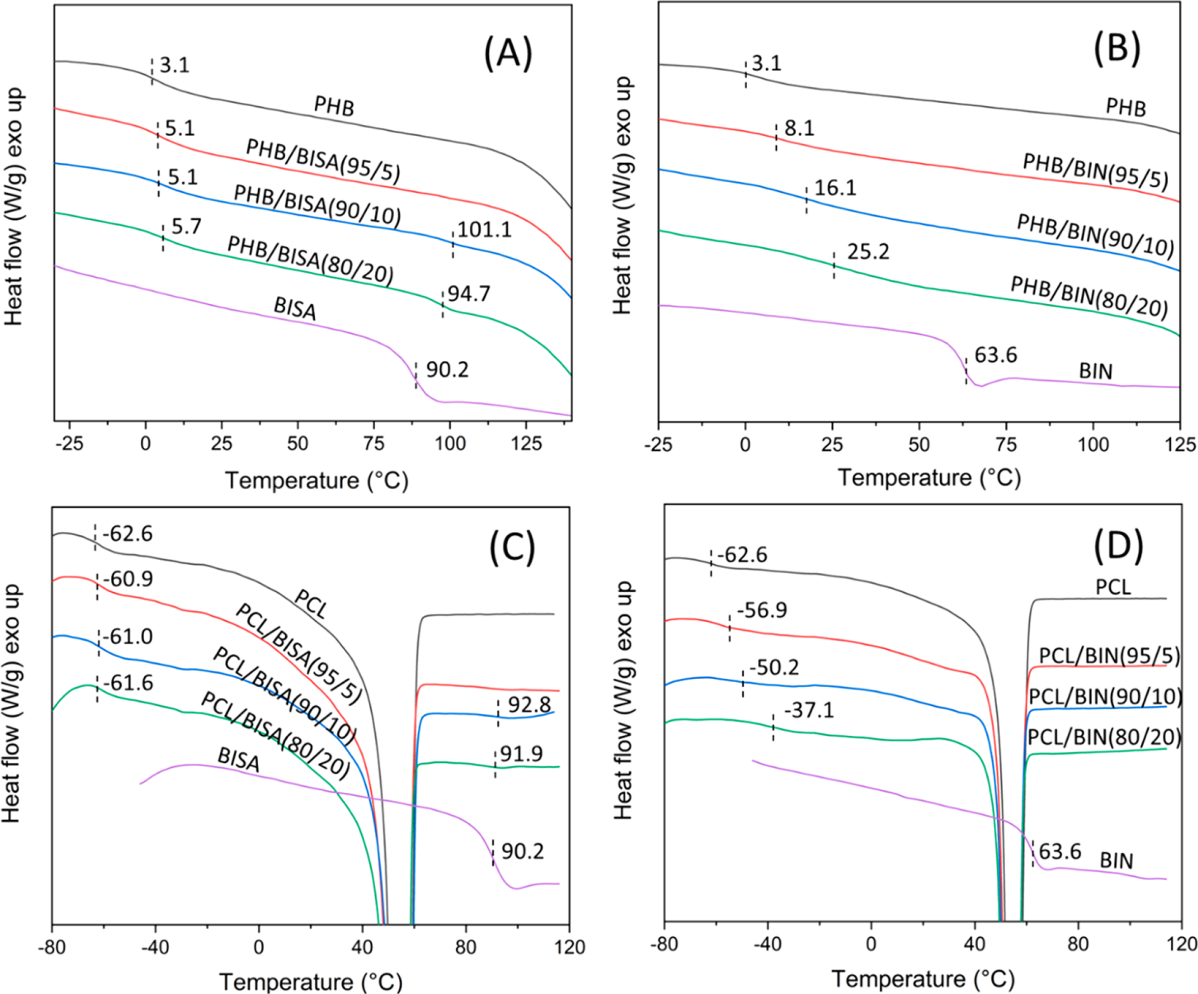
DSC second heating curves of (A) neat PHB and PHB with 5, 10, and 20 wt % of BISA and neat BISA, (B) neat PHB with 5, 10, and 20 wt % of BIN and neat BIN, (C) neat PCL and PCL with 5, 10, and 20 wt % of BISA, and (D) neat PCL with 5, 10, and 20 wt % of BIN.

The miscibility of up to 20 wt % HBPs (BISA and BIN) with two different biodegradable and biomedically relevant polyesters (PHB and PCL)^79,80^ was evaluated by DSC. For PHB/BISA blends (Figure 7A), the two *T*_g_ values corresponding to PHB- and BISA-rich phases were clearly observed for the 80/20 and 90/10 blends, which indicated that they were immiscible. For 95/5 blend, only one *T*_g_ value was observed (∼5.1 °C), but it was very close to the *T*_g_ of the PHB-rich phase of 80/20 and 90/10 blends. This suggested that the blend was immiscible, but the *T*_g_ of the BISA-rich phase was not observed due to the low BISA content in the blend. For PHB/BIN blends (Figure 7B), only one *T*_g_ was observed with all three compositions (5, 10, and 20 wt %), and the *T*_g_ value increased as the increased BIN content. This indicated that PHB/BIN blends with up to 20 wt % BIN were miscible.

A similar observation was made in the case of PCL-based blends (Figure 7C and 7D). All of the PCL/BISA blends showed similar *T*_g_ values as pure PCL (Figure 7C). For 80/20 and 90/10 blends, the *T*_g_ for the BISA-rich phase was visible, while the *T*_g_ for the 95/5 blend was not observed (due to the low content of BISA). These observations indicated that BISA was immiscible with PCL in the measured composition range. However, all PCL/BIN blends exhibited only a single *T*_g_ over the entire blend compositions, and their *T*_g_ values shifted to higher temperatures with increasing BIN contents (Figure 7D). This indicated that these blends were miscible with up to 20 wt % BIN.

DSC data also provided valuable information regarding the crystallization behavior of the miscible blends of BIN with biodegradable polyester matrices (Table 3). Both PHB and PCL matrices showed similar results. The melting endotherm (in the second cycle heating curves, Figure S12A and S12C, ESI) and crystallization exotherms (in the first cycle cooling DSC curves, Figure S12B and D, ESI) of the blends became broader and shifted toward lower temperature as the BIN content increased in the blends, which indicated that the presence of BIN retarded the crystallization of PHB or PCL. This observation could be attributed to the favorable intermolecular hydrogen-bond interactions between BIN and the carbonyl groups of the polyester matrix (Figure S13, ESI), which is consistent with other reported miscible blends with hydrogen bonds (e.g., PHB blends with cellulose acetate butyrate or chitosan).^81,82^

**Table 3:** Thermal Properties of PHB/BIN and PCL/BIN Blends According to DSC Measurements^a^

samples	*T*_g_ (°C)	*T*_m_ (°C)	*T*_c_ (°C)	Δ*H*_f_ (J/g)	*χ*_c_ (%)
PHB	3.1	174.8	120.2	99.6	68.2
PHB/BIN (95/5)	8.1	167.8	117.0	97.0	69.9
PHB/BIN (90/10)	16.1	162.6	114.8	93.0	70.8
PHB/BIN (80/20)	25.2	158.8/148.8	107.6	73.5	62.9
PCL	–62.6	54.5/57.0	26.2	74.6	54.0
PCL/BIN (95/5)	–56.9	53.9/56.5	26.1	74.8	53.6
PCL/BIN (90/10)	–50.2	53.3/56.4	26.0	76.6	54.8
PCL/BIN (80/20)	–37.1	53.2/56.0	22.4	79.6	55.7

a *T*_g_ (glass transition temperature) and *T*_m_ (melting temperature) were measured from the second DSC heating curve; *T*_c_ (crystallization temperature) was measured from the first DSC cooling curve. Δ*H*_f_ (enthalpy of crystallization) and χ_c_ (degree of crystallinity) were measured by DSC.

In addition, the impact of blending on the crystallinity was also revealed by DSC results. The degree of crystallinity (χ_c_) of PHB and PCL blends with BIN was calculated using the areas of the melting endotherms (Figure S12, ESI), taking the Δ*H*_f_ values of 100% crystalline PHB and PCL as 146 and 139 J/g, respectively.^83−85^ For PHB/BIN blends, the crystallinity (χ_c_ values) increased slightly upon blending with 5–10% BIN (Table 3), which could be due to the plasticizing effect of BIN in the blend that increased the chain mobility of PHB.^86−88^ A similar observation has been reported for PHB blends with 5–7% Lapol (a commercial polyester plasticizer).^89^ When 20 wt % of BIN was blended with PHB matrix, two melting endotherms were observed (Figure S12A), indicating two different crystalline structures in the blend. The χ_c_ value decreased significantly, which indicated that the plasticizing effect of BIN was counteracted by its disruption in the crystallization of PHB, as for other reported PHB blends.^74,90^ For PCL/BIN blends, the crystallinity (χ_c_) and melting enthalpy were insignificantly influenced by blending, which could be attributed to the opposite plasticizing and nucleating effects^91−93^ of the blended BIN that canceled each other to a large extent.

The miscibility of BIN-based blends and immiscibility of BISA-based blends can be attributed to their molecular structures, which can or cannot induce hydrogen-bonding interactions.^94^ BIN contains indole N–H as a hydrogen donor that can form hydrogen bonds with the oxygen atoms in PHB and PCL (PHB/BIN as an example in Figure S13, ESI). On the contrary, BISA contains no active hydrogen donor, so it has relatively poor interactions with PHB and PCL. To further confirm this explanation, a methyl-modified indole-based HBP (namely, *m*-BIN) without active H donor was synthesized for comparison (Figure S14 and Scheme S2, ESI). In order to exclude the influence from the remaining OH groups, the GD value of *m*-BIN was carefully controlled (∼82%) to be consistent with that of BIN (∼80%). As a result (Figure S15A, ESI), two *T*_g_ values were clearly discernible for PHB/*m*-BIN blends with 90/10 and 80/20 compositions, which indicated that they were immiscible. For PHB/*m*-BIN-95/5 blend, only one *T*_g_ was observed at 7.1 °C, which was close to the *T*_g_ values for 90/10 and 80/20 blends. This observation was consistent with the PHB/BISA blends discussed earlier (Figure 7A) and was significantly different from the PHB/BIN miscible blends (Figure 7B). Similarly, PCL/*m-*BIN blends (Figure S15B, ESI) showed similar behavior as PCL/BISA blends but different from PCL/BIN blends. These observations confirmed that the observed miscibility between BIN and biodegradable aliphatic polyesters could be attributed to the presence of indole N–H as hydrogen-bond donors.^95^

### 3.5 Antibacterial Activity

**Figure 8:**
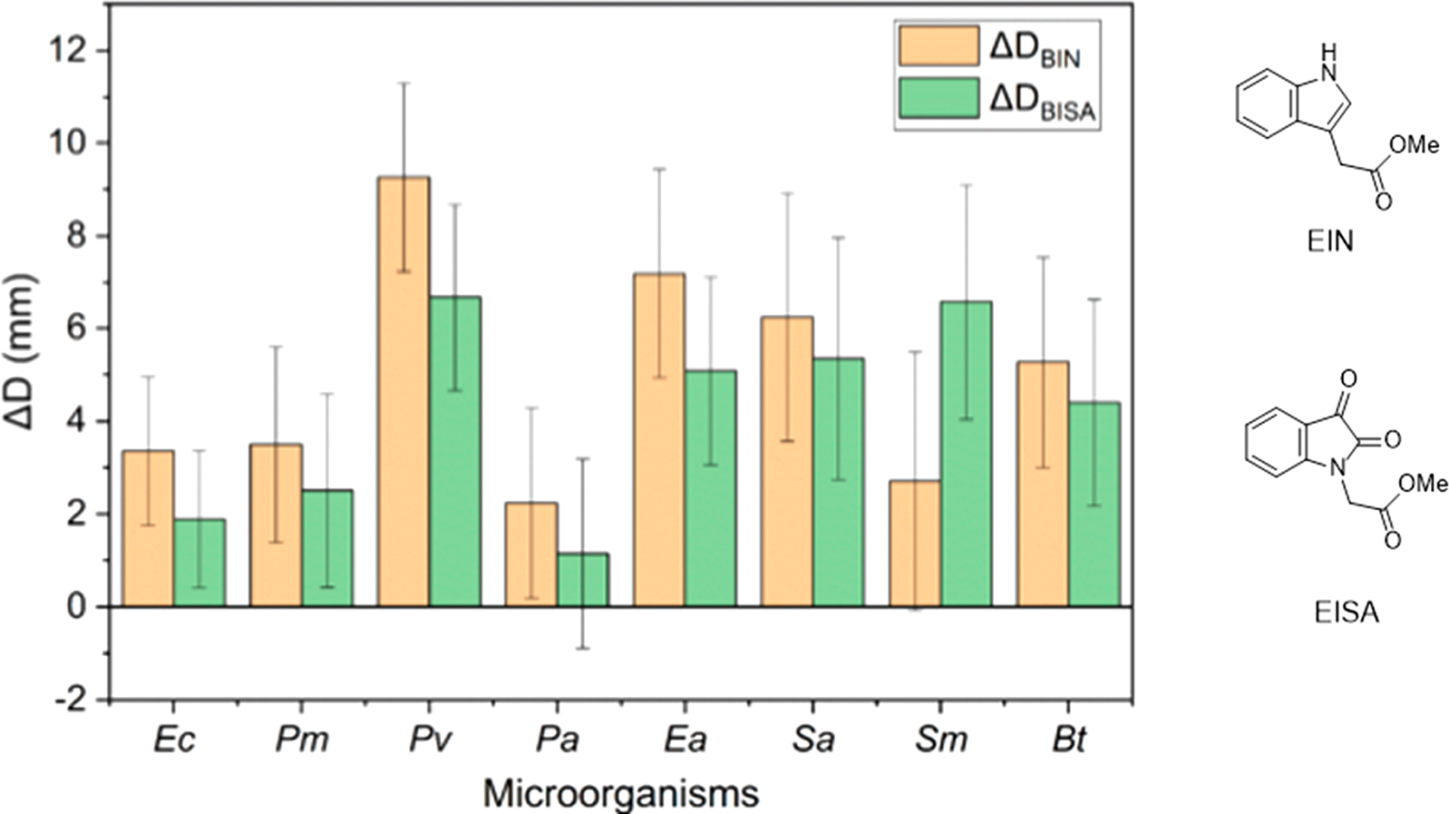
Comparison of the zones of inhibition of polymeric antimicrobials with the corresponding small molecular agents with the same functional groups. Δ*D*_BISA_ = *D*_BISA_ – *D*_EISA_, Δ*D*_BIN_ = *D*_BIN_ – *D*_EIN_. *D*_BISA_, *D*_EISA_, *D*_BIN_, and *D*_EIN_ are the diameters of the inhibition zones of BISA, EISA, BIN, and EIN, respectively.

**Figure 9:**
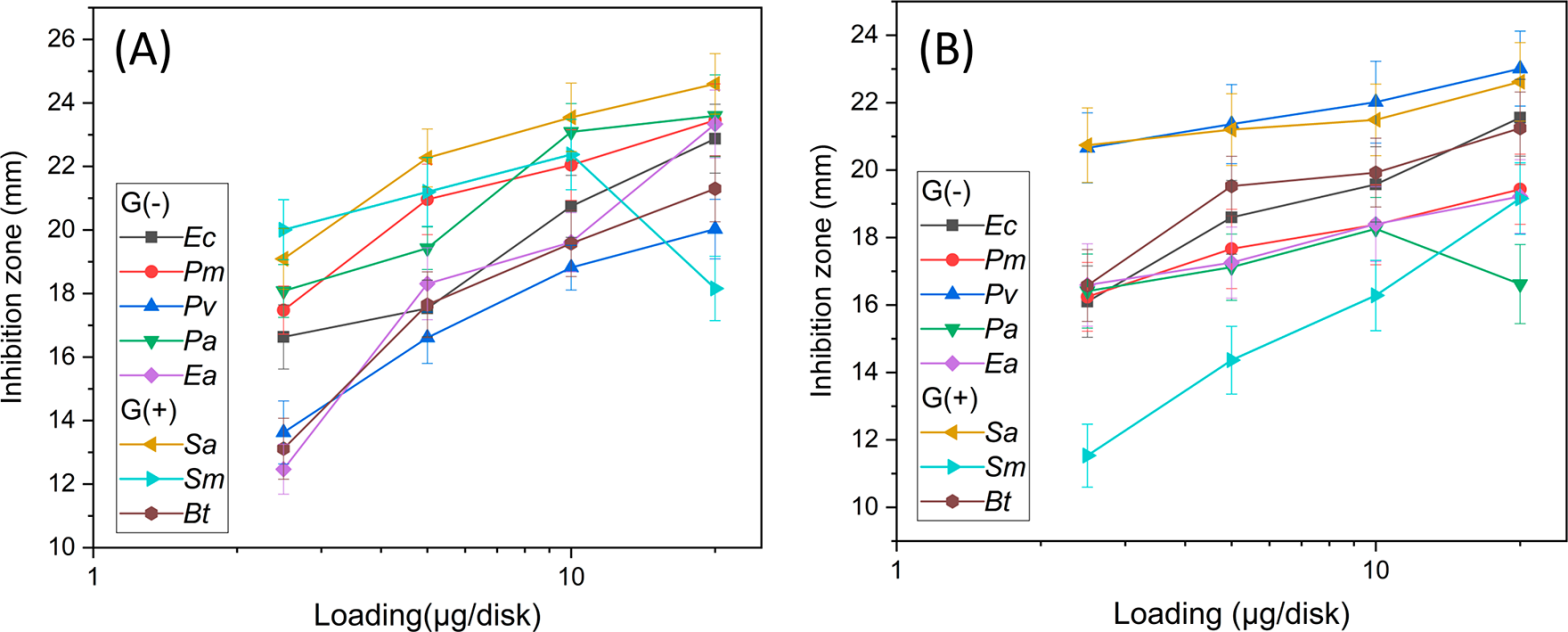
Zone of inhibition of (A) BISA and (B) BIN as a function of the loading amount (micrograms per disk). For clarity, the error range of the data points was omitted, which can be found in Figure S18.

Disk diffusion assay was carried out to evaluate the antibacterial activity of HBPs and two small molecules (EISA and EIN) against eight human pathogens, including five Gram-negative (*Ec*, *Pm*, *Pv*, *Pa*, and *Ea*) and three Gram-positive bacteria (*Sa*, *Sm,* and *Bt*). As shown in Figure S17, both BISA and BIN with 10 μg per disk loading showed a significant zone of inhibition (∼14–20 mm) against all of the tested bacteria (example images shown in Figure S16, ESI), and there was no significant difference between their effects (*p* > 0.05, entry 1, Table S2, ESI). The difference in the zone of inhibition between the polymers (BISA and EISA) and their corresponding small molecules (EISA and EIN) is presented in Figure 8 (full data set shown in Figure S17, ESI). Compared to the corresponding small molecules with isatin and indole functions (EISA and EIN) with the same sample loading (10 μg per disk), polymeric BISA and BIN showed a larger zone of inhibition against all of the tested bacteria (significantly different for most bacteria except for *Pa* for BISA/EISA and *Pa*, *Sm* for BIN/EIN, entries 2 and 3, Table S2, ESI), which could be attributed to the intensified interactions between the locally concentrated functional groups (isatin or indole) and bacteria, as another reported isatin-based HBP with more rigid structure.^26^ Moreover, ΔBIN were generally greater than ΔBISA except for *Sm*, which suggested that hyperbranched architectures were more effective for indole-based structures. In addition, compared to gentamicin (a commercial antibiotic), BISA and BIN were significantly more effective for three tested bacteria (*Ec, Sa, Sm*), comparable for two bacteria (*Pm* and *Bt*), and significantly less effective for two Gram-negative bacteria (*Pa* and *Ea*). For bactrium *Pv*, BIN was significantly more effective than gentamicin and BISA was comparable to gentamicin (confirmed with *p* values, entries 4 and 5, Table S2, ESI). Finally, the effect of the sample loading was investigated (Figure 9 and Figure S18, ESI). In general, both HBPs (BISA and BIN) showed higher antibacterial activity with higher sample loading in the range of 2.5–20 μg per disk with only two exceptions. For *Sm* (G+) with BISA and *Pa* (G-) with BIN, the largest zone of inhibition was observed at 10 μg per disk (Figure 9).

It should be noted that BISA and BIN (and their corresponding small molecular agents EISA and EIN) all contain ester groups, which may also interact with bacteria.^2^ To gain insight into the structure–function relationship, we measured the antibacterial properties of four small functional molecules with (EISA and EIN) or without (isatin and indole) ester bonds (with ester groups). For all of the tested bacteria except for *Ec* for EISA/ISA (Figures S19 and S20, ESI), isatin (ISA) and indole (IN) did not show a significantly different antibacterial effect compared to EISA and EIN, respectively (*p* > 0.05, entries 6 and 7, Table S2, ESI). This suggests that the ester groups did not play a major role in the antimicrobial function in our cases, and the observed antibacterial effect of EISA and EIN was mainly due to the effects of isatin or indole groups. For macromolecular agents BISA and BIN, further investigations will be needed to understand their similarity/difference with small molecular agents in terms of the antibacterial mechanism.

### 3.6 Anti-Quorum Sensing (Anti-QS)

Quorum sensing (QS) is an important biological process for bacteria, which is usually mediated by certain small signal molecules such as acylated homoserine lactones (AHLs). This process regulates the production of important compounds such as virulence molecules for biofilm formation. QS is considered to have a close relationship with biofilm formation and development of antibacterial resistance. Therefore, inhibition of QS may have promising potential to prevent or retard these processes.^96−99^ In this study, we determined the disruption activity of HBPs (BISA and BIN) and small molecules (EISA and EIN) for AHL-regulated QS response against *CV026*. The anti-QS property was measured by the zone of a halo formation, which illustrates the inhibition of purple violacein production during bacterial growth around the disc. According to the results (Figure S21, ESI), the HBPs and small molecules inhibited the quorum sensing of *CV026*, which was regulated by the signal molecule *N*-hexanoyl-l-homoserine lactone, HSL. The disc with pure solvent (negative control) showed no significant effect on pigment inhibition, and the disc containing gentamicin (positive control) showed a smaller inhibition zone (12.3 ± 0.6 mm) than HBPs (BIN and BISA) and small molecule EIN at the same loading amount (10 μg/mL). The measured HBPs (BISA and BIN) exhibited larger inhibition zones of pigment compared with their corresponding small molecules (i.e., 15.6 ± 1.9, 13.9 ± 0.8, 13.8 ± 0.4, and 12.0 ± 0.5 mm for BIN, BISA, EIN, and EISA, respectively). According to our results, the isatin- and indole-based nonionic HBPs and the small molecules all exhibited a significant anti-QS effect, so they may have potential to act as antibiofilm agents.

### 3.7 Enzymatic Degradation

The backbones of BISA and BIN are hyperbranched aliphatic polyester (BH40) structures, which are in principle biodegradable.^54^ However, it is unknown how the grafted isatin or indole units will affect the biodegradation. In this work, enzymatic degradation of BISA and BIN using PETase was investigated according to a previously reported method.^59,60^ The powders of BISA and BIN were treated with PETase from *Ideonella sakaiensis*(55) for 3 days. Reaction with a longer time was not carried out due to the reduced enzyme activity. The aqueous phase was analyzed by LC-MS. Negative control (without enzyme) was carried out for each degradation experiment.

**Figure 10:**
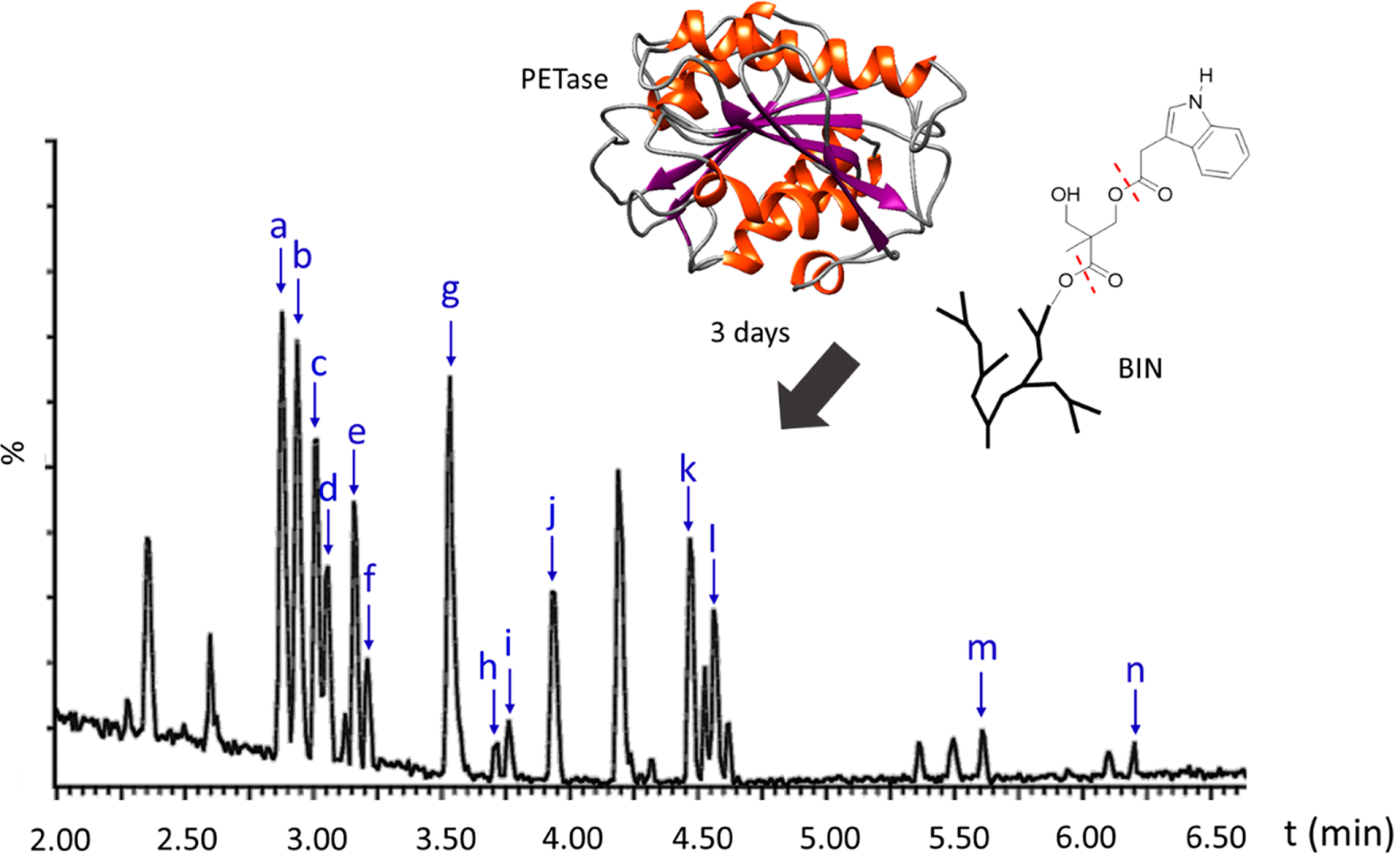
Base peak chromatograms (LC-MS) of the aqueous phase after BIN was reacted with PETase for 3 days. All 14 new signals (not present in the negative control, see Figure S22, ESI) are marked as a–n in the spectrum. Corresponding MS spectra of a–n are presented in Figure S23, ESI. The unassigned peaks are all present in the negative control (Figure S22, ESI).

For BIN, the ion chromatograms displayed 14 additional signals (marked as a–n, Figure 10) after the reaction with PETase, which were absent in the negative control experiment (Figure S22, ESI). These new signals are clearly due to the reaction with PETase, and thus, they were carefully examined by mass spectrometry. In the corresponding mass spectra of the 14 new ion signals (Figure S23, ESI), various monomer and oligomer structures could be identified (Figure S24, ESI), which indicated that BIN was degraded by PETase under the experimental conditions. For BISA, the ion chromatograms showed only one new signal (at 3.56 min, absent in negative control) after the reaction with PETase (Figure S25A and S25B, ESI). However, the corresponding mass spectrum (Figure S25C, ESI) only displayed a group of signals in the range of 600–1200 *m*/*z* with increasing intervals, which was due to multiple-charged species related to enzyme denaturing in the presence of DMSO,^100^ but not enzymatic degradation of BISA. For comparison, the nongrafted BH40 was also subjected to the same degradation conditions, which showed no significant difference with the negative control in the ion chromatograms (Figure S26, ESI). This preliminary result suggested that the grafted isatin groups may have a negative impact on the enzymatic degradation of hyperbranched polyester structures, while the grafted indole units could facilitate such biodegradation.

**Figure 11:**
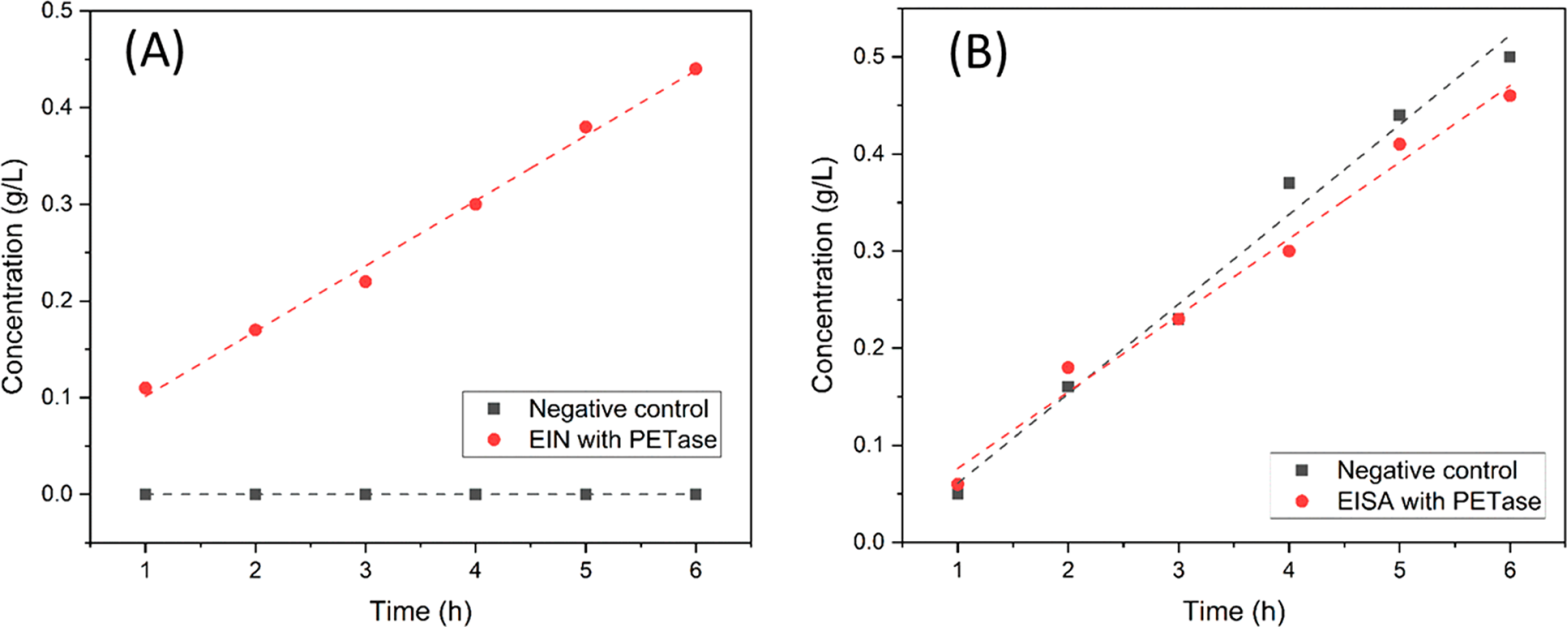
PETase reaction rates. Formation of pro-EIN (A) and pro-EISA (B). Concentration values were calculated based on the integrals of the HPLC peaks.

To further understand the impact of isatin or indole on the enzymatic degradation of ester bonds, the two corresponding monomers (EISA and EIN, structures see Figure 8) were subjected to the same enzymatic degradation with PETase. The initial hydrolysis kinetics were investigated by HPLC analysis of the supernatant after calibration of the HPLC peak area and analyte concentration using standard solutions of isatin-*N*-acetic acid and indole-3-acetic acid (Figure S27, ESI). During the enzymatic reactions, EISA and EIN were consumed and the intensity of the hydrolysis products (namely, pro-EISA and pro-EIN at retention times *t* ≈ 0.69 and 1.80 min, respectively) was growing during the reaction time, as indicated by the chromatograms of the reactions (Figure S28–S31). The kinetics of pro-EIN and pro-EISA formation with and without PETase are plotted in Figure 11. For EIN (Figure 11A), the formation of pro-EIN clearly followed pseudo-first-order kinetics (*k* ≈ 6.74 × 10^–2^ g L^–1^ h^–1^), which is consistent with other reported ester hydrolyses with PETase or other enzymes.^101,102^ The negative control showed insignificant hydrolysis, which confirmed the effect of PETase. This result revealed that the hydrolysis of ester bonds could be facilitated by the presence of the indole functionality, which corroborated the results of enzymatic degradation of polymer BIN (discussed earlier). On the contrary, the presence of isatin showed a complex impact on the enzymatic degradation (Figure 11B). Pseudo-first-order kinetics were observed regardless of the presence of PETase, and the rate constant for negative control was slightly higher (*k* ≈ 9.23 × 10^–2^ g L^–1^ h^–1^) than that of the reaction with PETase (*k* ≈ 7.89 × 10^–2^ g L^–1^ h^–1^). This indicates that the presence of isatin groups may inhibit the function of PETase but can promote the hydrolysis of ester bonds through a nonenzymatic mechanism. This observation was generally in agreement with the reported inhibition of similar enzymes, carboxylesterases, by isatin.^103^ The degradation results from monomeric model compounds are in general consistent with the observations for polymer biodegradation, although in the latter case the steric effect of the densely grafted functional groups in polymers may also have an impact on the enzymatic degradation process.

### 3.8 Molecular Docking

**Figure 12:**
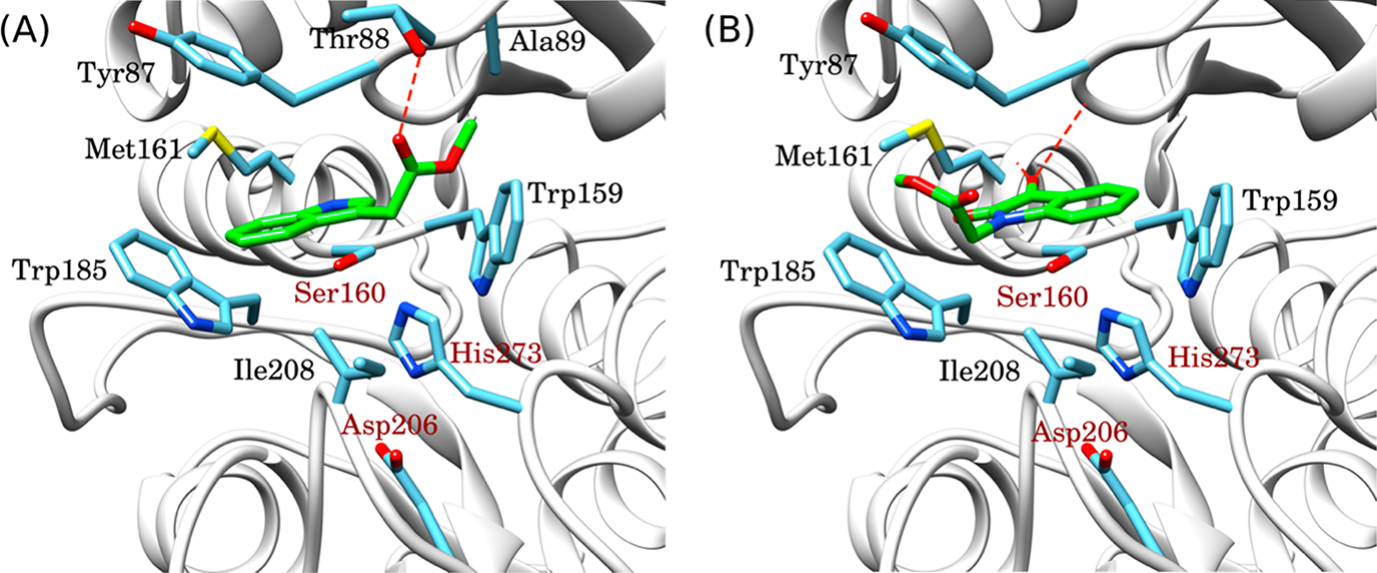
Molecular docking of monomers into the PETase active site: (A) EIN and (B) EISA. Catalytic amino acids are labeled in dark red. Predicted hydrogen bonds are represented by red dashed lines.

Molecular docking simulation of monomeric molecules (EIN and EISA) provided more insights into the impacts of indole or isatin groups on the PETase-catalyzed degradation of ester bonds. The docking simulation has shown that EIN and EISA both bind into the active site of PETase but in different orientations (Figure 12). The indole ring of EIN interacts with Trp185 (Figure 12A), while in EISA, the isatin ring oriented oppositely, interacting with Trp159 (Figure 12B). The carbonyl at position 3 of the isatin ring in EISA forms two hydrogen bonds (=O···H–N) with the PETase backbone at positions Tyr87 and Met161. These interactions could contribute to stronger binding of EISA than EIN with a difference of 1.1 kcal/mol. Thus, the carbonyl group could also be determinant for the ligand orientation into the active site. EIN bonded in an opposite orientation, with a hydrogen bond between the carbonyl of the ester bond and the hydroxyl group of Thr88 (red dashed line in Figure 12A). Despite the relatively weaker binding of EIN into PETase (compared to EISA), the ester bond is oriented closer to the catalytic amino acids (Ser16 and His273), which could facilitate its enzymatic degradation.

### 3.9 Cytotoxicity

**Figure 13:**
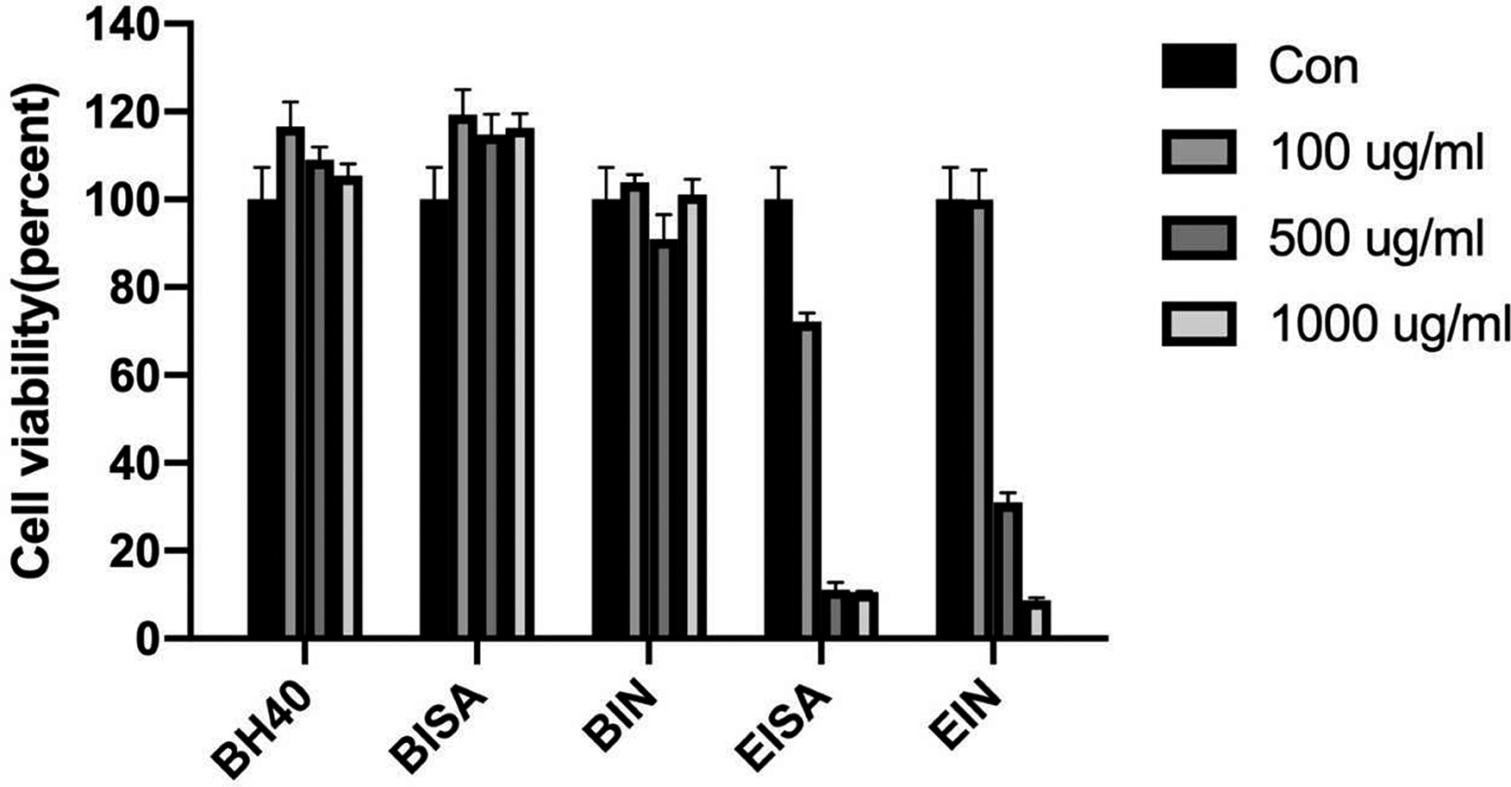
Cytotoxicity of BH40, BISA, BIN, EISA, and EIN at three concentrations (100, 500, and 1000 μg/mL). Results are presented as percent viability of treated cells compared to that of untreated control (shown as Con in the graph).

The cytotoxicities of three HBPs (BH40, BISA, and BIN) and two small molecules (EISA and EIN) were assessed against MG-63 osteoblast-like cells using MTT assay. The cell viability results are summarized in Figure 13. For the three tested HBPs, the results demonstrated that the cell toxicities (below 1000 μg/mL) are negligible to the MG-63 osteoblast-like cells in the evaluated period, confirming they are nontoxic and would not inhibit the growth of the cells. However, the corresponding small molecules EISA and EIN showed cytotoxicity potency and could inhibit cell proliferation at all concentrations except for EIN at 100 μg/mL. A 24 h incubation with 1000 μg/mL of EISA and EIN inflicted severe cell death in the MG-63 osteoblast-like cells. Moreover, a dose-dependent cytotoxic effect was observed for small molecules EISA and EIN under the experimental conditions. For EISA, cell viability started to decrease under the lowest tested dose (100 μg/mL), which was significantly lower under 500 and 1000 μg/mL with doses (11.15% and 10.53% respectively). For EIN, there was no significant influence of the cell viability under a 100 μg/mL dose, but consistently cell viability decreased when higher doses were applied. From these observations, it was concluded that the grafting of isatin and indole groups on the BH40 polymer backbone did not lead to a significant cytotoxic effect to MG-63 osteoblast-like cells.

## 4 Conclusions

This study presents the synthesis and evaluation of new biodegradable hyperbranched nonionic polyesters with indole and isatin groups as nonleachable antibacterial agents, which could potentially be used as additives or coatings. A key finding is that while both isatin and indole groups can endow strong antibacterial properties to the hyperbranched polyesters, the presence of a hydrogen-bond donor (i.e., N–H) in indole remarkably enhanced the miscibility of the resulting polymers with biodegradable polyester matrices (PHB, PCL). Furthermore, we discovered that the presence of indole groups facilitated the enzymatic degradation of ester bonds in both HBP and its monomeric model compound, while the presence of isatin groups had a complex impact on the PETase-catalyzed hydrolysis. Molecular docking simulations showed that the presence of indole and isatin rings could form hydrogen bonds with the PETase backbone, thus promoting different orientations of the molecules at the active site. MTT assay revealed that the obtained polymeric antimicrobials showed negligible cytotoxicity, while small molecular analogs showed a significant cytotoxic effect. We also observed anti-quorum sensing effects for the obtained nonionic polymers, which may suggest investigations toward an antibiofilm effect in the future.

## Supporting Information Available

Synthesis scheme and analytical data of grafting agent **1** (^1^H NMR spectrum); analytical data of grafting agents **1** and **2** (FTIR and TGA data) and polymers (^13^C NMR spectra); grafting density analysis; photos of leaching potential tests; DSC data of polymer blends; illustration of hydrogen bonding; synthesis and analytical data of *m*-BIN (^1^H NMR spectrum, DSC data); Petri plate images of disk diffusion assay; antibacterial data of monomers and polymers (inhibition zone data, *p* values, anti-QS data); biodegradation data of polymers (LC-MS data, chemical structure of the observed degradation products) and monomers (calibration curves, HPLC data) (PDF)

## References

[ref1] Nederberg F.; Zhang Y.; Tan J. P. K.; Xu K.; Wang H.; Yang C.; Gao S.; Guo X. D.; Fukushima K.; Li L.; et al. Biodegradable Nanostructures with Selective Lysis of Microbial Membranes. Nat. Chem. 2011, 3, 409–414. 10.1038/nchem.1012. 21505501

[ref2] Hook A. L.; Chang C. Y.; Yang J.; Luckett J.; Cockayne A.; Atkinson S.; Mei Y.; Bayston R.; Irvine D. J.; Langer R.; et al. Combinatorial Discovery of Polymers Resistant to Bacterial Attachment. Nat. Biotechnol. 2012, 30 (9), 868–875. 10.1038/nbt.2316. 22885723 PMC3796337

[ref3] Chin W.; Zhong G.; Pu Q.; Yang C.; Lou W.; De Sessions P. F.; Periaswamy B.; Lee A.; Liang Z. C.; Ding X.; et al. A Macromolecular Approach to Eradicate Multidrug Resistant Bacterial Infections While Mitigating Drug Resistance Onset. Nat. Commun. 2018, 9, 1–14. 10.1038/s41467-018-03325-6. 29500445 PMC5834525

[ref4] Zhu T.; Sha Y.; Yan J.; Pageni P.; Rahman M. A.; Yan Y.; Tang C. Metallo-Polyelectrolytes as a Class of Ionic Macromolecules for Functional Materials. Nat. Commun. 2018, 9, 917 10.1038/s41467-018-06475-9. 30337530 PMC6193978

[ref5] Rahman M. A.; Bam M.; Luat E.; Jui M. S.; Ganewatta M. S.; Shokfai T.; Nagarkatti M.; Decho A. W.; Tang C. Macromolecular-Clustered Facial Amphiphilic Antimicrobials. Nat. Commun. 2018, 9 (1), 1–10. 10.1038/s41467-018-07651-7. 30531920 PMC6286373

[ref6] Tew G. N.; Scott R. W.; Klein M. L.; DeGrado W. F. De Novo Design of Antimicrobial Polymers, Foldamers, and Small Molecules: From Discovery to Practical Applications. Acc. Chem. Res. 2010, 43, 30–39. 10.1021/ar900036b. 19813703 PMC2808429

[ref7] Kenawy E.-R.; Worley S. D.; Broughton R. The Chemistry and Applications of Antimicrobial Polymers: A State-of-the-Art Review. Biomacromolecules 2007, 8, 1359–1384. 10.1021/bm061150q. 17425365

[ref8] Siedenbiedel F.; Tiller J. C. Antimicrobial Polymers in Solution and on Surfaces: Overview and Functional Principles. Polymers (Basel, Switz.) 2012, 4, 46–71. 10.3390/polym4010046.

[ref9] Banerjee I.; Pangule R. C.; Kane R. S. Antifouling Coatings: Recent Developments in the Design of Surfaces That Prevent Fouling by Proteins, Bacteria, and Marine Organisms. Adv. Mater. 2011, 23, 690–718. 10.1002/adma.201001215. 20886559

[ref10] Santos M. R. E.; Fonseca A. C.; Mendonça P. V.; Branco R.; Serra A. C.; Morais P. V.; Coelho J. F. J. Recent Developments in Antimicrobial Polymers: A Review. Materials 2016, 9 (7), 599–632. 10.3390/ma9070599. PMC5456892 28773721

[ref11] Zhu Z.; Jeong G.; Kim S. J.; Gadwal I.; Choe Y.; Bang J.; Oh M. K.; Khan A.; Rao J. Balancing Antimicrobial Performance with Hemocompatibility in Amphiphilic Homopolymers. J. Polym. Sci., Part A: Polym. Chem. 2018, 56, 2391–2396. 10.1002/pola.29213.

[ref12] Khatoon Z.; McTiernan C. D.; Suuronen E. J.; Mah T. F.; Alarcon E. I. Bacterial Biofilm Formation on Implantable Devices and Approaches to Its Treatment and Prevention. Heliyon 2018, 4 (12), e01067 10.1016/j.heliyon.2018.e01067. 30619958 PMC6312881

[ref13] Abat C.; Gautret P.; Raoult D. Benefits of Antibiotics Burden in Low-Income Countries. Proc. Natl. Acad. Sci. U. S. A. 2018, 115 (35), E8109–E8110. 10.1073/pnas.1809354115. 30087189 PMC6126769

[ref14] Zhao C.; Liu A.; Santamaria C. M.; Shomorony A.; Ji T.; Wei T.; Gordon A.; Elofsson H.; Mehta M.; Yang R.; et al. Polymer-Tetrodotoxin Conjugates to Induce Prolonged Duration Local Anesthesia with Minimal Toxicity. Nat. Commun. 2019, 10 (1), 2566 10.1038/s41467-019-10296-9. 31189915 PMC6561913

[ref15] Robert C.; De Montigny F.; Thomas C. M. Tandem Synthesis of Alternating Polyesters from Renewable Resources. Nat. Commun. 2011, 2 (1), 1–6. 10.1038/ncomms1596. PMC3247812 22158441

[ref16] Sangroniz A.; Zhu J.-B.; Tang X.; Etxeberria A.; Chen E. Y.-X.; Sardon H. Packaging Materials with Desired Mechanical and Barrier Properties and Full Chemical Recyclability. Nat. Commun. 2019, 10 (1), 1–7. 10.1038/s41467-019-11525-x. 31395871 PMC6687705

[ref17] Lutz J. F.; Andrieu J.; Üzgün S.; Rudolph C.; Agarwal S. Biocompatible, Thermoresponsive, and Biodegradable: Simple Preparation of “All-in-One” Biorelevant Polymers. Macromolecules 2007, 40 (24), 8540–8543. 10.1021/ma7021474.

[ref18] Kanai T.; Thirumoolan D.; Mohanram R.; Vetrivel K.; Basha K. A. Antimicrobial Activity of Hyperbranched Polymers: Synthesis, Characterization, and Activity Assay Study. J. Bioact. Compat. Polym. 2015, 30 (2), 145–156. 10.1177/0883911514565936.

[ref19] Izutsu K.; Shigeo K. Phase Separation of Polyelectrolytes and Non-Ionic Polymers in Frozen Solutions. Phys. Chem. Chem. Phys. 2000, 2, 123–127. 10.1039/a907591g.

[ref20] Liber K.; Weber L.; Lévesque C. Sublethal Toxicity of Two Wastewater Treatment Polymers to Lake Trout Fry (Salvelinus Namaycush). Chemosphere 2005, 61 (8), 1123–1133. 10.1016/j.chemosphere.2005.03.004. 16263382

[ref21] Costa R.; Pereira J. L.; Gomes J.; Gonçalves F.; Hunkeler D.; Rasteiro M. G. The Effects of Acrylamide Polyelectrolytes on Aquatic Organisms: Relating Toxicity to Chain Architecture. Chemosphere 2014, 112, 177–184. 10.1016/j.chemosphere.2014.03.096. 25048904

[ref22] Cumming J. L.; Hawker D. W.; Matthews C.; Chapman H. F.; Nugent K. Analysis of Polymeric Quaternary Ammonium Salts as Found in Cosmetics by Metachromatic Polyelectrolyte Titration. Toxicol. Environ. Chem. 2010, 92 (9), 1595–1608. 10.1080/02772248.2010.482062.

[ref23] Demircan D.; Zhang B. Facile Synthesis of Novel Soluble Cellulose-Grafted Hyperbranched Polymers as Potential Natural Antimicrobial Materials. Carbohydr. Polym. 2017, 157, 1913–1921. 10.1016/j.carbpol.2016.11.076. 27987911

[ref24] Karpagam S.; Guhanathan S. Phosphorus Based Indole and Imidazole Functionalized Hyperbranched Polyester as Antimicrobial Surface Coating Materials. Prog. Org. Coat. 2014, 77 (11), 1901–1910. 10.1016/j.porgcoat.2014.06.022.

[ref25] Boopathy M.; Selvam R.; JohnSanthoshkumar S.; Subramanian K. Synthesis and Evaluation of Polyacrylamides Derived from Polycyclic Pendant Naphthalene, Indole, and Phenothiazine Based Chalcone Moiety as Potent Antimicrobial Agents. Polym. Adv. Technol. 2017, 28 (6), 717–727. 10.1002/pat.3972.

[ref26] Arza C. R.; Ilk S.; Demircan D.; Zhang B. New Biobased Non-Ionic Hyperbranched Polymers as Environmentally Friendly Antibacterial Additives for Biopolymers. Green Chem. 2018, 20 (6), 1238–1249. 10.1039/C7GC03401F.

[ref27] Cornell R. J.; Donaruma L. G. 2-MethacryIoxytropones. Intermediates for the Synthesis of Biologically Active Polymers. J. Med. Chem. 1965, 8 (3), 388–390. 10.1021/jm00327a025. 14323155

[ref28] Erdmann L.; Uhrich K. E. Synthesis and Degradation Characteristics of Salicylic Acid-Derived Poly(Anhydride-Esters). Biomaterials 2000, 21 (19), 1941–1946. 10.1016/S0142-9612(00)00073-9. 10941915

[ref29] Jabara R.; Chronos N.; Robinson K. Novel Bioabsorbable Salicylate-Based Polymer as a Drug-Eluting Stent Coating. Catheter. Cardiovasc. Interv. 2008, 72 (2), 186–194. 10.1002/ccd.21607. 18651646

[ref30] Shpaisman N.; Sheihet L.; Bushman J.; Winters J.; Kohn J. One-Step Synthesis of Biodegradable Curcumin-Derived Hydrogels as Potential Soft Tissue Fillers after Breast Cancer Surgery. Biomacromolecules 2012, 13 (8), 2279–2286. 10.1021/bm300518e. 22703560

[ref31] Hauenstein O.; Agarwal S.; Greiner A. Bio-Based Polycarbonate as Synthetic Toolbox. Nat. Commun. 2016, 7 (May), 1–7. 10.1038/ncomms11862. PMC4912624 27302694

[ref32] Weintraub S.; Shpigel T.; Harris L. G.; Schuster R.; Lewis E. C.; Lewitus D. Y. Astaxanthin-Based Polymers as New Antimicrobial Compounds. Polym. Chem. 2017, 8 (29), 4182–4189. 10.1039/C7PY00663B.

[ref33] Wang D.; Zhao T.; Zhu X.; Yan D.; Wang W. Bioapplications of Hyperbranched Polymers. Chem. Soc. Rev. 2015, 44 (12), 4023–4071. 10.1039/C4CS00229F. 25176339

[ref34] Gurunathan T.; Mohanty S.; Nayak S. K. Hyperbranched Polymers for Coating Applications: A Review. Polym.-Plast. Technol. Eng. 2016, 55, 92–117. 10.1080/03602559.2015.1021482.

[ref35] Mintzer M. A.; Dane E. L.; O’Toole G. A.; Grinstaff M. W. Exploiting Dendrimer Multivalency to Combat Emerging and Reemerging Infectious Diseases. Mol. Pharmaceutics 2012, 9, 342–354. 10.1021/mp2005033. PMC3729585 22126461

[ref36] Ortega P.; Cobaleda B. M. A.; Hernández-Ros J. M.; Fuentes-Paniagua E.; Sánchez-Nieves J.; Tarazona M. P.; Copa-Patiño J. L.; Soliveri J.; De La Mata F. J.; Gómez R. Hyperbranched Polymers versus Dendrimers Containing a Carbosilane Framework and Terminal Ammonium Groups as Antimicrobial Agents. Org. Biomol. Chem. 2011, 9 (14), 5238–5248. 10.1039/c1ob05321c. 21629893

[ref37] Calabretta M. K.; Kumar A.; McDermott A. M.; Cai C. Antibacterial Activities of Poly(Amidoamine) Dendrimers Terminated with Amino and Poly(Ethylene Glycol) Groups. Biomacromolecules 2007, 8 (6), 1807–1811. 10.1021/bm0701088. 17511499 PMC2430505

[ref38] Kwak S. Y.; Ahn D. U. Processability of Hyperbranched Poly(Ether Ketone)s with Different Degrees of Branching from Viewpoints of Molecular Mobility and Comparison with Their Linear Analogue. Macromolecules 2000, 33 (20), 7557–7563. 10.1021/ma991569r.

[ref39] Li J.; Zhang T.; Liang Y.; Yang R. Solution-Processible Carbazole Dendrimers as Host Materials for Highly Efficient Phosphorescent Organic Light-Emitting Diodes. Adv. Funct. Mater. 2013, 23, 619–628. 10.1002/adfm.201201326.

[ref40] Shih H. M.; Wu R. C.; Shih P. I.; Wang C. L.; Hsu C. S. Synthesis of Fluorene-Based Hyperbranched Polymers for Solution-Processable Blue, Green, Red, and White Light-Emitting Devices. J. Polym. Sci., Part A: Polym. Chem. 2012, 50, 696–710. 10.1002/pola.25080.

[ref41] Chen C. Z.; Cooper S. L. Interactions between Dendrimer Biocides and Bacterial Membranes. Biomaterials 2002, 23, 3359–3368. 10.1016/S0142-9612(02)00036-4. 12099278

[ref42] Ortega P.; Copa-Patiño J. L.; Muñoz-Fernandez M. A.; Soliveri J.; Gomez R.; de la Mata F. J. Amine and Ammonium Functionalization of Chloromethylsilane-Ended Dendrimers. Antimicrobial Activity Studies. Org. Biomol. Chem. 2008, 6 (18), 3264 10.1039/b809569h. 18802631

[ref43] Abid C. K. V. Z.; Chattopadhyay S.; Mazumdar N.; Singh H. Synthesis and Characterization of Quaternary Ammonium PEGDA Dendritic Copolymer Networks for Water Disinfection. J. Appl. Polym. Sci. 2010, 116 (3), 1640–1649. 10.1002/app.31510.

[ref44] Charles S.; Vasanthan N.; Kwon D.; Sekosan G.; Ghosh S. Surface Modification of Poly (Amidoamine)(PAMAM) Dendrimer as Antimicrobial Agents. Tetrahedron Lett. 2012, 53 (49), 6670–6675. 10.1016/j.tetlet.2012.09.098. 23125468 PMC3486433

[ref45] Gangadharan D.; Dhandhala N.; Dixit D.; Thakur R. S.; Popat K. M.; Anand P. S. Investigation of Solid Supported Dendrimers for Water Disinfection. J. Appl. Polym. Sci. 2012, 124 (2), 1384–1391. 10.1002/app.34967.

[ref46] Fuentes-Paniagua E.; Hernández-Ros J. M.; Sánchez-Milla M.; Camero M. A.; Maly M.; Pérez-Serrano J.; Copa-Patiño J. L.; Sánchez-Nieves J.; Soliveri J.; Gómez R.; et al. Carbosilane Cationic Dendrimers Synthesized by Thiol-Ene Click Chemistry and Their Use as Antibacterial Agents. RSC Adv. 2014, 4 (3), 1256–1265. 10.1039/C3RA45408H.

[ref47] Bakhshi H.; Agarwal S. Dendrons as Active Clicking Tool for Generating Non-Leaching Antibacterial Materials. Polym. Chem. 2016, 7 (33), 5322–5330. 10.1039/C6PY01105E.

[ref48] Bakhshi H.; Agarwal S. Hyperbranched Polyesters as Biodegradable and Antibacterial Additives. J. Mater. Chem. B 2017, 5 (33), 6827–6834. 10.1039/C7TB01301A. 32264332

[ref49] Bakhshi H.; Agarwal S. Hyperbranched Polyesters as Biodegradable and Antibacterial Additives. J. Mater. Chem. B 2017, 5 (33), 6827–6834. 10.1039/C7TB01301A. 32264332

[ref50] Mukherjee I.; Ghosh A.; Bhadury P.; De P. Leucine-Based Polymer Architecture-Induced Antimicrobial Properties and Bacterial Cell Morphology Switching. ACS Omega 2018, 3 (1), 769–780. 10.1021/acsomega.7b01674. 30023789 PMC6044967

[ref51] Cao H.; Zheng Y.; Zhou J.; Wang W.; Pandit A. A Novel Hyperbranched Polyester Made from Aconitic Acid (B3) and Di(Ethylene Glycol) (A2). Polym. Int. 2011, 60 (4), 630–634. 10.1002/pi.2993.

[ref52] Santra S.; Kaittanis C.; Perez J. M. Aliphatic Hyperbranched Polyester: A New Building Block in the Construction of Multifunctional Nanoparticles and Nanocomposites. Langmuir 2010, 26 (8), 5364–5373. 10.1021/la9037843. 19957939 PMC2854188

[ref53] Huang Y.; Wang D.; Zhu X.; Yan D.; Chen R. Synthesis and Therapeutic Applications of Biocompatible or Biodegradable Hyperbranched Polymers. Polym. Chem. 2015, 6 (15), 2794–2812. 10.1039/C5PY00144G.

[ref54] Aryal S.; Prabaharan M.; Pilla S.; Gong S. Biodegradable and Biocompatible Multi-Arm Star Amphiphilic Block Copolymer as a Carrier for Hydrophobic Drug Delivery. Int. J. Biol. Macromol. 2009, 44 (4), 346–352. 10.1016/j.ijbiomac.2009.01.007. 19428465

[ref55] Yoshida S.; Hiraga K.; Takehana T.; Taniguchi I.; Yamaji H.; Maeda Y.; Toyohara K.; Miyamoto K.; Kimura Y.; Oda K. A Bacterium That Degrades and Assimilates Poly(Ethyleneterephthalate). Science (Washington, DC, U. S.) 2016, 351, 1196–1199. 10.1126/science.aad6359. 26965627

[ref56] Royter M.; Schmidt M.; Elend C.; Höbenreich H.; Schäfer T.; Bornscheuer U. T.; Antranikian G. Thermostable Lipases from the Extreme Thermophilic Anaerobic Bacteria Thermoanaerobacter Thermohydrosulfuricus SOL1 and Caldanaerobacter Subterraneus Subsp. Tengcongensis. Extremophiles 2009, 13, 769–783. 10.1007/s00792-009-0265-z. 19579003 PMC2757599

[ref57] Wagner-Egea P., Wang P.; Grey C.; Zhang B.; Linares-Pastén J. A. PETase Degradation Assessment of Terephthalate Aromatic Polyesters. Manuscript in preparation, 2020.

[ref58] Wang P.; Arza C. R.; Zhang B. Indole as a New Sustainable Aromatic Unit for High Quality Biopolyesters. Polym. Chem. 2018, 9, 4706–4710. 10.1039/C8PY00962G.

[ref59] Arza C. R.; Wang P.; Linares-Pastén J.; Zhang B. Synthesis, Thermal, Rheological Characteristics, and Enzymatic Degradation of Aliphatic Polyesters with Lignin-based Aromatic Pendant Groups. J. Polym. Sci., Part A: Polym. Chem. 2019, 57, 2314–2323. 10.1002/pola.29534.

[ref60] Wang P.; Linares-Pastén J.; Zhang B. Synthesis, Molecular Docking Simulation and Enzymatic Degradation of AB-Type Indole-Based Polyesters with Improved Thermal Properties. Biomacromolecules 2020, 21 (3), 1078–1090. 10.1021/acs.biomac.9b01399. 31951388

[ref61] Hanwell M. D.; Curtis D. E.; Lonie D. C.; Vandermeersch T.; Zurek E.; Hutchison G. R. Avogadro: An Advanced Semantic Chemical Editor, Visualization, and Analysis Platform. J. Cheminf. 2012, 4, 1–17. 10.1186/1758-2946-4-17. PMC3542060 22889332

[ref62] Joo S.; Cho I. J.; Seo H.; Son H. F.; Sagong H. Y.; Shin T. J.; Choi S. Y.; Lee S. Y.; Kim K. J. Structural Insight into Molecular Mechanism of Poly(Ethylene Terephthalate) Degradation. Nat. Commun. 2018, 9, 382 10.1038/s41467-018-02881-1. 29374183 PMC5785972

[ref63] Trott O.; Olson A. J. Autodock Vina: Improving the Speed and Accuracy of Docking. J. Comput. Chem. 2010, 31 (2), 455–461. 10.1002/jcc.21334. 19499576 PMC3041641

[ref64] Krieger E.; Vriend G. YASARA View - Molecular Graphics for All Devices - from Smartphones to Workstations. Bioinformatics 2014, 30 (20), 2981–2982. 10.1093/bioinformatics/btu426. 24996895 PMC4184264

[ref65] Pettersen E. F.; Goddard T. D.; Huang C. C.; Couch G. S.; Greenblatt D. M.; Meng E. C.; Ferrin T. E. UCSF Chimera — A Visualization System for Exploratory Research and Analysis. J. Comput. Chem. 2004, 25, 1605–1612. 10.1002/jcc.20084. 15264254

[ref66] Demircan D.; Ilk S.; Zhang B. Cellulose-Organic Montmorillonite Nanocomposites as Biomacromolecular Quorum-Sensing Inhibitor. Biomacromolecules 2017, 18, 3439–3446. 10.1021/acs.biomac.7b01116. 28841299

[ref67] Shmidt M. S.; Perillo I. A.; Camelli A.; Fernández M. A.; Blanco M. M. Polyfunctional 4-Quinolinones. Synthesis of 2-Substituted 3-Hydroxy-4-Oxo-1,4-Dihydroquinolines. Tetrahedron Lett. 2016, 57, 1022–1026. 10.1016/j.tetlet.2016.01.077.

[ref68] Yi Mok N.; Chadwick J.; Kellett K. A.B.; Hooper N. M.; Johnson A. P.; Fishwick C. W.G. Bioorganic & Medicinal Chemistry Letters Discovery of Novel Non-Peptide Inhibitors of BACE-1 Using Virtual High-Throughput Screening. Bioorg. Med. Chem. Lett. 2009, 19, 6770–6774. 10.1016/j.bmcl.2009.09.103. 19854048

[ref69] Tsakos M.; Schaffert E. S.; Clement L. L.; Villadsen N. L.; Poulsen T. B. Ester Coupling Reactions - an Enduring Challenge in the Chemical Synthesis of Bioactive Natural Products. Nat. Prod. Rep. 2015, 32, 605–632. 10.1039/C4NP00106K. 25572105

[ref70] Yu H.; Schlüter A. D.; Zhang B. Synthesis of Dendronized Polymers by a “n + 2” Approach. Macromolecules 2012, 45, 8555–8560. 10.1021/ma301982r.

[ref71] Žagar E.; Žigon M.; Podzimek S. Characterization of Commercial Aliphatic Hyperbranched Polyesters. Polymer 2006, 47 (1), 166–175. 10.1016/j.polymer.2005.10.142.

[ref72] Margoshes M.; Fassel V.A. The Infrared spectra of Aromatic Compounds I. The out-of-Plane C-H Bending Vibrations in the Region 625–900 Cm-1. Spectrochim. Acta 1955, 7, 14–24. 10.1016/0371-1951(55)80003-3.

[ref73] Liu T.; Simmons T. L.; Bohnsack D. A.; Mackay M. E.; Smith M. R.; Baker G. L. Synthesis of Polymandelide: A Degradable Polylactide Derivative with Polystyrene-like Properties. Macromolecules 2007, 40, 6040–6047. 10.1021/ma061839n.

[ref74] Chan R. T. H.; Garvey C. J.; Marçal H.; Russell R. A.; Holden P. J.; Foster L. J. R. Manipulation of Polyhydroxybutyrate Properties through Blending with Ethyl-Cellulose for a Composite Biomaterial. Int. J. Polym. Sci. 2011, 2011, 651549 10.1155/2011/651549.

[ref75] Galasso V.; Pappalardo G. C. Dipole Moments and Absorption Spectra of Heterocyclic Diketones. J. Chem. Soc., Perkin Trans. 2 1976, 0 (5), 574 10.1039/p29760000574.

[ref76] Parkanyi C.; Oruganti S. R.; Abdelhamid A. O.; Von Szentpaly L.; Ngom B.; Aaron J.-J. Dipole Moments of Indoles in Their Ground and the First Excited Singlet States. J. Mol. Struct.: THEOCHEM 1986, 135, 105–116. 10.1016/0166-1280(86)80050-1.

[ref77] Žagar E.; Huskić M.; Grdadolnik J.; Žigon M.; Zupančič-Valant A. Effect of Annealing on the Rheological and Thermal Properties of Aliphatic Hyperbranched Polyester Based on 2,2-Bis(Methylol) Propionic Acid. Macromolecules 2005, 38, 3933–3942. 10.1021/ma0475434.

[ref78] Yang D.; Kong J. 100% Hyperbranched Polymers via the Acid-Catalyzed Friedel-Crafts Aromatic Substitution Reaction. Polym. Chem. 2016, 7 (33), 5226–5232. 10.1039/C6PY01168C.

[ref79] Keshavarz T.; Roy I. Polyhydroxyalkanoates: Bioplastics with a Green Agenda. Curr. Opin. Microbiol. 2010, 13 (3), 321–326. 10.1016/j.mib.2010.02.006. 20227907

[ref80] Fuchs A.; Youssef A.; Seher A.; Hochleitner G.; Dalton P. D.; Hartmann S.; Brands R. C.; Müller-Richter U. D. A.; Linz C. Medical-Grade Polycaprolactone Scaffolds Made by Melt Electrospinning Writing for Oral Bone Regeneration - A Pilot Study in Vitro. BMC Oral Health 2019, 19 (1), 1–11. 10.1186/s12903-019-0717-5. 30709394 PMC6359770

[ref81] Suttiwijitpukdee N.; Sato H.; Zhang J.; Hashimoto T.; Ozaki Y. Intermolecular Interactions and Crystallization Behaviors of Biodegradable Polymer Blends between Poly (3-Hydroxybutyrate) and Cellulose Acetate Butyrate Studied by DSC, FT-IR, and WAXD. Polymer 2011, 52 (2), 461–471. 10.1016/j.polymer.2010.11.021.

[ref82] Khasanah; Reddy K. R.; Sato H.; Takahashi I.; Ozaki Y. Intermolecular Hydrogen Bondings in the Poly(3-Hydroxybutyrate) and Chitin Blends: Their Effects on the Crystallization Behavior and Crystal Structure of Poly(3-Hydroxybutyrate). Polymer 2015, 75, 141–150. 10.1016/j.polymer.2015.08.011.

[ref83] Barham P. J.; Keller A.; Otun E. L.; Holmes P. A. Crystallization and Morphology of a Bacterial Thermoplastic: Poly-3-Hydroxybutyrate. J. Mater. Sci. 1984, 19, 2781–2794. 10.1007/BF01026954.

[ref84] Wei L.; Stark N. M.; McDonald A. G. Interfacial Improvements in Biocomposites Based on Poly(3-Hydroxybutyrate) and Poly(3-Hydroxybutyrate-Co-3-Hydroxyvalerate) Bioplastics Reinforced and Grafted with α-Cellulose Fibers. Green Chem. 2015, 17, 4800–4814. 10.1039/C5GC01568E.

[ref85] Crescenzi V.; Manzini G.; Calzolari G.; Borri C. Thermodynamics of Fusion of Poly-β-Propiolactone and Poly-β-Caprolactone. Comparative Analysis of the Melting of Aliphatic Polylactone and Polyester Chains. Eur. Polym. J. 1972, 8 (3), 449–463. 10.1016/0014-3057(72)90109-7.

[ref86] Azuma Y.; Yoshie N.; Sakurai M.; Inoue Y.; Chûjô R. Thermal Behaviour and Miscibility of Poly(3-Hydroxybutyrate)/Poly(Vinyl Alcohol) Blends. Polymer 1992, 33, 4763–4767. 10.1016/0032-3861(92)90690-X.

[ref87] Janigová I.; Lacík I.; Chodák I. Thermal Degradation of Plasticized Poly(3-Hydroxybutyrate) Investigated by DSC. Polym. Degrad. Stab. 2002, 77, 35–41. 10.1016/S0141-3910(02)00077-0.

[ref88] Iglesias Montes M. L.; D’amico D. A.; Manfredi L. B.; Cyras V. P. Effect of Natural Glyceryl Tributyrate as Plasticizer and Compatibilizer on the Performance of Bio-Based Polylactic Acid/Poly(3-Hydroxybutyrate) Blends. J. Polym. Environ. 2019, 27 (7), 1429–1438. 10.1007/s10924-019-01425-y.

[ref89] Abdelwahab M. A.; Flynn A.; Chiou B.-S.; Imam S.; Orts W.; Chiellini E. Thermal, Mechanical and Morphological Characterization of Plasticized PLA-PHB Blends. Polym. Degrad. Stab. 2012, 97, 1822–1828. 10.1016/j.polymdegradstab.2012.05.036.

[ref90] Chan C. H.; Kummerlowe C.; Kammer H.-W. Crystallization and Melting Behavior of Poly(3-Hydroxybutyrate)-Based Blends. Macromol. Chem. Phys. 2004, 205, 664–675. 10.1002/macp.200300062.

[ref91] Kuo S. W.; Chan S. C.; Chang F. C. Effect of Hydrogen Bonding Strength on the Microstructure and Crystallization Behavior of Crystalline Polymer Blends. Macromolecules 2003, 36 (17), 6653–6661. 10.1021/ma034695a.

[ref92] Luyt A. S.; Gasmi S. Influence of Blending and Blend Morphology on the Thermal Properties and Crystallization Behaviour of PLA and PCL in PLA/PCL Blends. J. Mater. Sci. 2016, 51 (9), 4670–4681. 10.1007/s10853-016-9784-z.

[ref93] Khunová V.; Kelnar I.; Kristóf J.; Dybal J.; Kratochvíl J.; Kaprálková L. The Effect of Urea and Urea-Modified Halloysite on Performance of PCL. J. Therm. Anal. Calorim. 2015, 120 (2), 1283–1291. 10.1007/s10973-015-4448-9.

[ref94] Lu X.; Weiss R. A. Relationship between the Glass Transition Temperature and the Interaction Parameter of Miscible Binary Polymer Blends. Macromolecules 1992, 25 (12), 3242–3246. 10.1021/ma00038a033.

[ref95] Chaudhuri A.; Haldar S.; Sun H.; Koeppe R. E.; Chattopadhyay A. Importance of Indole NH Hydrogen Bonding in the Organization and Dynamics of Gramicidin Channels. Biochim. Biophys. Acta, Biomembr. 2014, 1838 (1), 419–428. 10.1016/j.bbamem.2013.10.011. PMC3975908 24148157

[ref96] Wagner V. E.; Bushnell D.; Passador L.; Brooks A. I.; Iglewski B. H. Microarray Analysis of Pseudomonas Aeruginosa Quorum-Sensing Regulons: Effects of Growth Phase and Environment. J. Bacteriol. 2003, 185 (7), 2080–2095. 10.1128/JB.185.7.2080-2095.2003. 12644477 PMC151498

[ref97] Yan S.; Wu G. Can Biofilm Be Reversed Through Quorum Sensing in Pseudomonas Aeruginosa?. Front. Microbiol. 2019, 10 (July), 1–9. 10.3389/fmicb.2019.01582. 31396166 PMC6664025

[ref98] Klare W.; Das T.; Ibugo A.; Buckle E.; Manefield M.; Manos J. Glutathione-Disrupted Biofilms of Clinical Pseudomonas Aeruginosa Strains Exhibit an Enhanced Antibiotic Effect and a Novel Biofilm Transcriptome. Antimicrob. Agents Chemother. 2016, 60 (8), 4539–4551. 10.1128/AAC.02919-15. 27161630 PMC4958218

[ref99] Wolska K. I.; Grudniak A. M.; Rudnicka Z.; Markowska K. Genetic Control of Bacterial Biofilms. J. Appl. Genet. 2016, 57 (2), 225–238. 10.1007/s13353-015-0309-2. 26294280 PMC4830867

[ref100] Tjernberg A.; Markova N.; Griffiths W. J.; Hallén D. DMSO-Related Effects in Protein Characterization. J. Biomol. Screening 2006, 11 (2), 131–137. 10.1177/1087057105284218. 16490773

[ref101] Fecker T.; Galaz-Davison P.; Engelberger F.; Narui Y.; Sotomayor M.; Parra L. P.; Ramírez-Sarmiento C. A. Active Site Flexibility as a Hallmark for Efficient PET Degradation by I. Sakaiensis PETase. Biophys. J. 2018, 114 (6), 1302–1312. 10.1016/j.bpj.2018.02.005. 29590588 PMC5883944

[ref102] Zhu B.; Wei N. Biocatalytic Degradation of Parabens Mediated by Cell Surface Displayed Cutinase. Environ. Sci. Technol. 2019, 53 (1), 354–364. 10.1021/acs.est.8b05275. 30507170

[ref103] Hyatt J. L.; Moak T.; Hatfield M. J.; Tsurkan L.; Edwards C. C.; Wierdl M.; Danks M. K.; Wadkins R. M.; Potter P. M. Selective Inhibition of Carboxylesterases by Isatins, Indole-2,3-Diones. J. Med. Chem. 2007, 50 (8), 1876–1885. 10.1021/jm061471k. 17378546

