# Supplementary material for: Synthesis, Enzymatic Degradation, and Polymer-Miscibility Evaluation of Nonionic Antimicrobial Hyperbranched Polyesters with Indole or Isatin Functionalities
Source: Biomacromolecules. 2021 Apr 26;22(5):2256–71. doi: 10.1021/acs.biomac.1c00343 (PMC8382248; doi:10.1021/acs.biomac.1c00343)
Supplement: Supplementary file 1 — bm1c00343_si_001.pdf [file bm1c00343_si_001.pdf]

Synthesis, enzymatic degradation and polymer-miscibility evaluation of non-ionic antimicrobial hyperbranched polyesters with indole or isatin functionalities

Xiaoya Li,[a] Sedef İlk,[b,c] Javier A. Linares-Pastén,[d] Yang Liu,[e] Deepak Bushan Raina,[e] Deniz Demircan,[a] Baozhong Zhang[*a]*

[a] Lund University, Centre for Analysis and Synthesis, Department of Chemistry, P. O. Box 124, SE-22100 Lund, Sweden

[b] Faculty of Medicine, Department of Immunology, Niğde Ömer Halisdemir University, 51240 Niğde, Turkey

[c] KTH Royal Institute of Technology, School of Engineering Sciences in Chemistry, Biotechnology and Health, Department of Chemistry, Division of Glycoscience, SE-10691 Stockholm, Sweden

[d] Division of Biotechnology, Department of Chemistry, Lund University, P. O. Box 124, 22100 Lund, Sweden

[e] Faculty of Medicine, Department of Clinical Sciences, Orthopedics, Lund University, Lund, Sweden

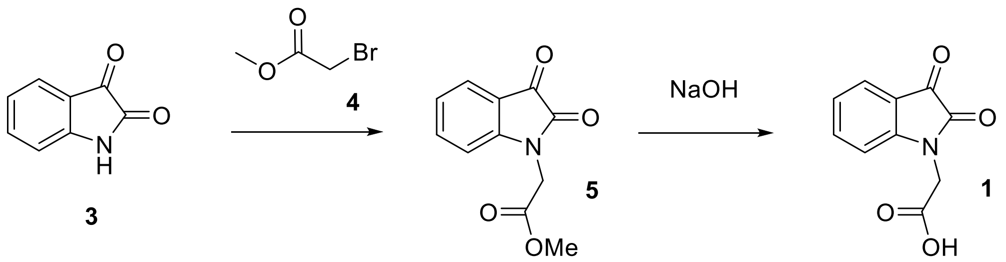

Scheme S1. Synthesis of grafting agent **1** from isatin (**3**).

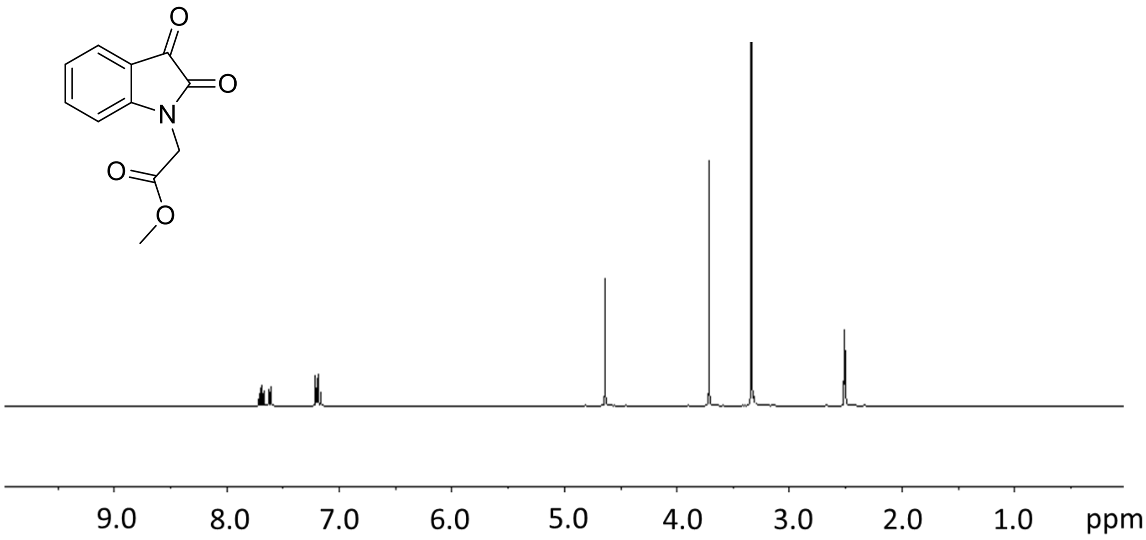

Figure S1. ^{1}H-NMR spectrum of methyl isatin-N-acetate (**5**).

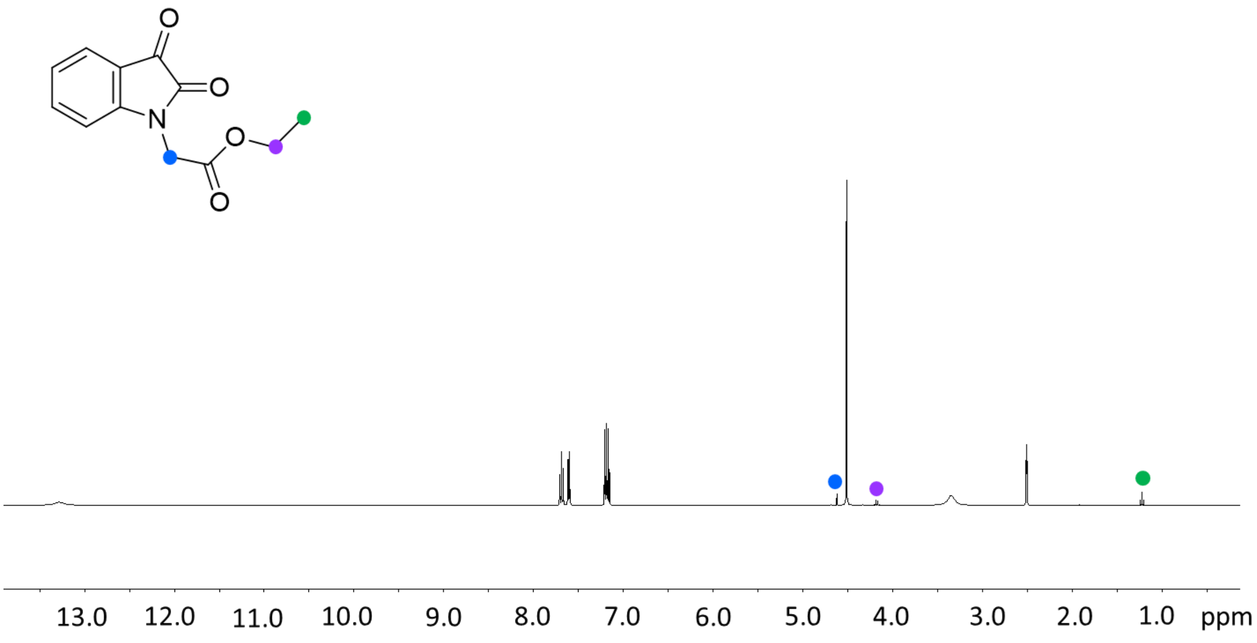

Figure S2. ^{1}H-NMR spectrum of by-product ethyl isatin-N-acetate.

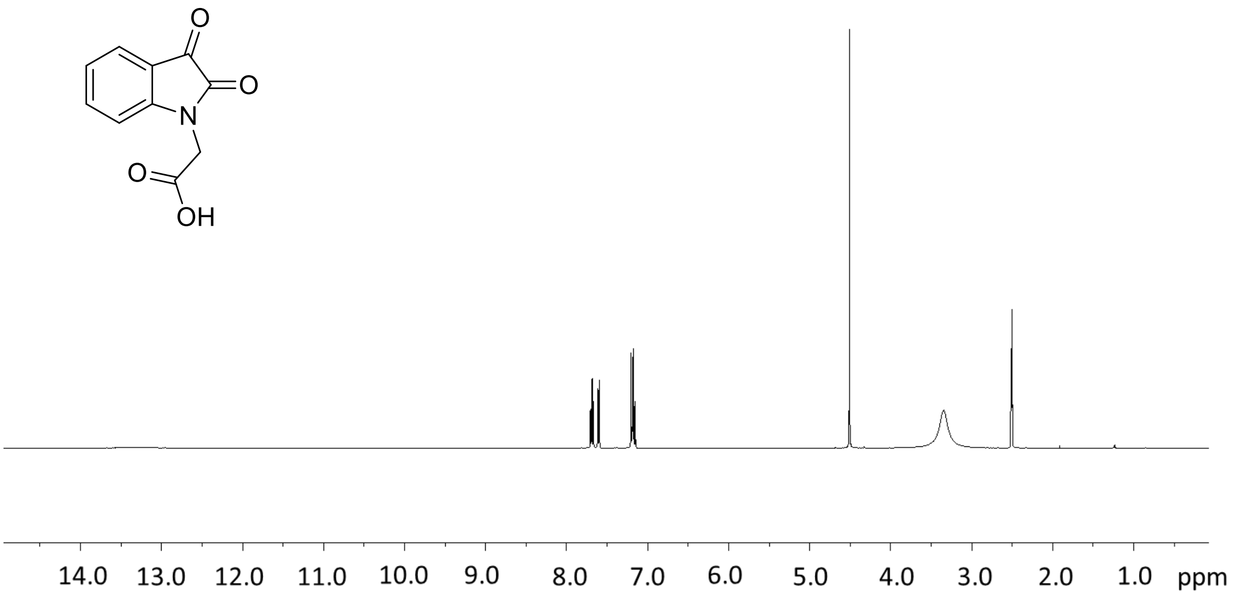

Figure S3. ^{1}H-NMR spectrum of purified isatin-N-acetic acid (**1**).

Table S1. Effect of initial excess of grafting agents (**1** and **2**) on the grafting density and yield of the resulting HBPs. Grafting density (GD) means the percentage of grafted OH groups among all the OH groups in the products BIN and BISA, which is obtained from ^{1}H-NMR. GD^1, GD^2 and GD^3 were calculated by comparing the integrals of different NMR signals (Fig. S4). GD is the average value of GD^1, GD^2 and GD^3. The yield means the isolated yield of BIN and BISA after purification.

HBP	Excess of grafting agent (**1** or **2**)/mol	GD^1 (%)	GD^2 (%)	GD^3 (%)	GD (%)	Yield (%)
BISA	10%	81	75	78	78	42
	50%	82	81	81	81	30
	200%	95	92	93	93	26
BIN	10%	80	82	81	81	45
	50%	98	93	90	94	29
	200%	98	95	90	94	27

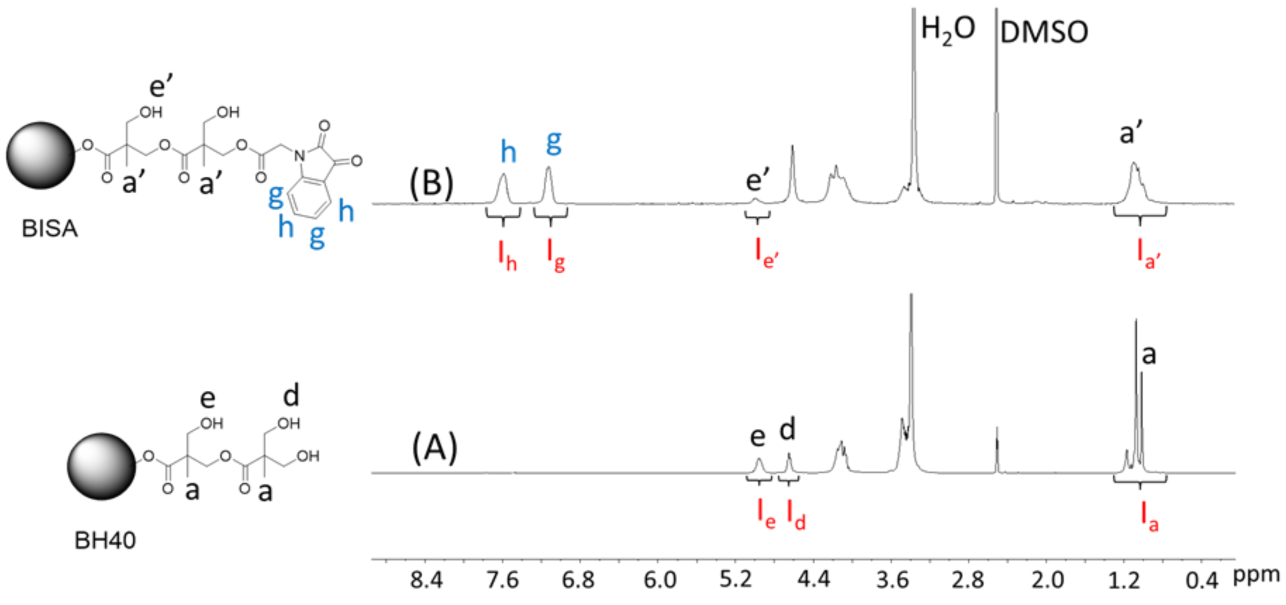

Figure S4. ^{1}H-NMR spectra of (A) BH40 and (B) BISA synthesized with 10 mol% initial excess of grafting agent **1**. The integrals for the calculation of grafting density (GD^1, GD^2 and GD^3) are marked. The grafting density values of BISA were calculated according to the equations below and the results are shown in Table S1. The integrals of the two spectra are normalized to make $I_a = I_{a'}$ (suppose that the methyl groups did not change before and after the grafting reaction).

$$GD^1 = 1 - \frac{I_{e'}}{I_d + I_e}$$

$$GD^2 = \frac{I_g}{2} / \frac{I_a}{3}$$

$$GD^3 = \frac{I_h}{2} / \frac{I_a}{3}$$

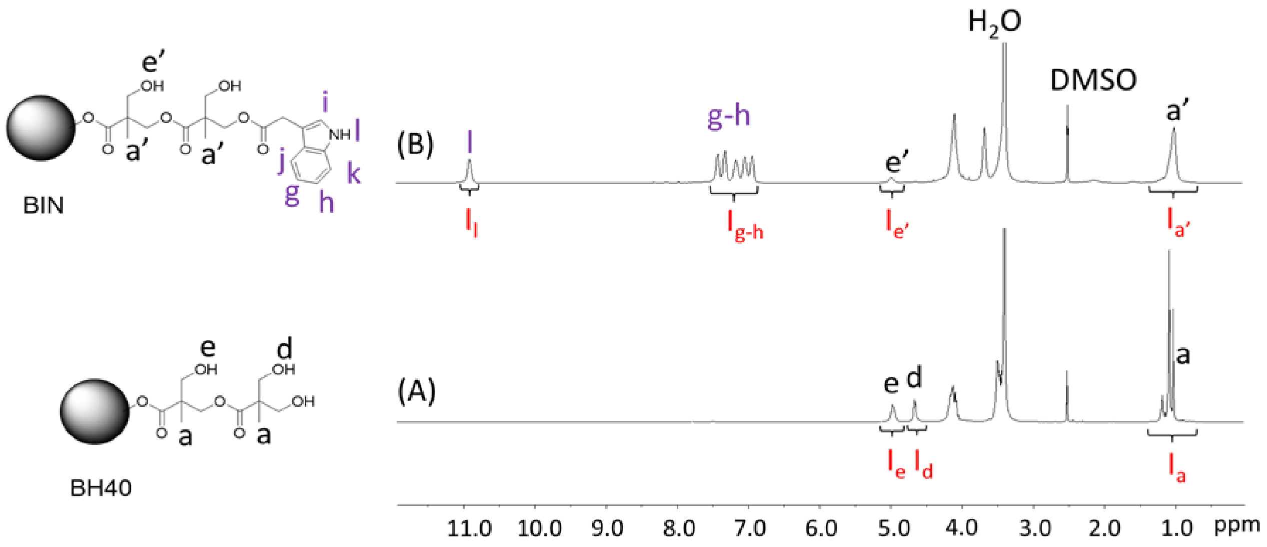

Figure S5. ^{1}H-NMR spectra of (A) BH40 and (B) BIN synthesized with 10 mol% initial excess of grafting agent **2**. The integrals for the calculation of grafting density (GD^1, GD^2 and GD^3) are marked. The grafting density values of BIN were calculated according to the equations below and the results are shown in Table S1. The integrals of the two spectra are normalized to make $I_a = I_{a'}$ (suppose that the methyl groups did not change before and after the grafting reaction).

$$GD^1 = 1 - \frac{I_{e'}}{I_d + I_e}$$

$$GD^2 = \frac{I_{g-h}}{5} / \frac{I_a}{3}$$

$$GD^3 = \frac{I_l}{1} / \frac{I_a}{3}$$

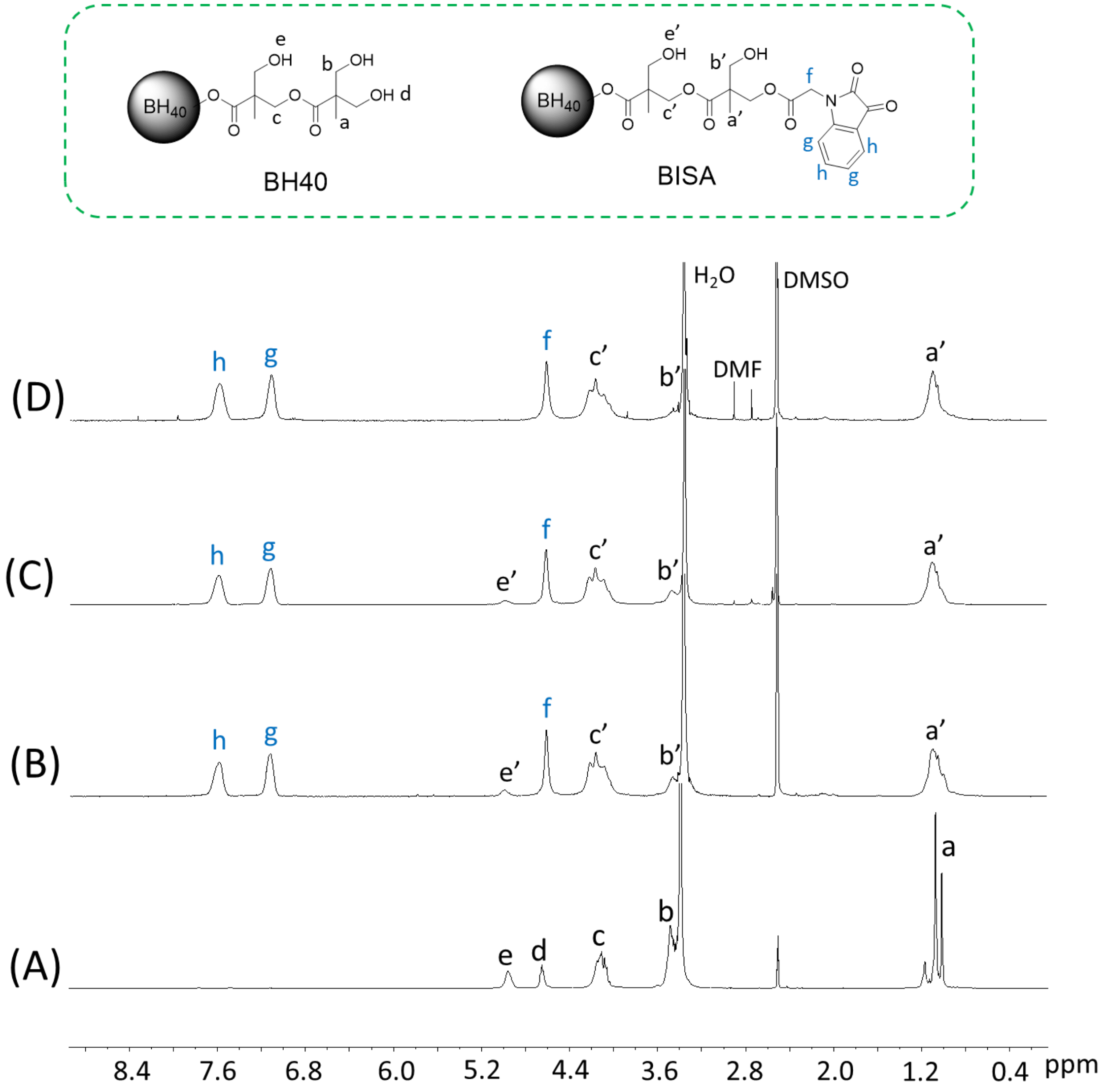

Figure S6. ^{1}H-NMR spectra of (A) BH40 and BISA synthesized with (B) 10%, (C) 50%, and (D) 200% initial excess of grafting agent **1**. The integrals of all the spectra are normalized to make $I_a=I_{a'}$ (suppose that the methyl groups did not change before and after the grafting reaction). The grafting density of the BISA was calculated according to the equations in Fig. S4, and the results are shown in Table S1.

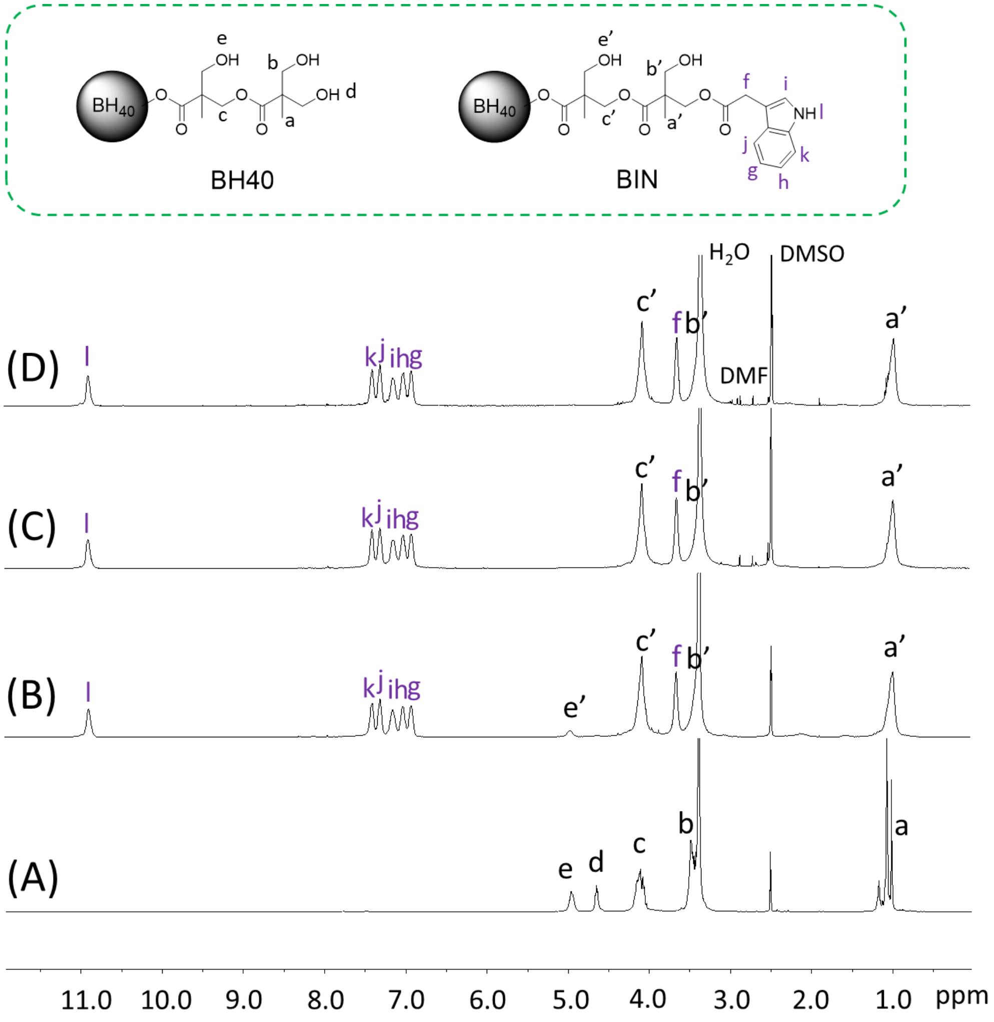

Figure S7. ^{1}H-NMR spectra of (A) BH40 and BIN synthesized with 10% (B), 50% (C), and 200% (D) initial excess of grafting agent **2**. The integrals of all the spectra are normalized to make $I_a=I_{a'}$ (suppose that the methyl groups did not change before and after the grafting reaction). The grafting density of the BIN was calculated according to the equations in Fig. S5, and the results are shown in Table S1.

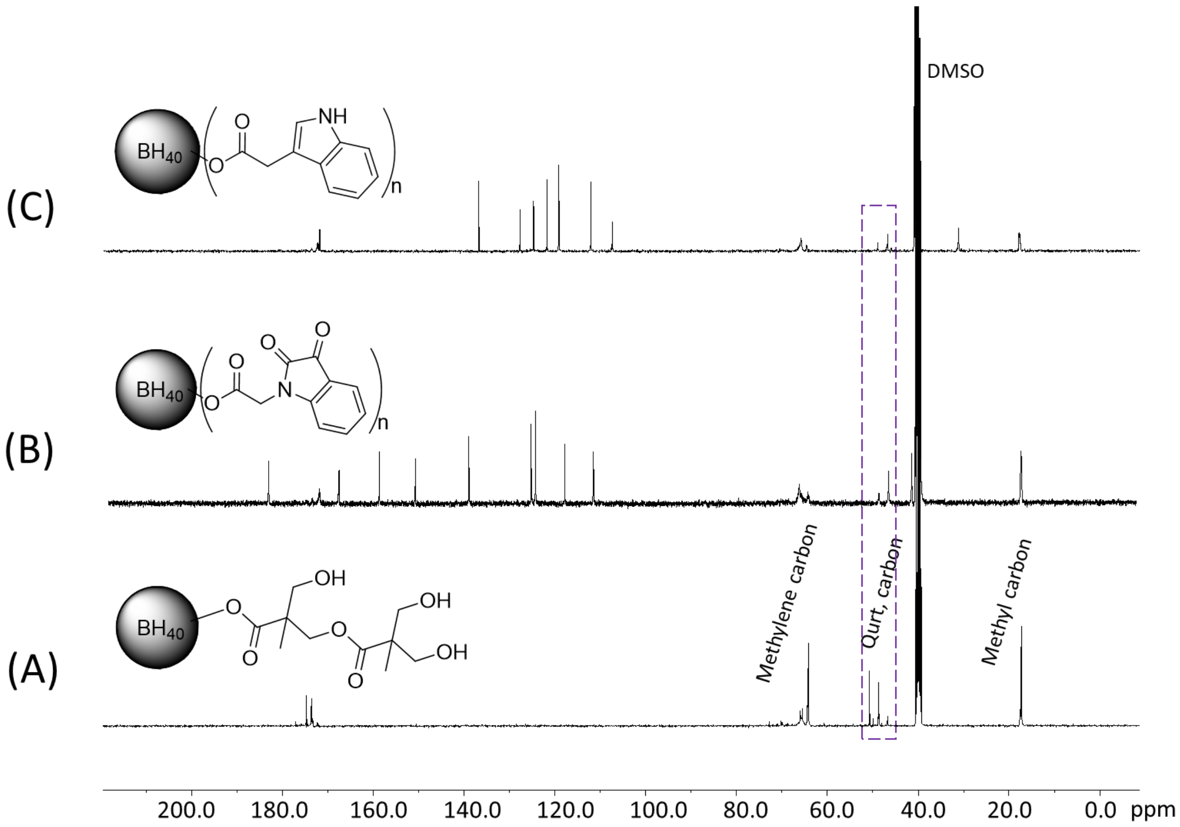

Figure S8. ^{13}C-NMR spectra of (A) BH40, (B) BISA and (C) BIN.

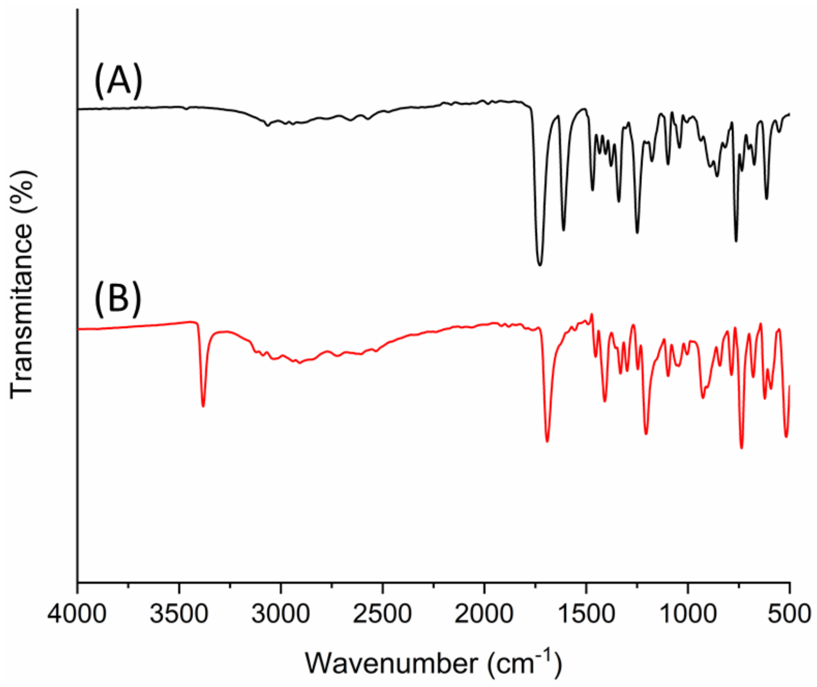

Figure S9. FT-IR spectra of the grafting agents (A) **1**, and (B) **2**.

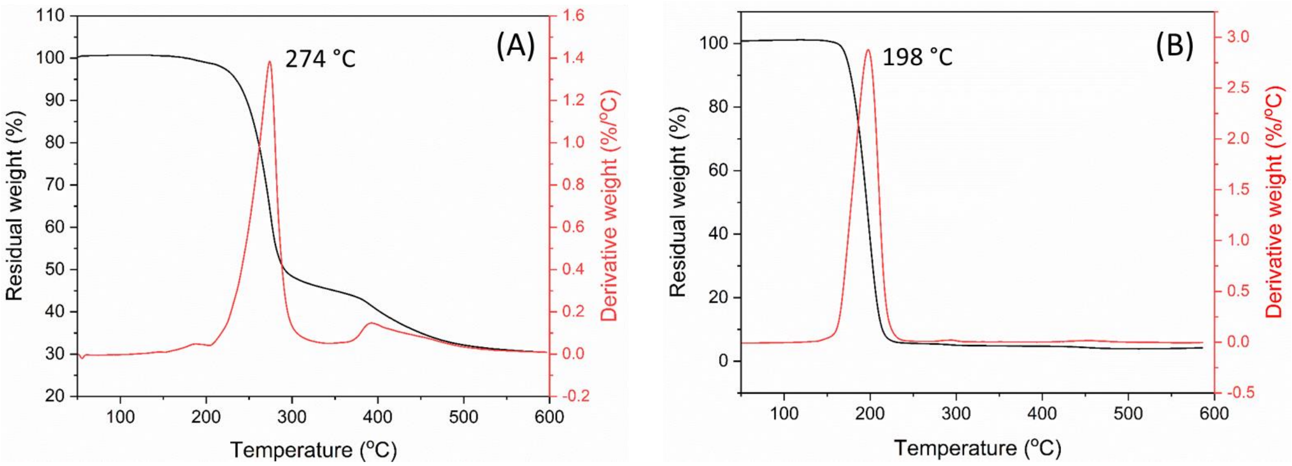

Figure S10. Residual weight and derivative weight loss cures of (A) **1**, and (B) **2**.

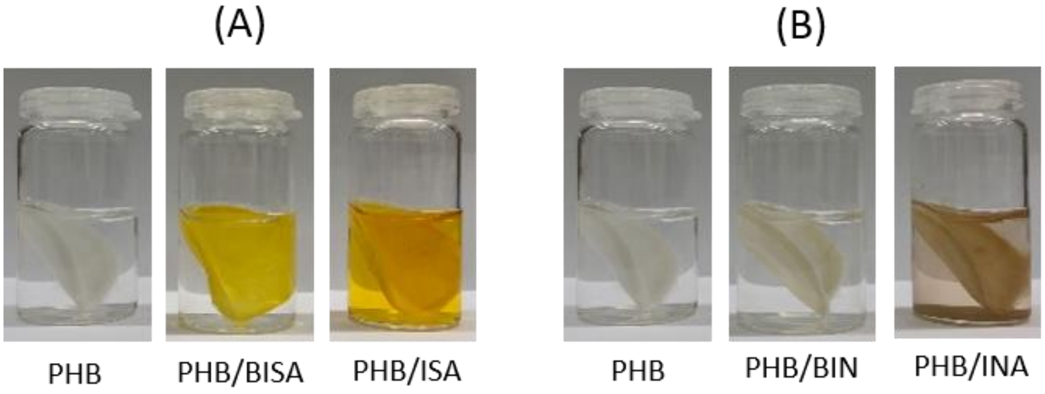

Figure S11. Photos of PHB films after being merged in deionized water for 5 days, for evaluation of the leaching potential of different polymers and molecules from PHB matrix. (A) from left: pure PHB, PHB with 5 w% of BISA, and PHB with 5 w% isatin (ISA). (B) from left: pure PHB, PHB with 5 w% of BIN, and PHB with 5w% indole-3-acetic acid (INA). Note, the orange/yellow color of the PHB/BISA and PHB/ISA blended films originated from the color of BISA and ISA.

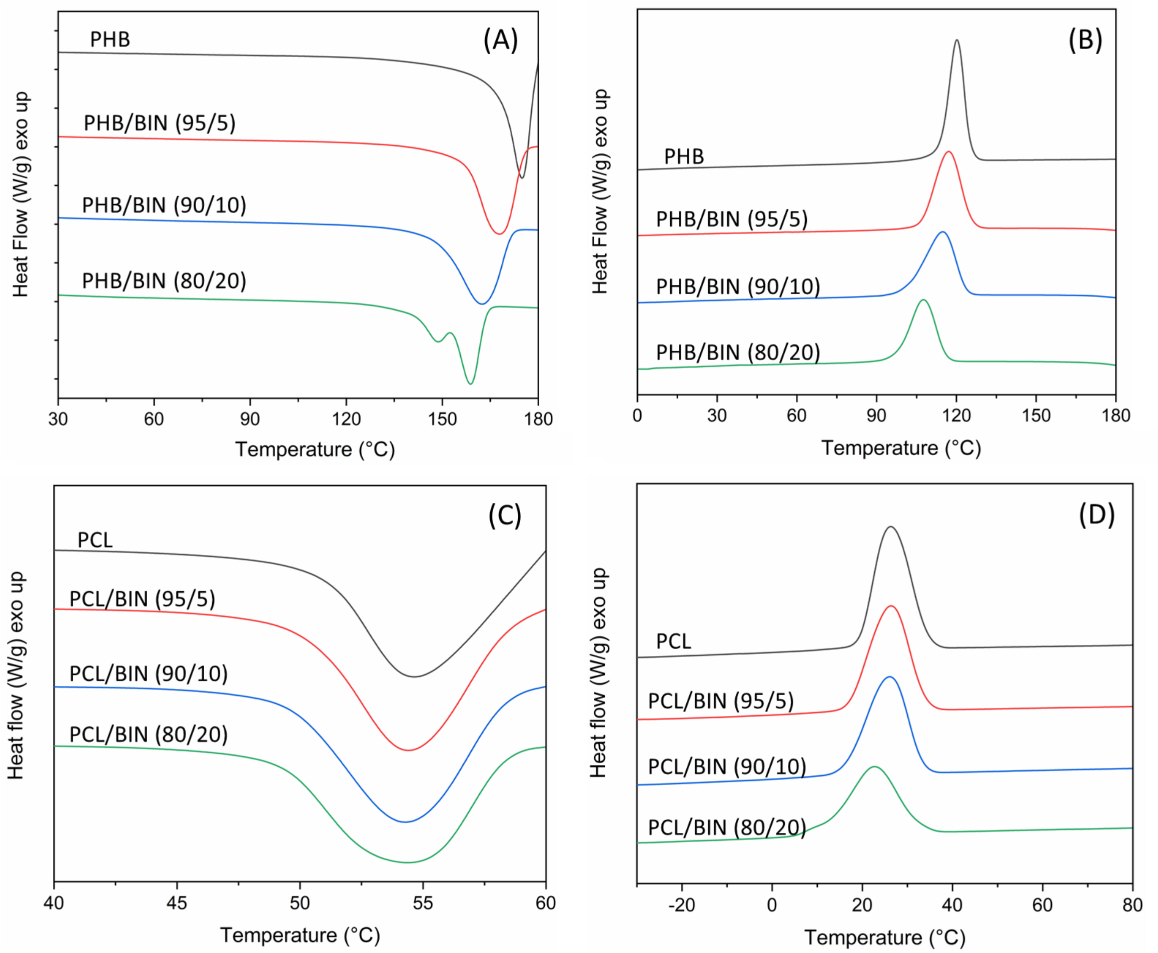

Figure S12. DSC second (A) second heating and (B) first cooling curves of PHB/BIN blends, and (C) second heating and (D) first cooling curves of PCL/BIN blends.

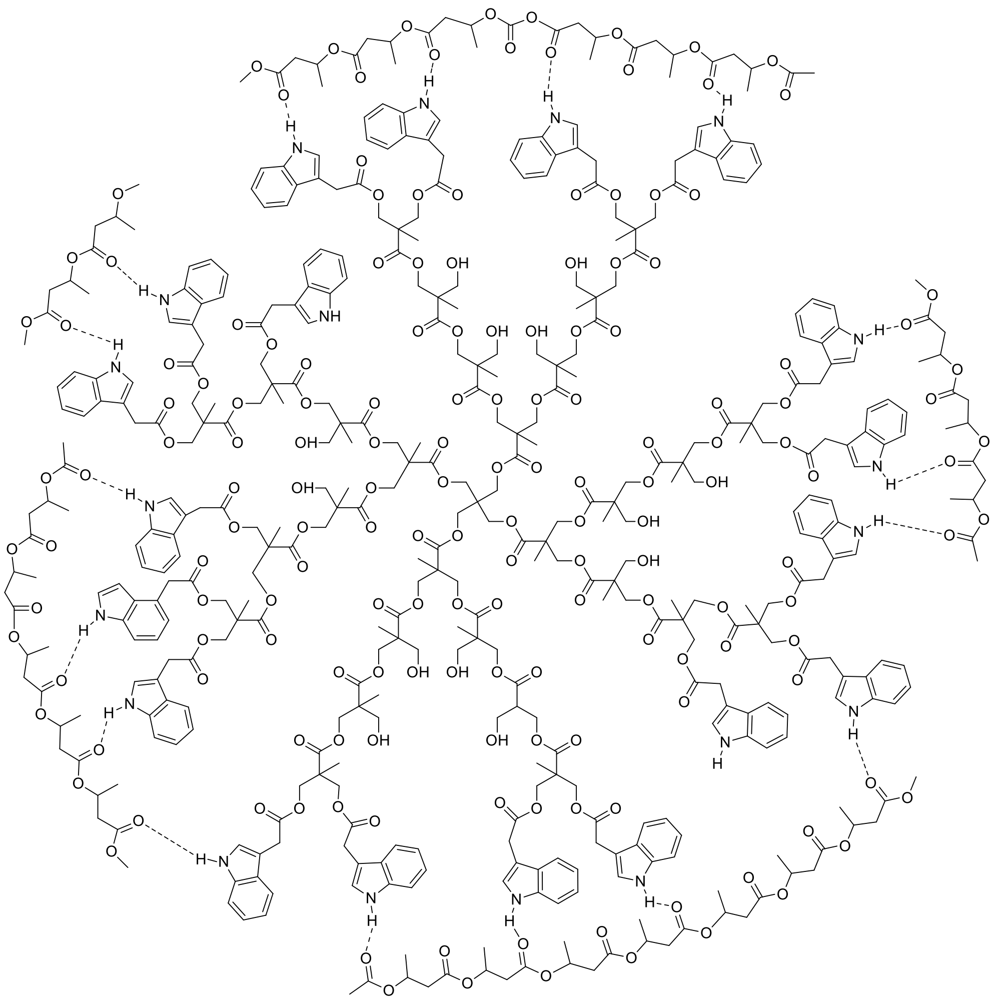

Figure S13. Hydrogen bonding between PHB and BIN.

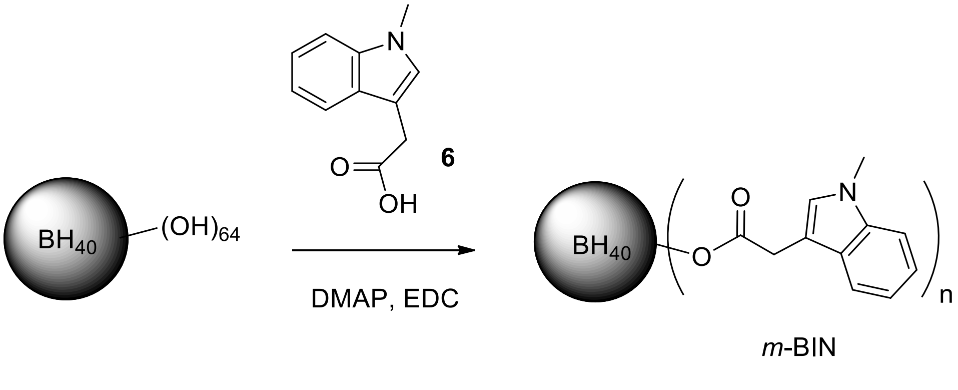

Scheme S2. Synthesis of N-methylindole grafted HBP, namely *m*-BIN.

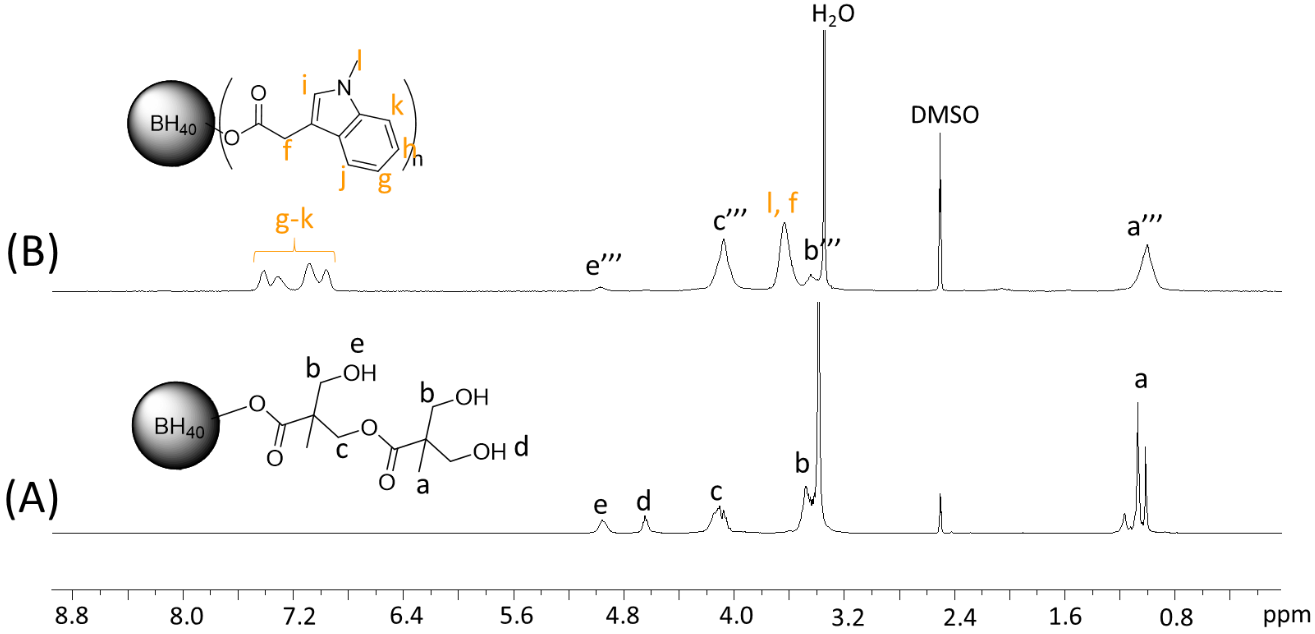

Figure S14. ^{1}H-NMR spectra of (A) BH40 and (B) *m*-BIN in DMSO-d$_6$.

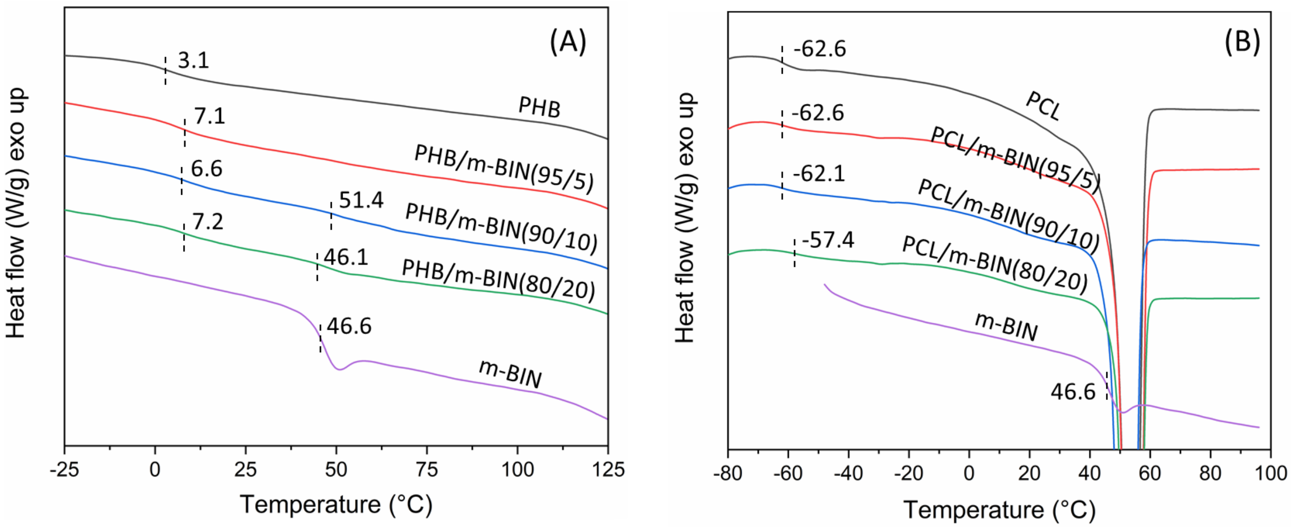

Figure S15. DSC second heating curves of (A) neat PHB and PHB with 5, 10 and 20 w% of *m*-BIN and neat *m*-BIN; (B) neat PCL and PCL with 5, 10 and 20 w% of *m*-BIN.

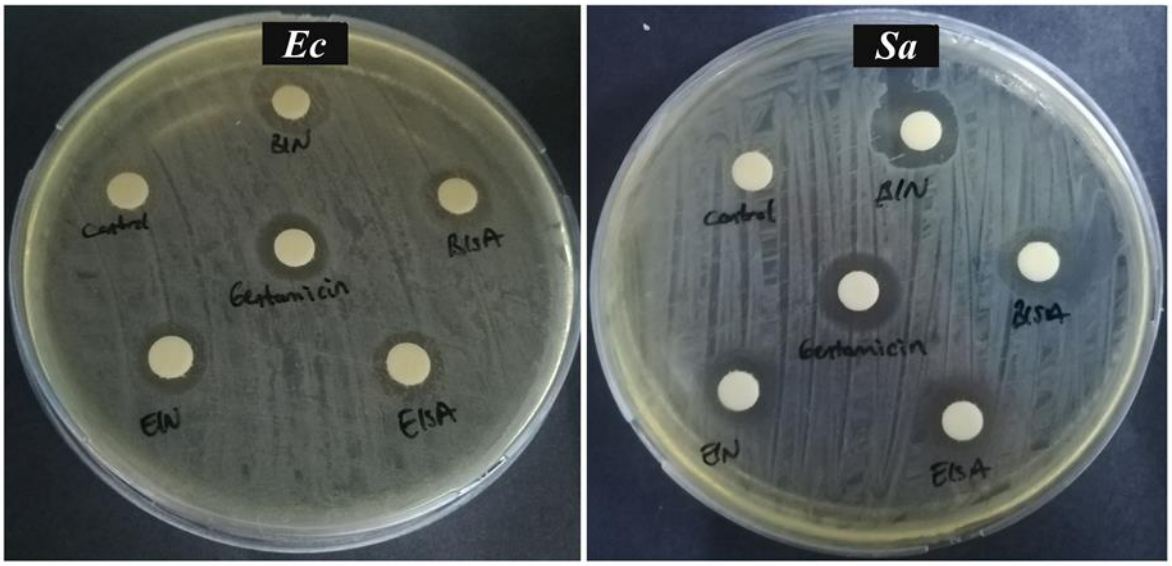

Figure S16. Petri plate images of disk diffusion assay for BIN, EIN, BISA, EISA, gentamicin and control (only solvent DMF) against *Escherichia coli* (G-) and *Staphylococcus aureus* (G+).

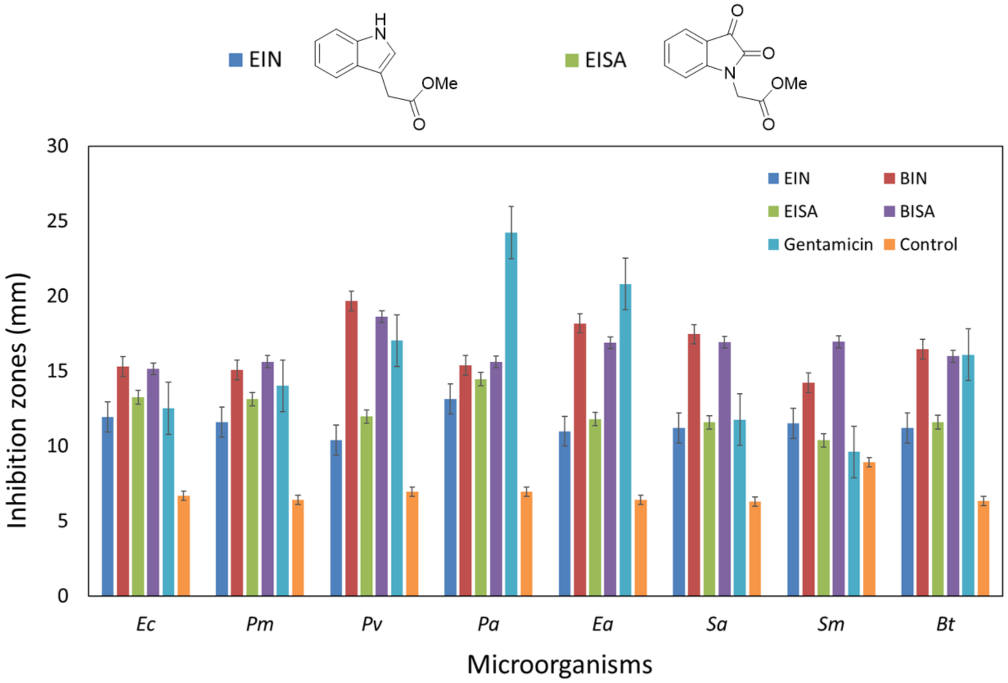

Figure S17. Comparison of the antibacterial effects of HBPs (BIN and BISA) and small molecular agents (EIN, EISA and gentamicin) at the loading level of 10 μg per disk. DMF was used for the negative control experiments.

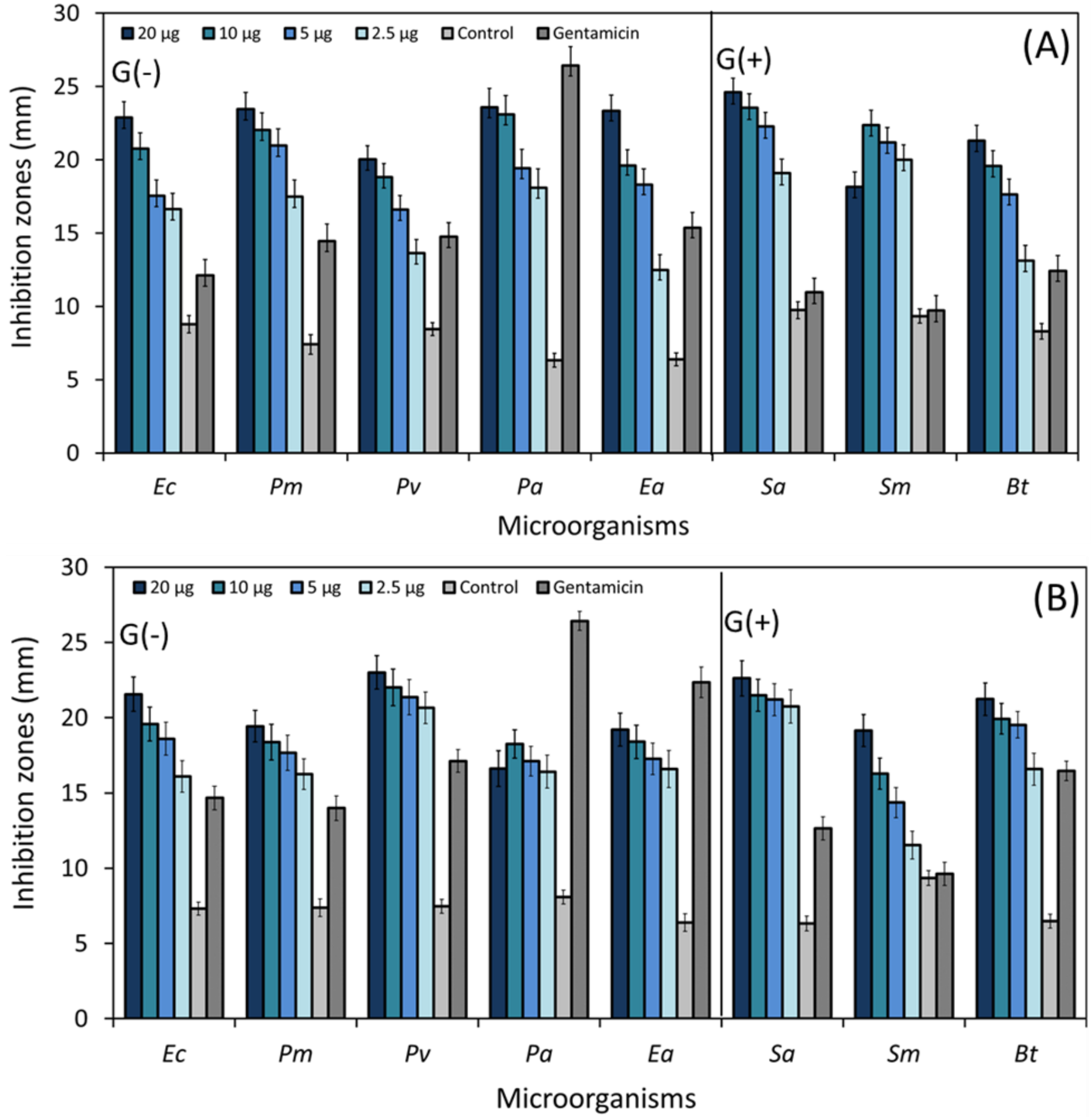

Figure S18. Comparison of antibacterial effects of BISA (A) and BIN (B) at different loading amount per disk. In the negative control experiments, pure DMF was used to treat the sample. In the positive control, gentamicin (10 μg per disk) was used to treat the samples.

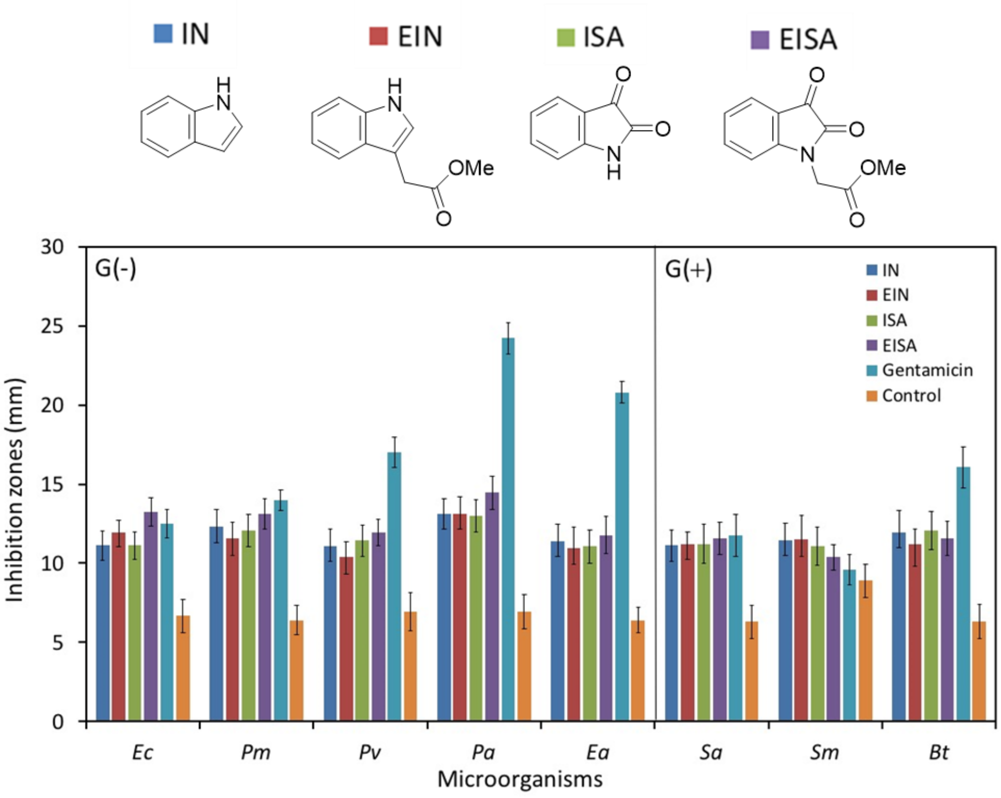

Figure 19. Comparison of the antibacterial effects of small molecules with indole or isatin groups. Pure DMF was used as negative control (shown as control in the figure). Gentamicin was used as positive control.

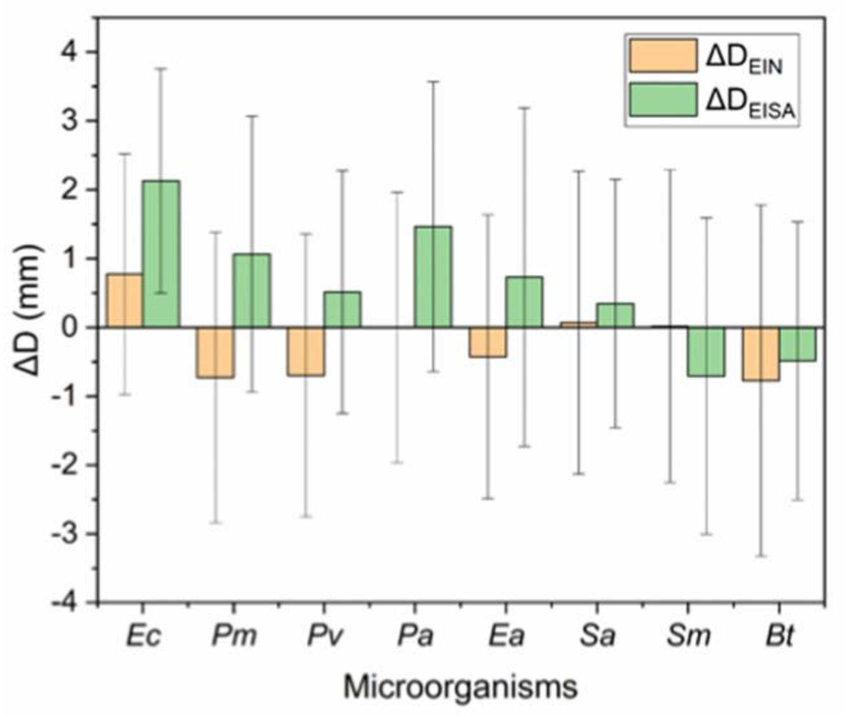

Figure S20. Comparison of small functional molecules with or without ester bonds. The differences in the zones of inhibition ($\Delta D_{EIN} = D_{EIN} - D_{IN}$, $\Delta D_{EISA} = D_{EISA} - D_{ISA}$). D_{IN}, D_{EIN}, D_{ISA} and D_{EISA} are the diameters of the inhibition zones of IN, EIN, ISA and EISA, respectively (chemical structures shown in Fig. S19).

Table S2. *p* values calculated by *t*-test statistical analysis for the comparison of the antibacterial effects between two samples (indicated in each entry).

		p value							
Entry	Sample	*Ec*	*Pm*	*Pv*	*Pa*	*Ea*	*Sa*	*Sm*	*Bt*
1	BISA/BIN	0.786	0.570	0.285	0.803	0.230	0.674	0.083	0.600
2	BISA/EISA	0.037	0.039	0.001	0.251	0.005	0.006	0.002	0.007
3	BIN/EIN	0.007	0.015	0.000	0.056	0.001	0.005	0.078	0.004
4	BISA/gentamicin	0.018	0.110	0.129	0.001	0.010	0.007	0.001	0.916
5	BIN/gentamicin	0.018	0.250	0.037	0.001	0.049	0.006	0.012	0.668
6	EISA/ISA	0.033	0.263	0.516	0.164	0.508	0.665	0.511	0.587
7	EIN/IN	0.339	0.447	0.453	0.997	0.639	0.942	0.987	0.500

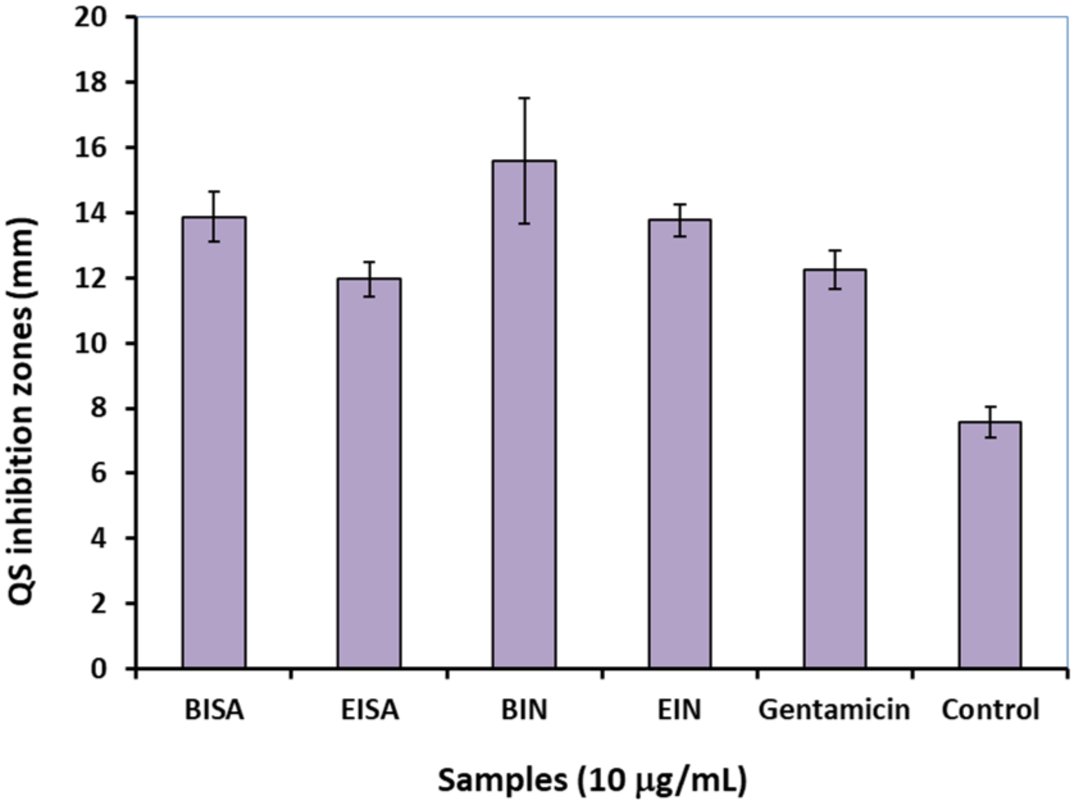

Figure S21. The anti-quorum sensing (anti-QS) property of BISA, EISA, BIN, and EIN at loading amount of 10 μg/mL against *Chromobacterium violaceum* CV026. Pure DMF was used as the negative control. Gentamicin (10 μg per disk) was used as the positive control.

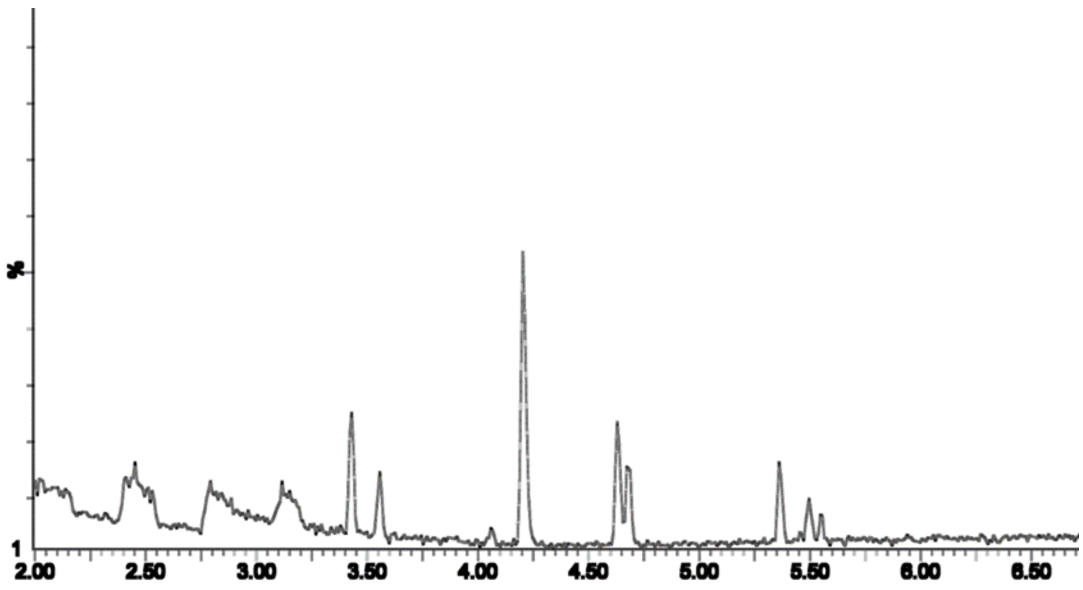

Figure S22. Base peak chromatograms (LC-MS) of the negative control experiment for BIN degradation without PETase.

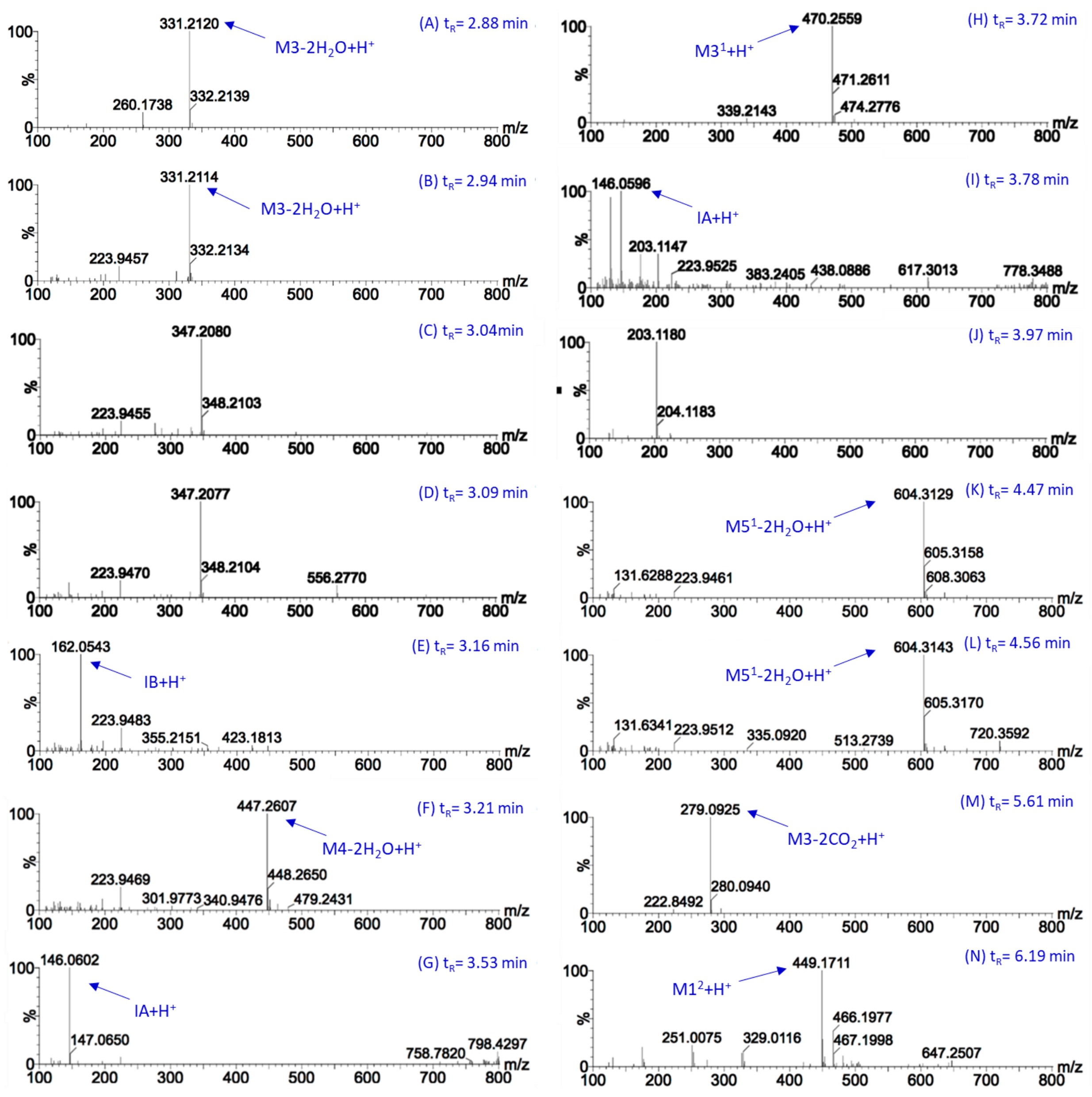

Figure S23. Mass spectra for the fourteen peaks (A-N, for peaks a-n in Fig. 10, respectively) in the base peak chromatograms for BIN after the treatment of PETase. All identified degradation products are marked with blue arrows, and their chemical structures are shown in Fig. S24.

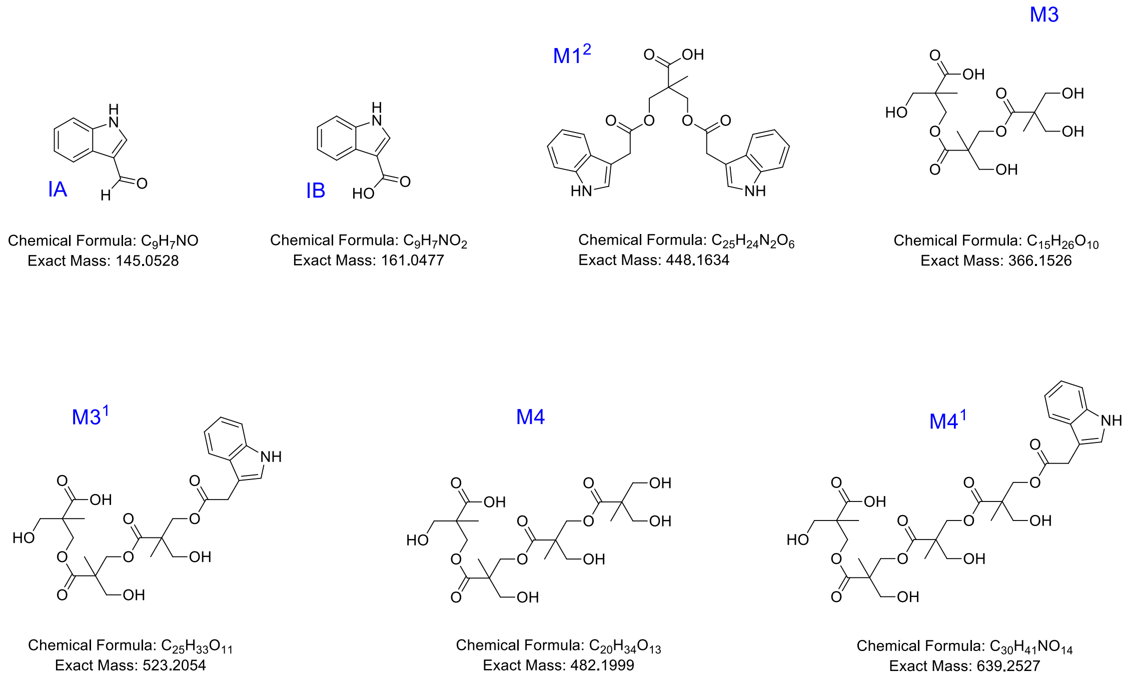

Figure S24. Chemical structures of selected examples of the observed degradation products of BIN (for Fig. S23). Note, possible isomeric structures are not shown.

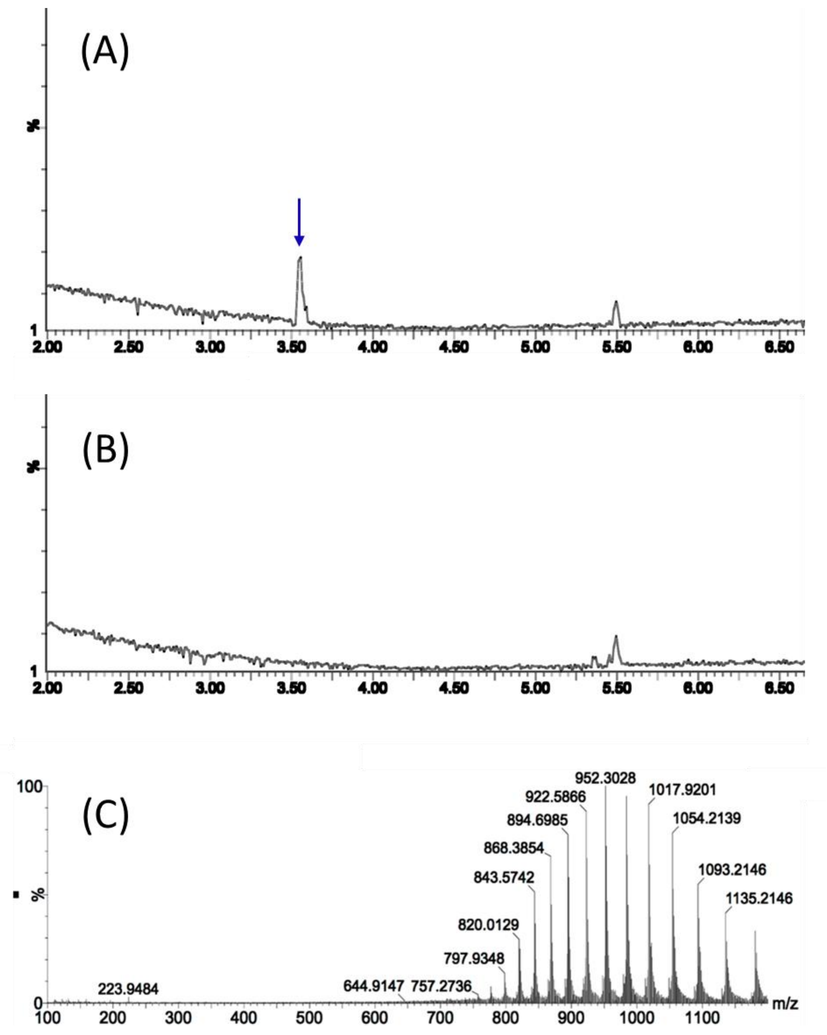

Figure S25. Base peak chromatograms (LC-MS) of the aqueous phase after BISA was reacted with (A) PETase and (B) no enzyme for 3 days. The new signal at 3.56 min in (A) was marked with blue arrow, and its corresponding mass spectrum was shown in (C).

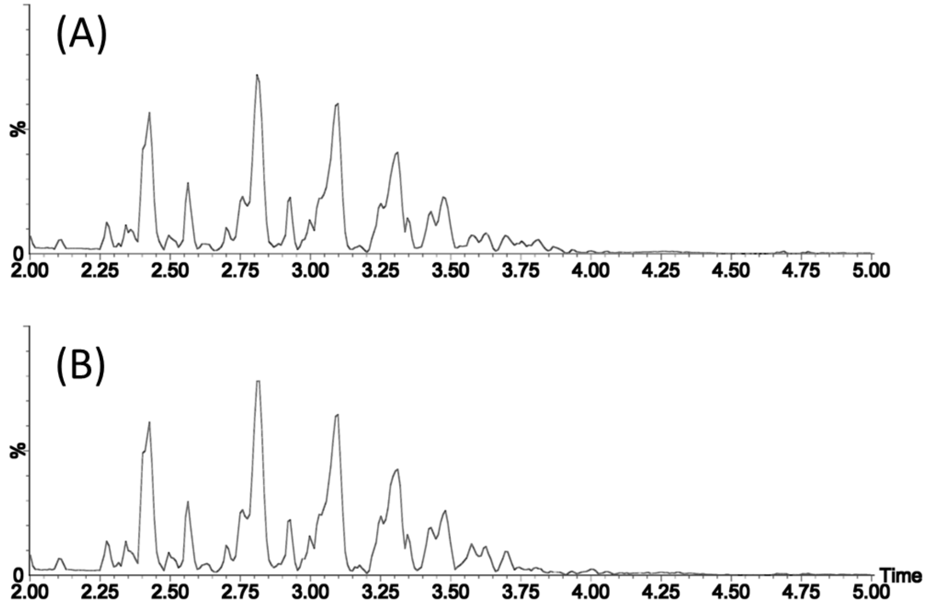

Figure S26. Base peak chromatograms (LC-MS) of the aqueous phase after BH40 was reacted with (A) PETase and (B) no enzyme for 3 days.

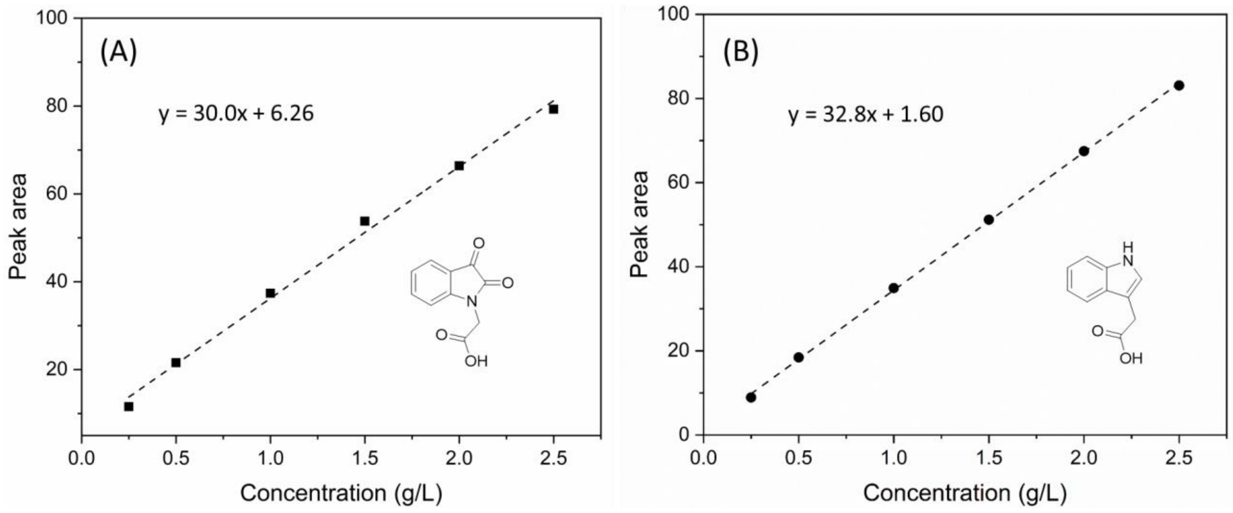

Figure S27. Calibration curve of (A) isatin-N-acetic acid and (B) indole-3-acetic acid standard solution.

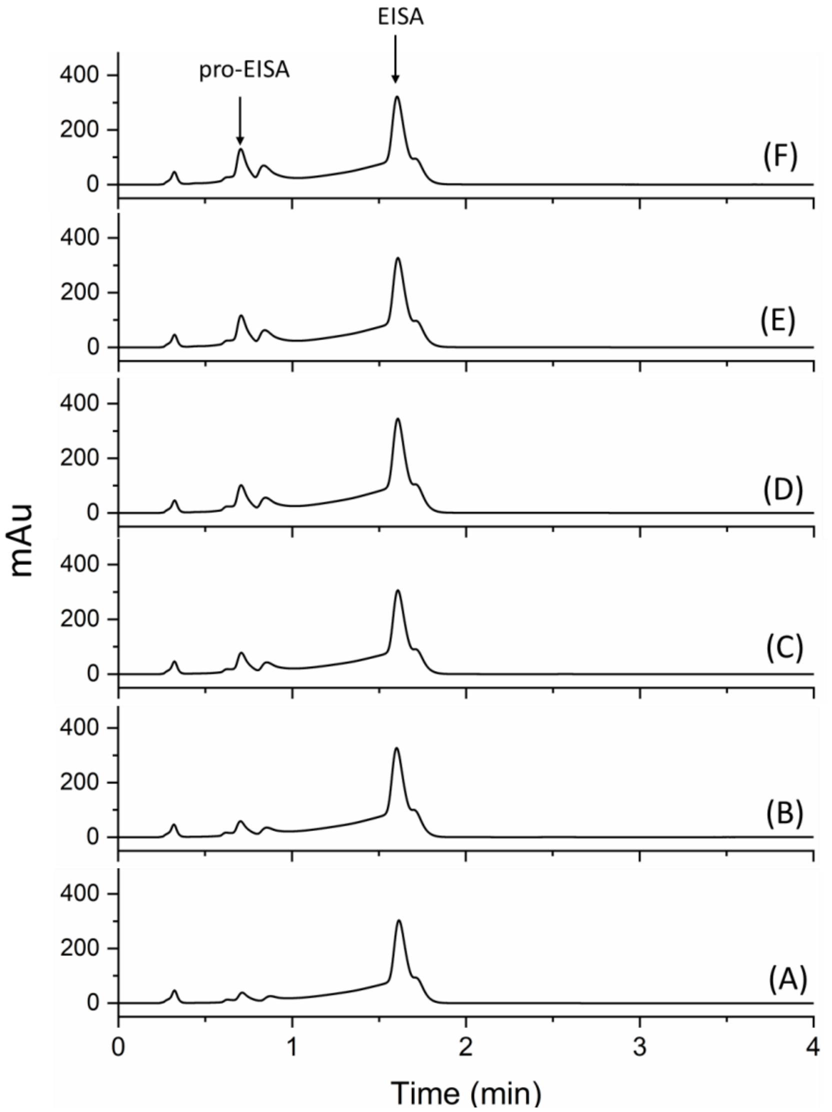

Figure S28. Chromatograms showing the degradation of EISA under PETase negative control condition. The reaction times were: (A) 1; (A) 2; (C) 3; (D) 4; (E) 5 and (D) 6 h.

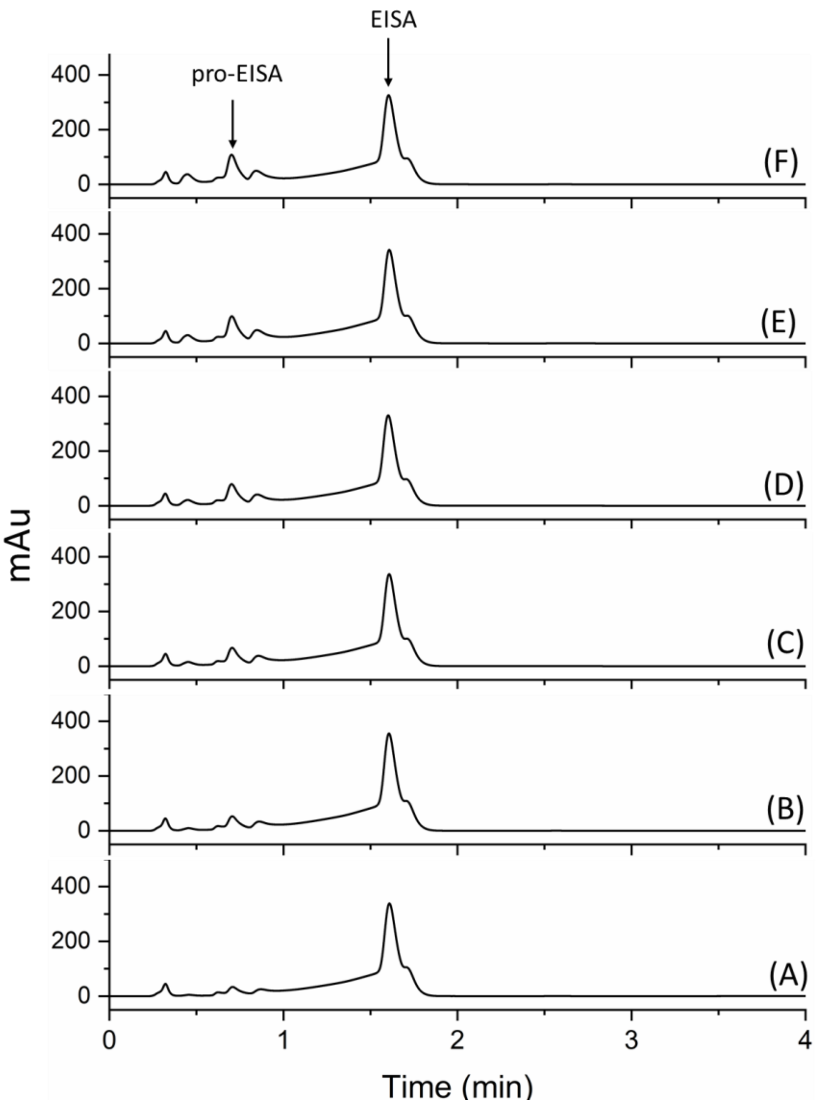

Figure S29. Chromatograms showing the degradation of EISA with PETase. The reaction times were: (A) 1; (A) 2; (C) 3; (D) 4; (E) 5 and (D) 6 h.

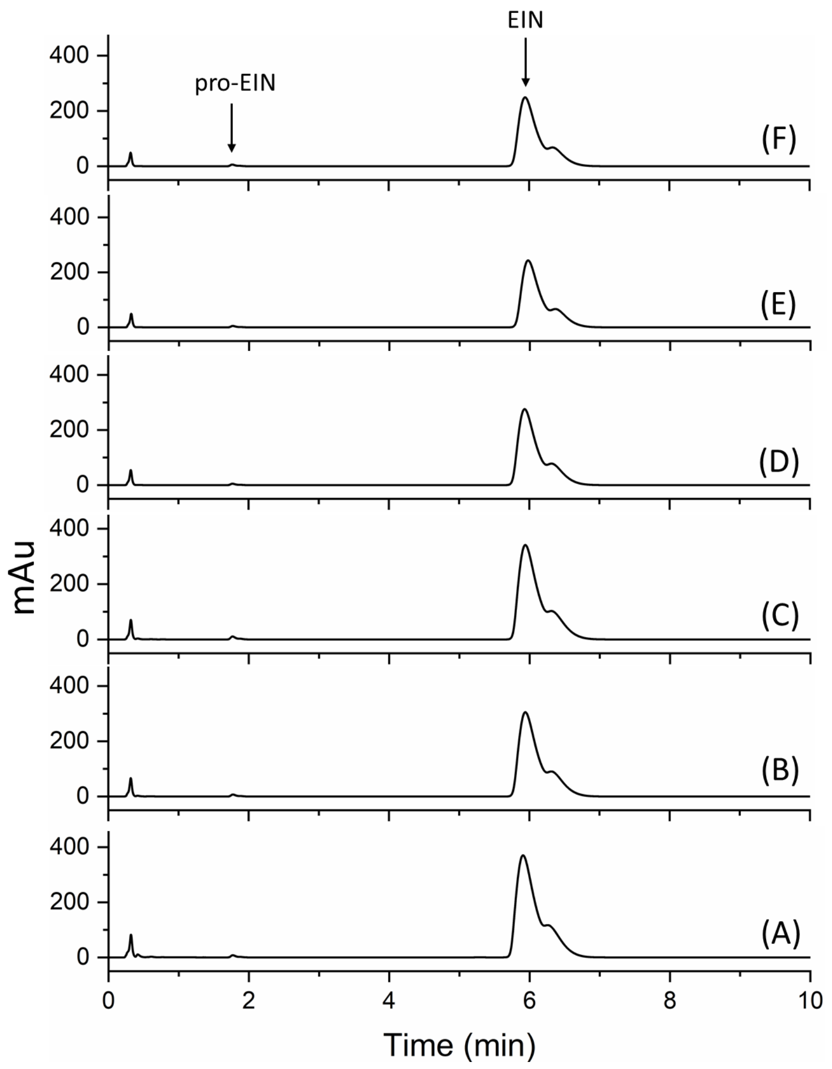

Figure S30. Chromatograms showing the degradation of EIN under PETase negative control condition. The reaction times were: (A) 1; (A) 2; (C) 3; (D) 4; (E) 5 and (D) 6 h.

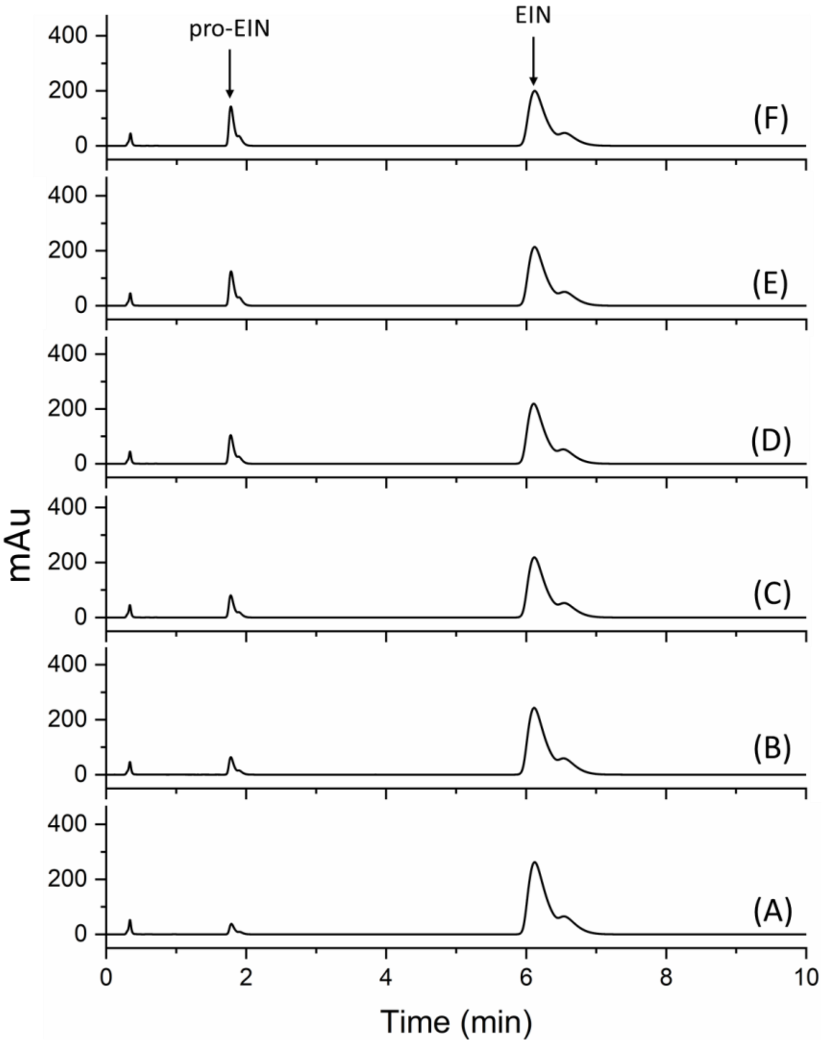

Figure S31. Chromatograms showing the degradation of EIN with PETase. The reaction times were: (A) 1; (A) 2; (C) 3; (D) 4; (E) 5 and (D) 6 h.
